# Six Unprecedented Cytochalasin Derivatives from the Potato Endophytic Fungus *Xylaria curta* E10 and Their Cytotoxicity

**DOI:** 10.3390/ph16020193

**Published:** 2023-01-28

**Authors:** Xian Zhang, Yinzhong Fan, Ke Ye, Xiaoyan Pan, Xujun Ma, Honglian Ai, Baobao Shi, Jikai Liu

**Affiliations:** 1 School of Pharmaceutical Sciences, South-Central Minzu University, Wuhan 430074, China; 2 State Key Laboratory of Phytochemistry and Plant Resources in West China, Kunming Institute of Botany, Chinese Academy of Sciences, Kunming 650201, China

**Keywords:** endophyte fungus, *Xylaria curta*, cytochalasins, isolation and structure elucidation, cytotoxicity

## Abstract

Six previously undescribed cytochalasins, Curtachalasins X1–X6 (**1**–**6**), together with six known compounds (**7**–**12**) were isolated from the endophytic fungus *Xylaria curta* E10 harbored in the plant *Solanum tuberosum*. The structures were elucidated by the interpretation of HRESIMS, UV, and NMR data. The absolute configurations of Curtachalasins X1–X6 were determined by comparison of their experimental and calculated electronic circular dichroism (ECD) spectra. In bioassays, Curtachalasin X1 (**1**) and X5 (**5**) showed cytotoxic activity against the MCF-7 cell line with IC_50_ values of 2.03 μM and 0.85 μM, respectively.

## 1. Introduction

Cytochalasins are a class of cell membrane permeability mycotoxins, and they can lead to disruption of the filament mesh structure, preventing cell movement and changing cell morphology by binding to intracellular filaments and inhibiting actin polymerization at this point [1]. Meanwhile, they are also a large group of fungal polyketide nonribosomal peptide products with remarkable biological activities and structural diversity [2]. Recently, bioactive cytochalasins with various skeletons keep springing up, indicating they are a hot spot of natural product research [3,4,5].

**Figure 1:**
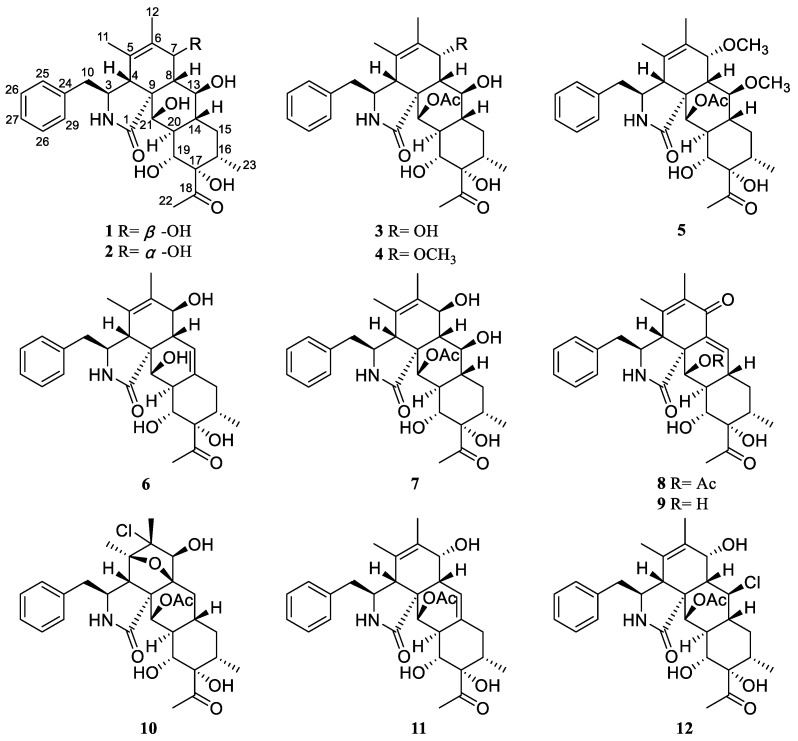
Chemical structures of compounds **1**–**12**.

*Xylaria* was the largest genus of the family Xylariaceae. In the traditional view of this genus, they were saprotrophic fungi that usually appeared on deadwood, participated in the decomposition of organic waste, and even destructed the growth of plants [6]. At the same time, members of this genus are also commonly found in endophytes of vascular plants, so there are many types and contents of cytochalasin in this genus [7,8]. In addition, the type of bioactive compounds in the genus *Xylaria* also include polyketones [9], alkaloids [10], diphenyl ethers [11], diketopiperazines [12], triterpenoid glycosides [13], alkyl aromatics [14], and cyclic depsipeptides [15], with cytotoxic [16] and antithrombotic activity, acetylcholinesterase (AChE) inhibition, antibacterial activity, and phytotoxic activities [17]. In our previous study on *Xylaria curta*, a series of cytochalasins including Curtachalasins A–P were characterized [18,19,20]. Of these, Curtachalasins A and B possess an unprecedented pyrolidine/perhydroanthracene (5/6/6/6 tetracyclic skeleton) fused-ring system [18]. Considering the potential of *Xylariales* species to produce undescribed specialized metabolites, we were inspired to further investigate the traceable cytochalasins produced in different fermentation conditions. As a result, six unprecedented cytochalasins, Curtachalasins X1–X6, together with six known compounds were isolated from *X. curta* E10 (Figure 1). The undescribed structures were established by extensive spectroscopic methods and ECD calculations. All compounds were evaluated by cytotoxicity assay against MCF-7 cell lines. Herein, we report the isolation, structural determination, and bioactivity of these compounds.

## 2. Results

Compound (**1**) was obtained as a colorless amorphous powder. The molecular formula was deduced as C_28_H_37_NO_7_ from the quasimolecular ion at *m/z* 522.2463 [M + Na]^+^ (calcd for C_28_H_37_NO_7_Na^+^, 522.2462) in HRESIMS, indicating eleven degrees of unsaturation. Analysis of the ^1^H and ^13^C NMR data (Table 1 and Table 2) showed characteristic signals, including a single substituted phenyl at δ_H_ 7.33 (2H, t, *J* = 6.8 Hz, H-26/H-28), 7.32 (2H, d, *J* = 6.8 Hz, H-25/H-29), and 7.24 (1H, t, *J* = 6.8 Hz, H-27); four methyl groups at δ_H_ 2.19 (3H, s, H-22), 1.48 (3H, s, H-12), 1.01 (3H, s, H-11), and 0.66 (1H, d, *J* = 6.6 Hz, H-23); and ten methines including four oxygenated methines (δ_C_ 73.3, 72.9, 71.8, 67.0), two methenes (δ_C_ 43.9, 31.7), two quaternary carbons (δ_C_ 84.6, 52.1), a double bond (δ_C_ 132.8, 126.2), one amide group (δ_C_ 177.1), and one ketone group (δ_C_ 214.8). The established functional groups account for seven of eleven degrees of unsaturation, which indicated that compound **1** possessed a four-ring system. 

**Figure 2:**
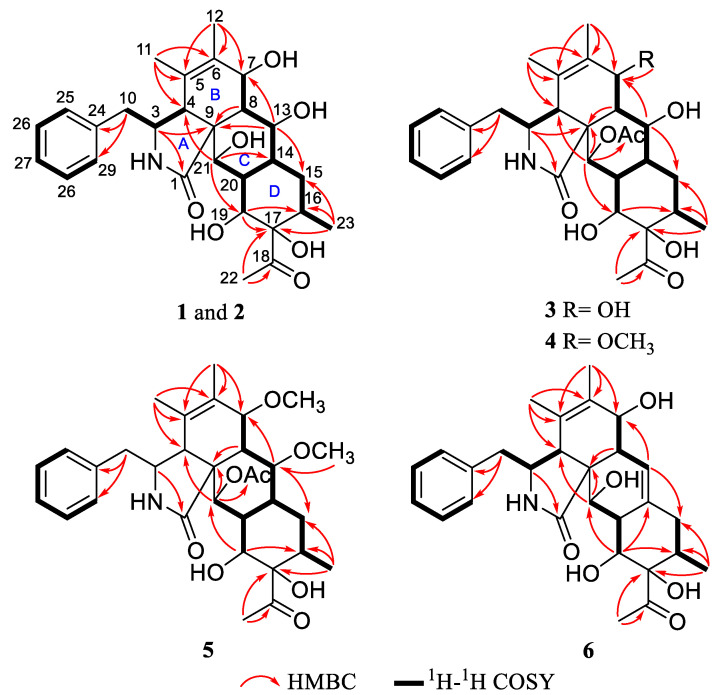
Key HMBC and ^1^H-^1^H COSY correlations of compounds **1**–**6**.

The planar structure of **1** was assigned by extensive NMR data analysis. The ^1^H-^1^H COSY spectrum showed signals of H-10 (*δ*_H_ 2.86)/H-3 (*δ*_H_ 3.12)/H-4 (*δ*_H_ 2.91), H-7(*δ*_H_ 4.01)/H-8 (*δ*_H_ 1.59)/H-13 (*δ*_H_ 4.38)/H-14 (*δ*_H_ 1.52)/H-15 (*δ*_H_ 1.73)/H-16 (*δ*_H_ 1.77)/H-23 (*δ*_H_ 0.66), and H-14 (*δ*_H_ 1.52)/H-20(H-19)/H-21 (*δ*_H_ 4.03), allowing the connections as shown by bold lines in Figure 2. The HBC correlations from H-23 to C-17, from H-19 to C-17 and C-16, and from H-16 to C-19, as well as in combination with the ^1^H-^1^H COSY correlations, established a six-membered ring D. Moreover, the HMBC correlations from H-21 to C-8, C-14, and C-19 and from H-13 to C-9, C-15 and C-20 established a six-membered ring C, which was fused to ring D via C-14 and C-20. According to the HMBC correlations from H-3 to C-1 and C-9, H-11 to C-4, C-5 and C-6, H-8 to C-4, and C-6 and C-9, the fusing pattern of rings A and B can be determined. In addition, the rings B and C of **1** were suggested to be fused through C-9 and C-8 based on the HMBC correlations between H-20 and C-9, between H-21 and C-4, and between H-13 and C-7. Locations of the acetyl group (*δ*_C_ 214.8 and 26.7) at C-17 (*δ*_C_ 84.6) and the phenyl group at C-10 in **1** were determined by HMBC correlations from CH_3_-22 to C-17 and C-18, and from H-10 to C-24 and C-25/29. 

Because the B, C, and D rings are typical cyclohexane structures, the coupling constants between vicinal protons are useful to determine their orientations. The NOESY correlation of H-7/H-13 and H-20/H-21, coupled with the coupling constant of H-7/H-8 (*J*_7,8_ = 10.0 Hz), H-8/H-13 (*J*_8,13_ = 10.0 Hz), H-19/H-20 (*J*_19,20_ = 10.2 Hz), and H-20/H-21 (*J*_20,21_ = 1.8 Hz), implied that H-7, H-13, H-20, and H-21 should be *α*-oriented, and H-8 and H-19 belong to the *β*-orientation. The NOE correlations of 13-OH/H-15a, H-19/H-14, and H-19/H-16 implied that they were cofacial and assigned as *β*-orientated. In contrast, the NOE correlations of H-15b/H-20 and H-15b/17-OH suggested that they were on the same side, with an *α*-orientation. Therefore, the relative configuration was determined as shown in Figure 4.

**Figure 3:**
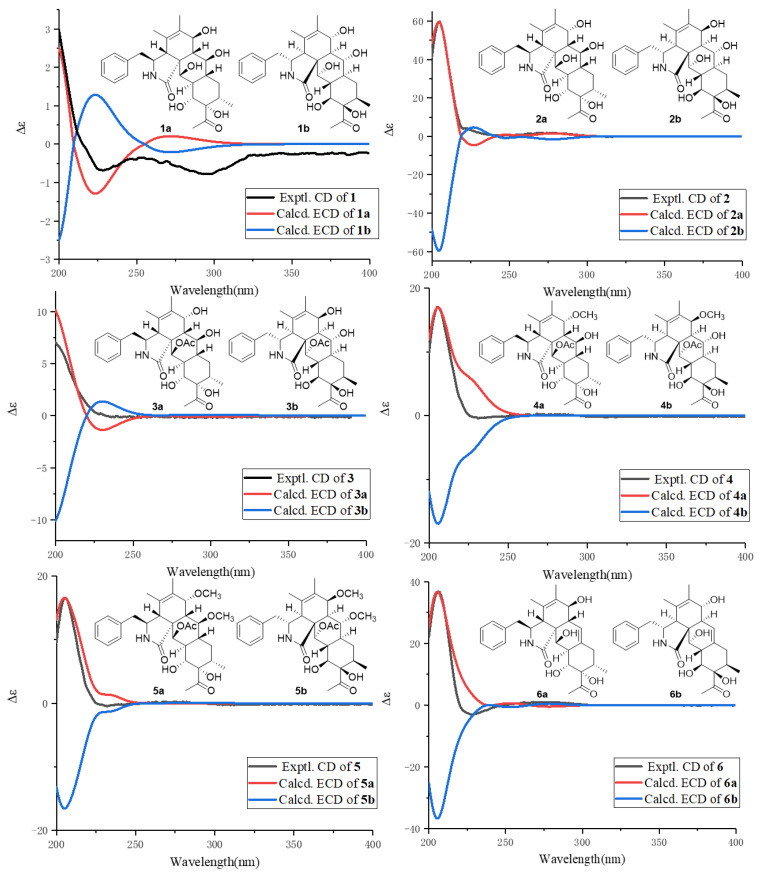
Experimental and calculated ECD curves of compounds **1**–**6**.

The absolute configuration of **1** was determined by ECD calculation (Figure 3) on B3LYP-D3(BJ)/6-311G* (IEFPCM, MeOH) level of theory. The calculated ECD curve of the conformers of **1** with 3*S*, 4*R*, 7*S*, 8*S*, 9*R*, 13*S*, 14*R*, 16*S*, 17*S*, 19*R*, 20*R*, and 21*R*-**1** matched the experimental ECD well. Therefore, compound **1** can be fully assigned to Curtachalasin X1.

**Figure 4:**
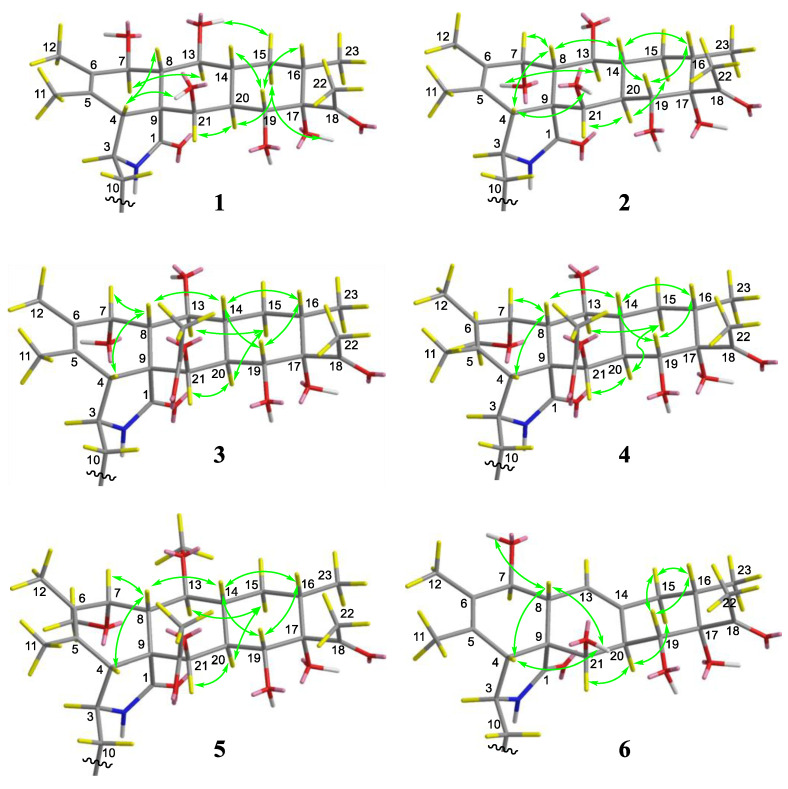
Key NOESY correlations of compounds **1**–**6**.

Compound **2** was obtained as a colorless powder and had the same molecular formula (C_28_H_37_NO_7_) as compound **1**, according to HRESIMS. Their NMR (Table 1 and Table 2) signals were almost identical, except that the coupling constant of H-7/H-8 (3.0 Hz) in **2** was smaller than the coupling constant of H-7/H-8 (10.0 Hz) in **1,** which indicated **2** as an epimer of **1**. In addition, from 2D NMR correlations (Figure 4), the NOE correlations of 7-OH/H-13, and H-7/H-8, coupled with the coupling constant of H-7/H-8 (3.0 Hz) confirmed that H-7 should be *β*-orientated. Finally, to assign the absolute configuration, ECD calculation of **2** was performed at the B3LYP-D3(BJ)/6-311G* (IEFPCM, MeOH) level of theory, and the absolute configuration was deduced as 3*S*, 4*R*, 7*R*, 8*S*, 9*R*, 13*S*, 14*R*, 16*S*, 17*S*, 19*R*, 20*R*, and 21*R*-**2** by comparison of the experimental and calculated ECD data (Figure 3). Therefore, compound **2** can be fully assigned to Curtachalasin X2. 

Compound **3** was obtained as a colorless powder with the molecular formula determined to be C_30_H_39_NO_8_ by HRESIMS at *m*/*z* 564.2585 [M + Na] ^+^ (calcd for C_30_H_39_NO_8_Na^+^, 564.2568). A comparison of the NMR data (Table 1 and Table 2) of **3** with **2** indicated that both compounds share the same skeleton, with the only difference between the two compounds being the presence of an acetyl moiety in **3** instead of a hydroxy in **2**. In the HMBC experiment, the correlation between H-21 (*δ*_H_ 5.50) and acetyl group (*δ*_C_ 172.5) was observed, which suggested that the acetyl group connects to the oxygen at position 21, as shown in Figure 1. The absolute configuration of **3** was assigned as 3*S*, 4*R*, 7*R*, 8*S*, 9*R*, 13*S*, 14*R*, 16*S*, 17*S*, 19*R*, 20*S*, and 21*R* by comparison of the calculated and experimental ECD data (Figure 3), Finally the structure of **3** was established and named as Curtachalasin X3. 

Compound **4** was isolated as a colorless amorphous powder and given a molecular formula of C_31_H_41_NO_8_ by HRESIMS at m/z 578.2724 [M + Na] ^+^ (calcd for C_30_H_39_NO_8_Na^+^, 578.2724). Analysis of the 1D NMR data (Table 1 and Table 3) showed characteristic signals, including a single substituted phenyl at *δ*_H_ 7.32 (2H, t, *J* = 7.2 Hz, H-26/H-28), 7.25 (2H, d, *J* = 7.2 Hz, H-25/H-29), and 7.20 (1H, t, *J* = 7.2 Hz, H-27); five methyl groups (*δ*_C_ 26.1, 21.2, 20.1, 16.8, and 15.4); and ten methines, including four oxygenated methines (*δ*_C_ 78.1, 72.9, 71.5, and 66.1), two methenes (*δ*_C_ 44.2 and 33.4), and a double bond (*δ*_C_ 130.4, 128.6). The above data revealed that **4** was a tetracyclic cytochalasin bearing two acetyl groups, highly similar to those of **3**. The only difference is that the hydroxyl group in **3** is replaced by a methoxyl group in **4**, which was supported by HMBC correlations from -OCH_3_ (*δ*_H_ 3.29) to C-7 (*δ*_C_ 78.1) (Figure 2).

By analysis on the 2D NMR spectra, the coupling constants of H-7/H-8 and NOE for the correlation pattern of **4** are similar to **3**. Thus, the relative configuration of **4** can be deduced, as shown in Figure 4. The absolute configuration of **4** was determined to be 3*S*, 4*R*, 7*R*, 8*S*, 9*R*, 13*S*, 14*R*, 16*S*, 17*S*, 19*R*, 20*S*, and 21*R* by ECD calculation on the same level as for compound **1** (Figure 3). 

Compound **5** was isolated as a colorless powder and given a molecular formula of C_32_H_43_NO_8_ on the basis of the HRESIMS at *m*/*z* 592.2893 [M + Na] ^+^ (calcd for C_32_H_43_NO_8_Na^+^, 592.2881). The NMR spectra of **5** highly matched with those of **4** (Table 1 and Table 3). The only difference is the presence of a methoxyl group at C-13 in **5** instead of 13-OH in **4**, as evidenced by the key HMBC correlations from -OCH_3_ (*δ*_H_ 3.27) to C-13 (*δ*_C_ 80.4). The similar experimental CD spectra (Figure 3) and the coupling constants between H-7 and H-8 in **4** and **5** indicated that they share the identical absolute configuration. The absolute configuration of **5** was determined to be 3*S*, 4*R*, 7*R*, 8*S*, 9*R*, 13*S*, 14*R*, 16*S*, 17*S*, 19*R*, 20*S*, and 21*R*. The identity of the measured circular dichromism (CD) and the calculated ECD spectra of **5** (Figure 3) supported this prediction.

Compound **6** was isolated as a colorless powder. Its molecular formula was deduced as C_28_H_35_NO_6_ by HRESIMS at m/z 482.2540 [M + H] ^+^ (calcd for C_28_H_36_NO_6_^+^, 482.2537). The ^1^H and ^13^C NMR spectra (Table 1 and Table 3) showed that **6** shared similar signals of the mono-substituted phenyl group and tetracyclic skeleton (rings A-D) with compound **1**. For the difference, compound **6** showed one more double bond signal (*δ*_C_ 132.6, 119.9). The ^1^H-^1^H COSY correlation of H-8 (*δ*_H_ 2.20)/H-13 (*δ*_H_ 5.57) together with HMBC correlations from H-13 (*δ*_H_ 5.57) to C-7 (*δ*_C_ 68.1) and C-15 (*δ*_C_ 37.4) suggested that the double bond was located at C-13 (14) (Figure 2). The similar coupling constants between H-7 and H-8 in **1** and **6** indicated that 7-OH should be *β*-oriented. In the NOESY experiment, NOE cross peaks were observed between H-4/H-8, H-16/H-19, and H-4/21-OH, which indicated the relative configuration of **6** as shown in Figure 4. The absolute configuration of **6** was determined to be 3*S*, 4*R*, 7*S*, 8*R*, 9*R*, 16*S*, 17*S*, 19*R*, 20*R*, and 21*R* by ECD calculations at the B3LYP-D3(BJ)/6-311G* (IEFPCM, MeOH) level of theory (Figure 3).

Six known compounds were determined as Curtachalasin J (**7**), Curtachalasin O (**8**), Curtachalasin N (**9**), Curtachalasin I (**10**), Curtachalasin H (**11**), and Curtachalasin K (**12**) by the comparison of their spectral data with the data reported in the literature [20]. 

All compounds were evaluated for their cytotoxicity. As a result, compounds **1** and **5** showed powerful inhibitory activities with IC_50_ values of 2.03 and 0.85 μM, which were more potent than the positive control, cisplatin (IC_50_ = 9.12 μM). Comparing the structures of compounds **1** and **2**, the configuration of the hydroxyl group at the C-7 position may jointly affect the cytotoxic activity. Comparing compounds **4** and **5**, we found that a methoxyl group at C-13 may be a key factor for cytotoxic activity. In addition, the acetylation of OH-21 was also essential for the cytotoxicity of this structure class based on the comparison of **2** with **3** (Table 4). A similar biological property regarding cytotoxic activities has been observed on cytochalasin derivatives previously. For example, xylarichalasin A exhibited moderate cytotoxicity against human cancer cell lines MCF-7 and SMMC-7721 with IC_50_ values of 6.3 and 8.6 µM, respectively. Cytochalasin P1 was also found to have strong cytotoxicity against MCF-7 (IC_50_ 0.71 µM) and SF-268 (1.37 µM) [21].

## 3. Materials and Methods

### 3.1. General Experimental Procedures

Optical rotations were recorded on an Autopol IV polarimeter (Rudolph, Hackettstown, NJ, USA). High-resolution electrospray ionization mass spectra (HRESIMS) were measured on a Q Exactive HF mass spectrometer (Thermo Fisher Scientific, Waltham, Massachusetts, USA). All nuclear magnetic resonance (NMR) spectra were recorded on a Bruker Ascend 600 MHz spectrometer (Bruker Corporation, Karlsruhe, Germany). CD spectra were recorded with an Applied Photophysics spectrometer (Chirascan, New Haven, CT, USA). UV spectra were measured on a UV-2450 spectrometer (Hitachi, Co., Ltd., Tokyo, Japan). Column chromatography (CC) was performed on silica gel (Qingdao Haiyang Chemical Co., Ltd., Qingdao, China) and Sephadex LH-20 (Pharmacia Fine Chemical Co., Ltd., Stockholm, Sweden). Medium-pressure liquid chromatography (MPLC) was performed on a Biotage SP1 equipment, and column packed with C_18_ silica gel. Semipreparative high-performance liquid chromatography (HPLC) experiments were performed on an Agilent 1260 HPLC with an Agilent Zorbax SB-C_18_ column (particle size, 5 μm, i.d. 150 × 20 mm or 150 × 9.8 mm). Fractions were monitored by thin-layer chromatography (TLC) (GF_254_, Qingdao Haiyang Chemical Co., Ltd., Qingdao, China), and spots were visualized by heating silica gel plates sprayed with vanillin and 10% H_2_SO_4_ in EtOH. 

### 3.2. Culture and Fermentation of Fungal Material

*Xylaria curta* E10 was isolated from the healthy stem tissues of potato (*Solanum tuberosum*), which were collected from Dali, Yunnan, China. This isolate was identified according to the ITS sequence (GenBank Accession No. KJ883611.1, query cover 100%, maximum identity 99%). The fungal specimen is deposited at the School of Pharmaceutical Sciences, South-Central Minzu University, Wuhan, China. The strain was fermented by cooked rice medium. The preparation of rice media was 50 g rice with 50 mL water each in 500 mL Erlenmeyer flasks. The fermentation was kept in a dark environment for 30 days at 25 °C (the total weight of rice was 5 kg).

### 3.3. Extraction and Isolation

The rice cultures of *X. curta* E10 (5 kg) were collected and extracted with methanol at room temperature to yield a crude extract after evaporation under vacuum. The crude extract was partitioned between H_2_O and ethyl acetate three times to give an EtOAc extract. The EtOAc extract was concentrated under reduced pressure to give an organic extract (125 g), which was subjected to silica gel column flushing with CHCl_3_-MeOH (1:0-0:1) to obtain five fractions (A-E). Fraction C (43.7 g) was fractionated by MPLC over an RP-18 silica gel column and eluted with MeOH-H_2_O (*v*/*v* 20:80, 40:60, 60:40, 80:20, 100:0, 20 mL/min) to yield eleven subfractions (C1-C11). Fraction C6 (5.6 g) was subjected to a silica gel column gradually eluted with petroleum-acetone (*v*/*v* 20:1, 10:1 5:1, 3:1, 1:1) to give five subfractions (C6-1-C6-5). Fraction C6-2 (760 mg) was further purified by preparative HPLC (with CH_3_CN-H_2_O from 42:52 to 53:47 in 28 min, *v*/*v*, 4.0 mL/min) to obtain compounds **6** (1.6 mg, retention time (*t*_R_) = 22.6 min) and **11** (5.7 mg, *t*_R_ = 25.3 min). Fraction C6-4 was further purified by preparative HPLC (with CH_3_CN-H_2_O from 38:62 to 49:51 in 34 min, *v*/*v*, 4.0 mL/min) to obtain compounds **4** (2.9 mg, *t*_R_ =26.7 min) and **5** (3.7 mg, *t*_R_ = 28.3 min). Fraction C7 (2.8 g) was fractionated by CC over Sephadex LH-20 (CH_3_OH), and then Fraction C7-4 (86 mg) was purified by prep-HPLC (CH_3_CN-H_2_O from 40:60 to 50:50 in 30 min, *v*/*v*, 4.0 mL/min) to give **2** (3.0 mg, *t*_R_ = 25.6 min) and **1** (3.9 mg, *t*_R_ = 26.8 min). Fraction C7-5 (105 mg) was purified by semi-preparative HPLC (CH_3_CN-H_2_O from 37:63 to 46:54 in 30 min) to give **9** (1.3 mg, *t*_R_ = 25.8 min) and **8** (2.2 mg, *t*_R_ = 28.6 min). Fraction C7-6 (74 mg) was further purified by preparative HPLC (with CH_3_CN-H_2_O from 43:57 to 55:45 in 30 min, *v*/*v*, 4.0 mL/min) to obtain compounds **12** (2.7 mg, *t*_R_ = 15.3 min) and **10** (2.1 mg, *t*_R_ = 26.5 min). Fraction C8 (3.2 g) was applied to Sephadex LH-20 eluted with CHCl_3_- CH_3_OH (1:1, *v*/*v*) and was further purified by preparative HPLC (CH_3_CN-H_2_O 43:57, *v/v*, 4.0 mL/min) to obtain compounds **3** (1.3 mg, t*_R_* = 20.6 min) and **7** (6.6 mg, t*_R_* = 21.5 min).

*Curtachalasin X1* (**1**): colorless powder; [*α*${]}_{D}^{20}$ 3.7 (*c* 0.1, CH_3_OH); UV (CH_3_OH) *λ*_max_ (log *ε*) 205 (3.42) nm; ^1^H NMR (600 MHz) and ^13^C NMR (150 MHz, DMSO), see Table 1 and Table 2; HRESIMS (positive) *m*/*z* 522.2463 [M + Na] ^+^ (calcd for C_28_H_37_NO_7_Na^+^, 522.2462). 

*Curtachalasin X2* (**2**): colorless powder; [*α*${]}_{D}^{20}$ 113.3 (*c* 0.1, CH_3_OH); UV (CH_3_OH) *λ*_max_ (log *ε*) 205 (3.47) nm; ^1^H NMR (600 MHz) and ^13^C NMR (150 MHz, DMSO), see Table 1 and Table 2; HRESIMS (positive) *m*/*z* 522.2481 [M + Na] ^+^ (calcd for C_28_H_37_NO_7_Na^+^, 522.2462).

*Curtachalasin X3* (**3**): colorless powder; [*α*${]}_{D}^{22}$ 5.18 (*c* 0.1, CH_3_OH); UV (CH_3_OH) *λ*_max_ (log *ε*) 205 (3.05) nm; ^1^H NMR (600 MHz) and ^13^C NMR (150 MHz, CD_3_OD), see Table 1 and Table 2; HRESIMS (positive) *m*/*z* 564.2585 [M + Na]^+^ (calcd for C_30_H_39_NO_8_Na^+^, 564.2568).

*Curtachalasin X4* (**4**): colorless powder; [*α*${]}_{D}^{20}$ 36.4 (*c* 0.1, CH_3_OH); UV (CH_3_OH) *λ*_max_ (log *ε*) 205 (3.88) nm; ^1^H NMR (600 MHz) and ^13^C NMR (150 MHz, DMSO), see Table 1 and Table 3; HRESIMS (positive) *m*/*z* 578.2724 [M + Na]^+^ (calcd for C_30_H_39_NO_8_Na^+^, 578.2724). 

*Curtachalasin X5* (**5**): colorless powder; [*α*${]}_{D}^{21}$ 73.6 (*c* 0.1, CH_3_OH); UV (CH_3_OH) *λ*_max_ (log *ε*) 210 (3.43) nm; ^1^H NMR (600 MHz) and ^13^C NMR (150 MHz, CD_3_OD), see Table 1 and Table 3; HRESIMS (positive) *m*/*z* 592.2893 [M + Na]^+^ (calcd for C_32_H_43_NO_8_Na^+^, 592.2881). 

*Curtachalasin X6* (**6**): colorless powder; [*α*${]}_{D}^{20}$ 28.0 (*c* 0.1, CH_3_OH); UV (CH_3_OH) *λ*_max_ (log *ε*) 205 (3.43) nm; ^1^H NMR (600 MHz) and ^13^C NMR (150 MHz, DMSO), see Table 1 and Table 3; HRESIMS (positive) *m*/*z* 482.2540 [M + H]^+^ (calcd for C_28_H_36_NO_6_^+^, 482.2537).

### 3.4. Quantum Chemical Calculations

Theoretical calculation of ECD spectra for compounds **1**–**6** were performed with the Gaussian 16 program package. Conformational analysis was initially performed using Spartan 14. The optimized conformation geometries, thermodynamic parameters, and populations of all conformations are provided in Tables S7–S18 in the Supporting Information. The conformers were first identified using the time-dependent density functional theory (TDDFT) method at the B3LYP-D3(BJ)/6-311G* level, and the frequency was calculated at the same level of theory. Then, the theoretical calculations of ECD were performed using TDDFT at B3LYP-D3(BJ)/6-311G* level with PCM in methanol. The final ECD spectra were obtained according to the Boltzmann calculated contribution of each conformer after UV correction.

### 3.5. Cytotoxicity Assay against MCF-7

The cytotoxicity actions of the pure isolated compounds were tested on the breast cancer MCF-7 cell lines using the MTS assay. For this, cells were cultured in DMEM medium, supplemented with 10% fetal bovine serum FBS, 100 U/mL penicillin, and 100 U/mL streptomycin (Invitrogen, Carlsbad, CA, USA), and incubated at 37 °C, 5% CO_2_ until 70–80% coverage. Cells were then transferred into 96-well culture plates with appropriate density. The plate was incubated for another 24 h at 37 °C, 5% CO_2_ for cell growth and adhesion. Then, the cells were treated with test samples prepared in culture media at different concentrations (0.08, 0.16, 0.31, 0.62, 1.25, 2.5, 5, 10, 20, and 40 μM) for 48 h. The blank control (wells with MTS, without cells) and the negative control (wells with solution, without samples) were performed simultaneously. After 48 h, 20 μL of MTS reagent was added into each well and optical density at 490 nm was read using a Spectra Max M5 microplate reader (Molecular Devices, Sunnyvale, CA, USA) after 1 h incubation at 37 °C, 5% CO_2_, in the dark. The percentage of cell inhibition was calculated based on the equation below.

$$\%\;Inhibition=\left(1-\frac{Absorbance\;\left(sample\right)-Absorbance\;\left(blank\right)}{Absorbance\;\left(negative\right)-Absorbance\;\left(blank\right)}\right)\times 100\%$$

## 4. Conclusion

In summary, the structures of six undescribed cytochalasins with a tetracyclic skeleton (**1**–**6**) were determined unambiguously by extensive spectroscopic analysis, with the absolute configuration being determined by quantum chemistry calculations. In the cytotoxicity assay, compounds **1** and **5** showed activity against MCF-7 cell lines with outstandingly low IC_50_ values (2.03 and 0.85 μM) compared to the positive anti-cancer drug cisplatin. Our studies have certain significances in exploring the structural diversity of cytochalasins.

## Figures and Tables

**Table 1:** The ^13^C NMR data (150 MHz) of compounds **1**–**6**.

No.	1 ^a^	2 ^a^	3 ^b^	4 ^a^	5 ^b^	6 ^a^
1	177.1, C	178.4, C	178.7, C	174.7, C	178.7, C	176.1, C
3	59.4, CH	58.5, CH	61.2, CH	59.9, CH	61.5, CH	58.5, CH
4	47.2, CH	47.2, CH	49.5, CH	47.9, CH	49.3, CH	47.3, CH
5	126.2, C	128.3, C	130.4, C	128.6, C	129.6, C	126.8, C
6	132.8, C	130.7, C	131.4, C	130.4, C	133.0, C	134.7, C
7	71.8, CH	66.7, CH	68.0, CH	78.1, CH	77.5, CH	68.1, CH
8	43.4, CH	44.0, CH	46.6, CH	46.9, CH	46.6, CH	39.2, CH
9	52.1, C	50.4, C	50.6, C	50.0, C	50.8, C	50.5, C
10	43.9, CH_2_	44.5, CH_2_	44.8, CH_2_	44.2, CH_2_	45.3, CH_2_	44.1, CH_2_
11	17.0, CH_3_	18.5, CH_3_	17.1, CH_3_	16.8, CH_3_	17.3, CH_3_	17.3, CH_3_
12	14.2, CH_3_	16.8, CH_3_	18.4, CH_3_	20.1, CH_3_	21.1, CH_3_	15.1, CH_3_
13	73.3, CH	67.8, CH	69.3, CH	66.1, CH	80.4, CH	119.9, CH
14	39.1, CH	39.7, CH	42.1, CH	42.4, CH	41.4, CH	132.6, C
15	31.7, CH_2_	32.4, CH_2_	33.0, CH_2_	33.4, CH_2_	32.9, CH_2_	37.4, CH_2_
16	35.9, CH	36.1, CH	37.3, CH	35.5, CH	37.3, CH	35.7, CH
17	84.6, C	84.7, C	85.7, C	84.3, C	85.6, C	84.8, C
18	214.8, C	215.8, C	215.7, C	214.9, C	215.4, C	214.8, C
19	72.9, CH	73.2, CH	74.1, CH	72.9, CH	74.0, CH	72.3, CH
20	41.3, CH	41.7, CH	42.1, CH	41.5, CH	42.2, CH	43.1, CH
21	67.0, CH	67.8, CH	73.3, CH	71.5, CH	73.2, CH	67.4, CH
22	26.7, CH_3_	26.7, CH_3_	26.3, CH_3_	26.1, CH_3_	26.2, CH_3_	26.6, CH_3_
23	15.7, CH_3_	15.7, CH_3_	15.3, CH_3_	15.4, CH_3_	15.4, CH_3_	15.3, CH_3_
24	138.7, C	138.7, C	138.7, C	138.5, C	139.2, C	138.8, C
25, 29	130.0, CH	130.1, CH	130.6, CH	129.8, CH	130.7, CH	130.0, CH
26, 28	128.8, CH	128.8, CH	129.6, CH	128.9, CH	130.3, CH	128.8, CH
27	126.8, CH	126.8, CH	127.7, CH	126.9, CH	127.7, CH	126.8, CH
21-OAc			172.5, C	170.4, C	172.5, C	
			20.9, CH_3_	21.2, CH_3_	20.8, CH_3_	
7-OCH_3_				59.3	59.8	
13-OCH_3_					57.5	

^a^ in DMSO, ^b^ in CD_3_OD.

**Table 2:** The ^1^H NMR data (600 MHz) of compounds **1**–**3** (*δ* in ppm, *J* in Hz).

No.	1 ^a^	2 ^a^	3 ^b^
3	3.12, m	3.15, m	3.09, m
4	2.91, br s	2.87, br s	2.39, br s
7	4.01, d, (10.0)	3.93, dd, (5.0, 3.0)	4.19, d, (2.4)
8	1.59, dd, (10.0, 10.0)	1.56, d, (3.0)	1.63, d, (11.0, 2.4)
10	2.86, dd, (12.8, 5.3)	2.86, dd, (12.8, 4.8)	2.98, dd, (13.1, 5.2)
	2.74, dd, (12.8, 9.6)	2.67, dd, (12.8, 10.0)	2.90, dd, (13.1, 9.2)
11	1.01, s	1.56, s	1.04, s
12	1.48, s	0.93, s	1.69, s
13	4.38, dd, (10.0, 3.7)	4.18, t, 10.4	4.26, dd, (11.0, 10.0)
14	1.52, m	1.41, ddd, (12.0, 9.2, 2.8)	1.55, m
15	1.73, m	1.80, m	1.95, dt, (12.8, 3.7)
	1.01, m	1.05, dd, (12.0, 12.0)	1.30, m
16	1.77, m	1.77, m	1.89, m
19	3.76, dd, (10.2, 8.8)	3.75, dd, (10.6, 8.8)	3.50, d, (10.6)
20	1.99, ddd, (10.2, 10.2,1.8)	2.21, dd, (10.6, 2.0)	2.69, ddd, (12.0, 10.6, 2.2)
21	4.03, d, (6.0, 1.8)	3.90, dd, (5.8, 2.0)	5.50, d, (2.2)
22	2.19, s	2.19, s	2.25, s
23	0.66, d, (6.6)	0.66, d, (6.4)	0.77, d, (6.8)
25, 29	7.32, d, (6.8)	7.32, d, (7.1)	7.26, d, (7.2)
26, 28	7.33, t, (6.8)	7.32, t, (7.1)	7.30, t, (7.2)
27	7.24, t, (6.8)	7.23, t, (7.1)	7.20, t, (7.2)
21-OAc			2.20, s
7-OH	5.95, d, (2.9)	3.51, d, (5.0)	
13-OH	5.56, d, (3.7)	4.10, d, (6.8)	
17-OH	4.40, s	4.34, s	
19-OH	4.54, d, (8.8)	4.54, d, (8.8)	
21-OH	5.14, d, (6.0)	5.13, d, (5.8)	

^a^ in DMSO, ^b^ in CD_3_OD.

**Table 3:** The ^1^H NMR data (600 MHz) of compounds **4**–**6** (*δ* in ppm, *J* in Hz).

No.	4 ^a^	5 ^b^	6 ^a^
3	3.15, m	3.32, m	3.11, m
4	2.27, br s	2.39, br s	2.80, br s
7	3.91, d, (2.0)	3.75, d, (1.8)	3.61, dd, (9.8, 7.7)
8	1.98, dd, (11.2, 2.0)	1.82, dd, (10.8, 1.8)	2.20, m
10	2.87, dd, (13.0, 4.2)	2.97, dd, (13.1, 5.2)	2.88, dd, (12.8, 5.3)
	2.78, dd, (13.0, 9.8)	2.84, dd, (13.1, 9.2)	2.76, dd, (12.8, 9.7)
11	0.85, s	1.03, s	1.03, s
12	1.66, s	1.78, s	1.52, s
13	5.06, dd, (11.0, 11.0)	4.24, dd, (10.8, 10.8)	5.57, d, (2.1)
14	1.76, dd, (11.0, 2.3)	1.57, m	
15	1.91, m	1.83, m	2.07, m
	1.21, d, (11.7)	1.36, dd, (12.2, 12.2)	1.86, m
16	1.88, m	1.88, m	1.82, m
19	3.37, m	3.48, d, (9.7)	3.94, dd, (10.6, 8.6)
20	2.75, m	2.77, ddd, (10.0, 10.0, 2.3)	2.45, m
21	5.41, d, (2.0)	5.42, d, (2.3)	4.18, dd, (6.4, 3.7)
22	2.18, s	2.25, s	2.20, s
23	0.68, d, (6.5)	0.77, d, (6.6)	0.66, d, (6.6)
25, 29	7.25, d, (7.2)	7.29, d, (7.0)	7.32, d, (7.2)
26, 28	7.32, t, (7.2)	7.28, t, (7.0)	7.33, t, (7.2)
27	7.20, t, (7.2)	7.20, t, (7.0)	7.24, t, (7.2)
21-OAc	2.14, s	2.16, s	
7-OCH_3_	3.29, s	3.48, s	
13-OCH_3_		3.27, s	
7-OH			4.74, d, (7.7)
17-OH			4.50, s
19-OH			4.70, d, (8.6)
21-OH			5.20, d, (6.4)

^a^ in DMSO, ^b^ in CD_3_OD.

**Table 4:** The cytotoxic activities of isolated compounds (IC_50_, μM).

Compound	MCF-7	Compound	MCF-7
**1**	2.03 ± 0.63	**8**	>40
**2**	>40	**9**	>40
**3**	13.86 ± 1.62	**10**	>40
**4**	>40	**11**	>40
**5**	0.85 ± 0.13	**12**	>40
**6**	>40	cisplatin	9.12 ± 0.41
**7**	>40		

## Data Availability

Data are contained within the article and Supplementary Materials.

## Supplementary Materials

The following are available online at https://www.mdpi.com/article/10.3390/ph16020193/s1, Figure S1.1: ^1^H NMR spectrum of **1** in DMSO-*d_6_*, Table S1: Cartesian coordinates for the low-energy optimized conformers of **1a** at B3LYP-D3(BJ)/6-311G* level.
