# Supplementary material for: Six Unprecedented Cytochalasin Derivatives from the Potato Endophytic Fungus Xylaria curta E10 and Their Cytotoxicity
Source: Pharmaceuticals (Basel). 2023 Jan 28;16(2):193. doi: 10.3390/ph16020193 (PMC9964435; doi:10.3390/ph16020193)
Supplement: Supplementary file 1 [file pharmaceuticals-16-00193-s001.zip › pharmaceuticals-2143089-supplementary.pdf]

Supplementary materials for

Six unprecedented Cytochalasins Derivatives from the Potato Endophytic Fungus *Xylaria curta* E10 and Their Cytotoxicity

Xian Zhang,‡[a,b] Yin-Zhong Fan,‡[a] Ke Ye,[a] Xiao-Yan Pan,[a] Xu-Jun Ma,[a] Hong-Lian Ai,*[a,b] Bao-Bao Shi,*[a,b] and Ji-Kai Liu*[a,b]

[a]School of Pharmaceutical Sciences, South-Central MinZu University, Wuhan 430074, People's Republic of China;

[b]State Key Laboratory of Phytochemistry and Plant Resources in West China, Kunming Institute of Botany, Chinese Academy of Sciences

*Corresponding Author.

E-mail: aihonglian05@163.com (H.-L. A.); shibb0505@163.com (B.-B. S.); liujikai@mail.scuec.edu.cn (J. K. Liu)

‡These authors contributed equally to this work.

Table of Contents

Spectroscopic data 4
Figure S1.1 ^{1}H NMR spectrum of **1** in DMSO-d_6 4
Figure S1.2 ^{13}C NMR spectrum of **1** in DMSO-d_6 4
Figure S1.3 HSQC spectrum of **1** in DMSO-d_6 5
Figure S1.4 HMBC spectrum of **1** in DMSO-d_6 5
Figure S1.5 COSY spectrum of **1** in DMSO-d_6 6
Figure S1.6 ROESY spectrum of **1** in DMSO-d_6 6
Figure S1.7 HRMS spectrum of **1** 7
Figure S2.1 ^{1}H NMR spectrum of **2** in DMSO-d_6 8
Figure S2.2 ^{13}C NMR spectrum of **2** in DMSO-d_6 8
Figure S2.3 HSQC spectrum of **2** in DMSO-d_6 9
Figure S2.4 HMBC spectrum of **2** in DMSO-d_6 9
Figure S2.5 COSY spectrum of **2** in DMSO-d_6 10
Figure S2.6 Roesy spectrum of **2** in DMSO-d_6 10
Figure S2.7 HRMS spectrum of **2** 11
Figure S3.1 ^{1}H NMR spectrum of **3** in CD_3OD 12
Figure S3.2 ^{13}C NMR spectrum of **3** in CD_3OD 12
Figure S3.3 HSQC spectrum of **3** in CD_3OD 13
Figure S3.4 HMBC spectrum of **3** in CD_3OD 13
Figure S3.5 COSY spectrum of **3** in CD_3OD 14
Figure S3.6 Roesy spectrum of **3** in CD_3OD 14
Figure S3.7 HRMS spectrum of **3** 15
Figure S4.1 ^{1}H NMR spectrum of **4** in CD_3OD 16
Figure S4.2 ^{13}C NMR spectrum of **4** in CD_3OD 16
Figure S4.3 HSQC spectrum of **4** in CD_3OD 17
Figure S4.4 HMBC spectrum of **4** in CD_3OD 17
Figure S4.5 COSY spectrum of **4** in CD_3OD 18
Figure S4.6 Roesy spectrum of **4** in CD_3OD 18
Figure S4.7 ^{1}H NMR spectrum of **4** in DMSO-d_6 19
Figure S4.8 ^{13}C NMR spectrum of **4** in DMSO-d_6 19
Figure S4.9 HSQC spectrum of **4** in DMSO-d_6 20
Figure S4.10 HMBC spectrum of **4** in DMSO-d_6 20
Figure S4.11 COSY spectrum of **4** in DMSO-d_6 21
Figure S4.12 Roesy spectrum of **4** in DMSO-d_6 21
Figure S4.13 HRMS spectrum of **4** 22
Figure S5.1 ^{1}H NMR spectrum of **5** in CD_3OD 23
Figure S5.2 ^{13}C NMR spectrum of **5** in CD_3OD 23
Figure S5.3 HSQC spectrum of **5** in CD_3OD 24
Figure S5.4 HMBC spectrum of **5** in CD_3OD 24
Figure S5.5 COSY spectrum of **5** in CD_3OD 25
Figure S5.6 Roesy spectrum of **5** in CD_3OD 25
Figure S5.7 HRMS spectrum of **5** 26
Figure S6.1 ^{1}H NMR spectrum of **6** in DMSO-d_6 27

Figure S6.2 ^{13}C NMR spectrum of **6** in DMSO-d_6 27
Figure S6.3 HSQC spectrum of **6** in DMSO-d_6 28
Figure S6.4 HMBC spectrum of **6** in DMSO-d_6 28
Figure S6.5 COSY spectrum of **6** in DMSO-d_6 29
Figure S6.6 Roesy spectrum of **6** in DMSO-d_6. 29
Quantum chemical calculation 31
Figure S1. ECD calculations of **1−6**. 31
Table S1. Cartesian coordinates for the low-energy optimized conformers of **1a** at B3LYP-D3(BJ)/6-311G* level 32
Table S2. Cartesian coordinates for the low-energy optimized conformers of **2a** at B3LYP-D3(BJ)/6-311G* level. 42
Table S3. Cartesian coordinates for the low-energy optimized conformers of **3a** at B3LYP-D3(BJ)/6-311G* level. 49
Table S4. Cartesian coordinates for the low-energy optimized conformers of **4a** at B3LYP-D3(BJ)/6-311G* level. 56
Table S5. Cartesian coordinates for the low-energy optimized conformers of **5a** at B3LYP-D3(BJ)/6-311G* level. 62
Table S6. Cartesian coordinates for the low-energy optimized conformers of **6a** at B3LYP-D3(BJ)/6-311G* level. 69
Table S7. Important thermodynamic parameters of the M062X/Def2SVP optimized conformers of **1a** in the gas phase 76
Table S8. Conformational analysis of the M062X/Def2SVP optimized conformers of **1a** in the gas phase (T=298.15 K) 76
Table S9. Important thermodynamic parameters of the B3LYP/6-311G* optimized conformers of **2a** in the gas phase 77
Table S10. Conformational analysis of the B3LYP/6-311G* optimized conformers of **2a** in the gas phase (T=298.15 K) 77
Table S11. Important thermodynamic parameters of the B3LYP/6-311G* optimized conformers of **3a** in the gas phase 78
Table S12. Conformational analysis of the B3LYP/6-311G* optimized conformers of **3a** in the gas phase (T=298.15 K) 78
Table S13. Important thermodynamic parameters of the B3LYP/6-311G* optimized conformers of **4a** in the gas phase 79
Table S14. Conformational analysis of the B3LYP/6-311G* optimized conformers of **4a** in the gas phase (T=298.15 K) 79
Table S15. Important thermodynamic parameters of the B3LYP/6-311G* optimized conformers of **5a** in the gas phase 80
Table S16. Conformational analysis of the B3LYP/6-311G* optimized conformers of **5a** in the gas phase (T=298.15 K) 80
Table S17. Important thermodynamic parameters of the B3LYP/6-311G* optimized conformers of **6a** in the gas phase 81
Table S18. Conformational analysis of the B3LYP/6-311G* optimized conformers of **6a** in the gas phase (T=298.15 K) 81

Spectroscopic data

Figure S1.1 ^{1}H NMR spectrum of **1** in DMSO-d_6

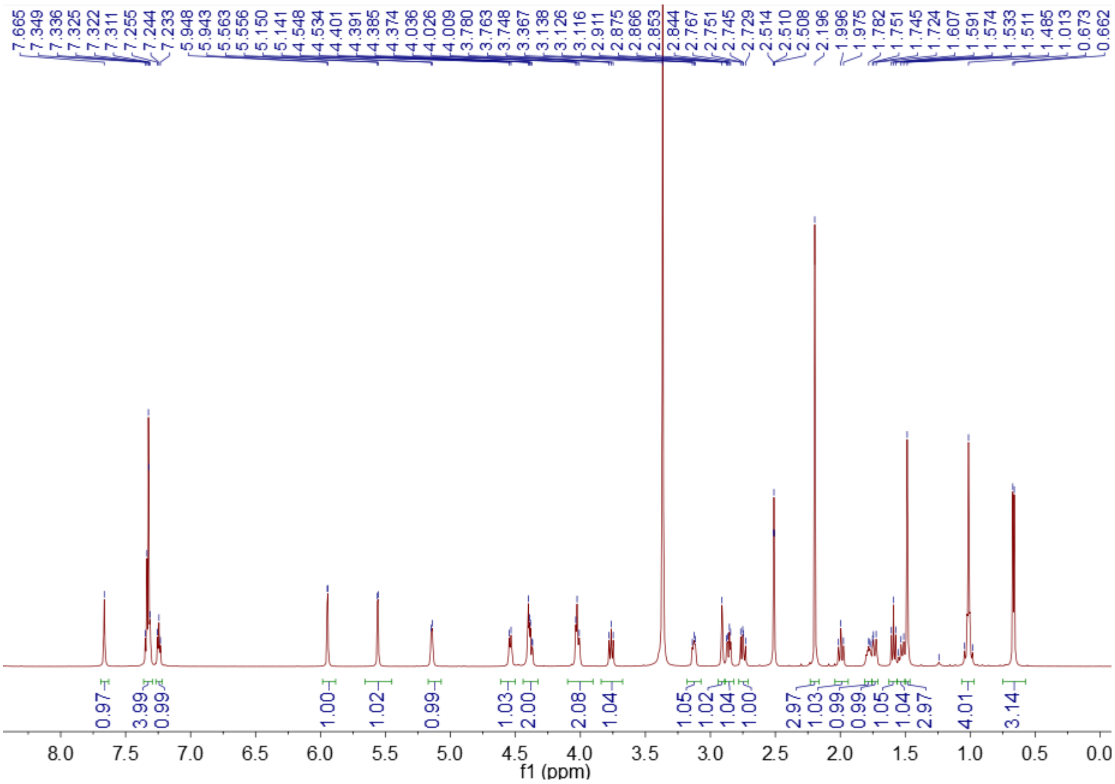

Figure S1.2 ^{13}C NMR spectrum of **1** in DMSO-d_6

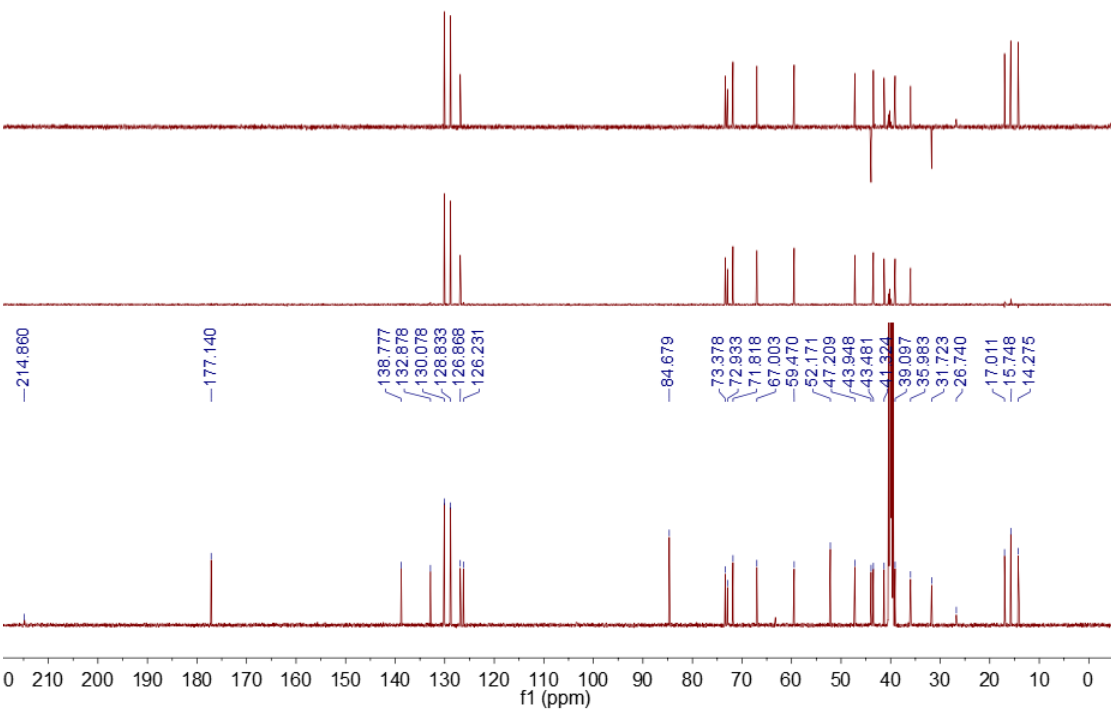

Figure S1.3 HSQC spectrum of **1** in DMSO-d_6

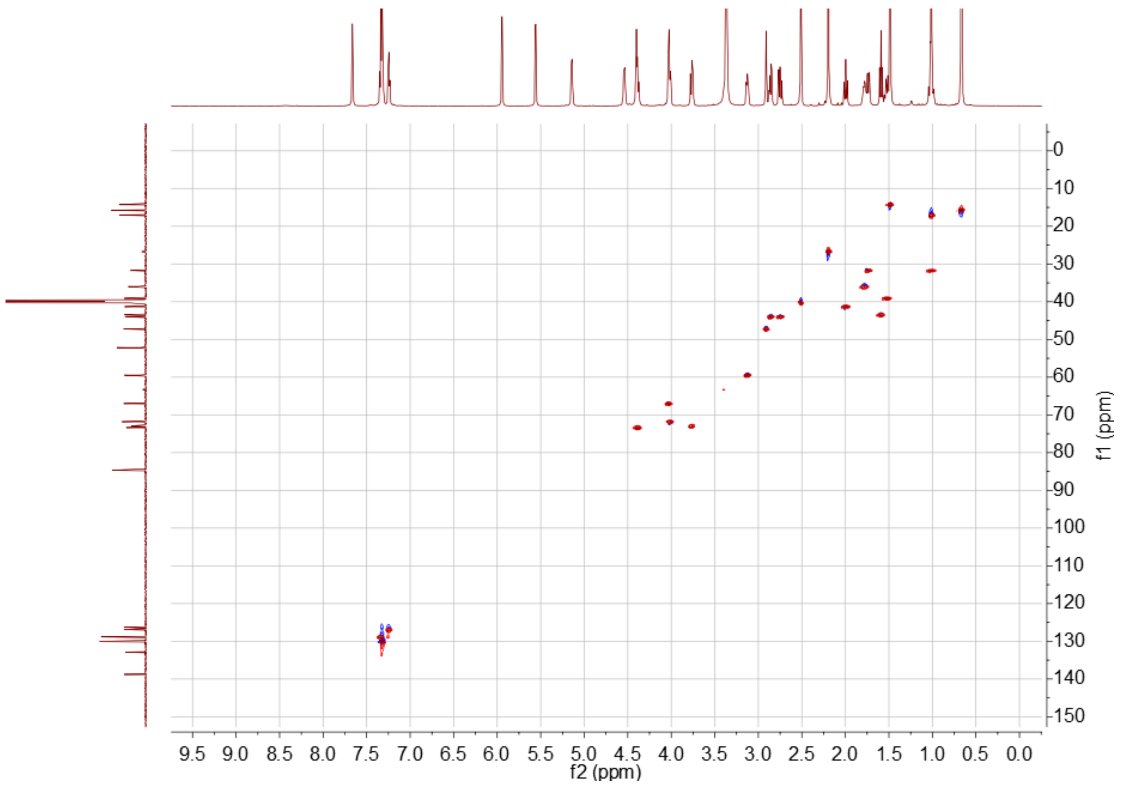

Figure S1.4 HMBC spectrum of **1** in DMSO-d_6

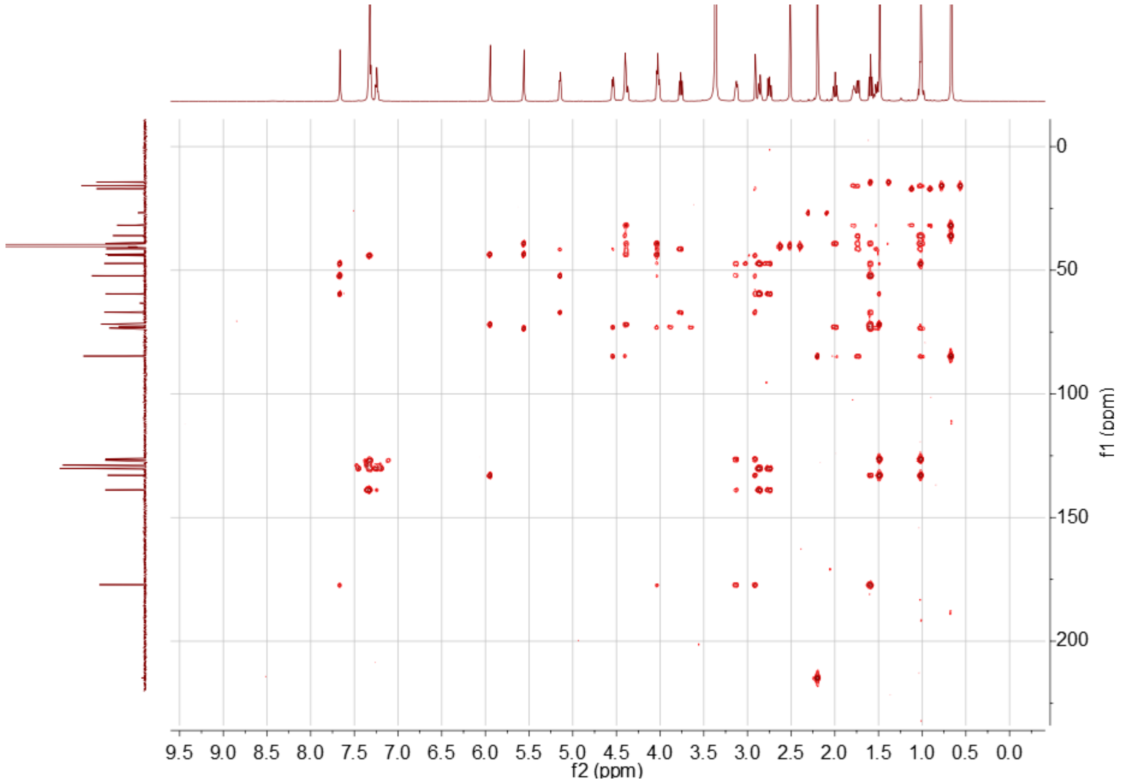

Figure S1.5 COSY spectrum of **1** in DMSO-d_6

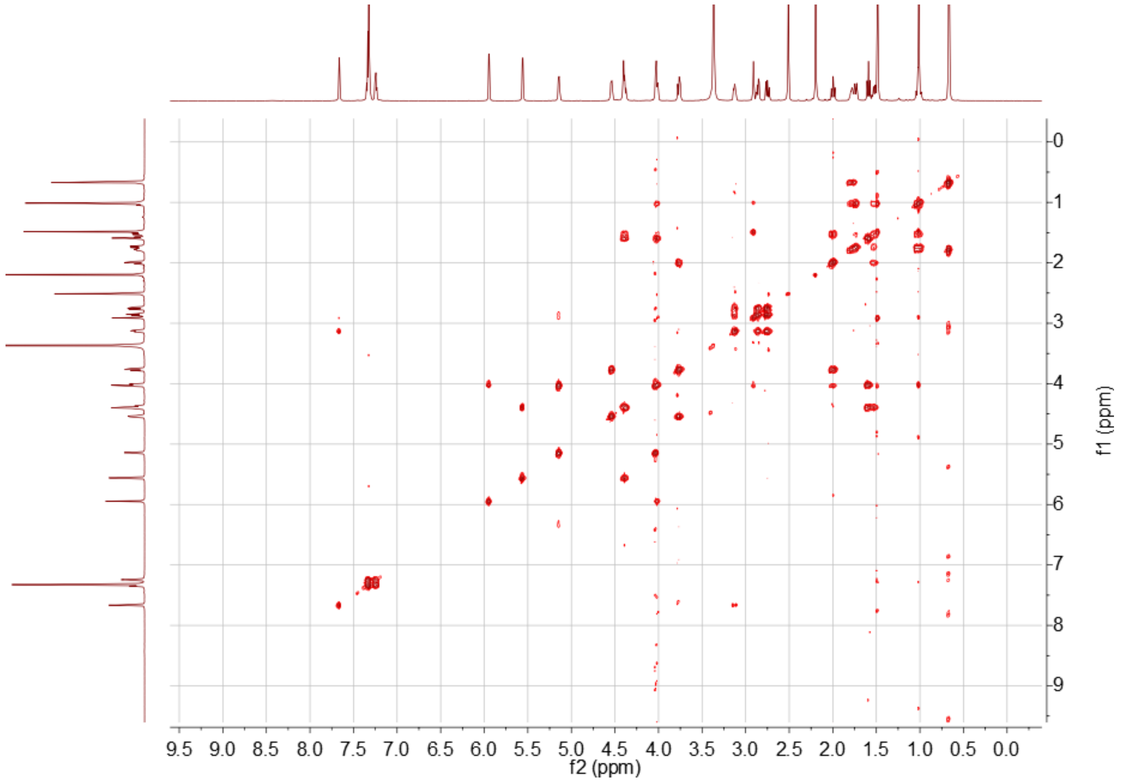

Figure S1.6 ROESY spectrum of **1** in DMSO-d_6

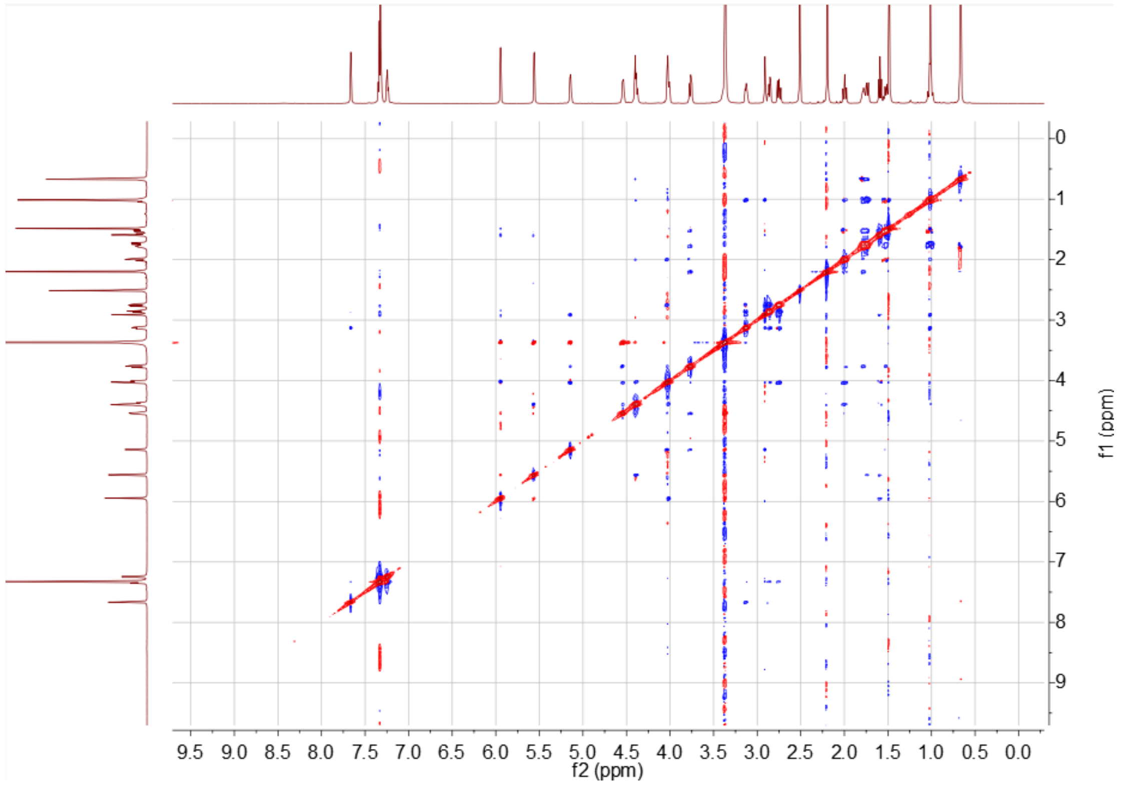

Figure S1.7 HRMS spectrum of **1**

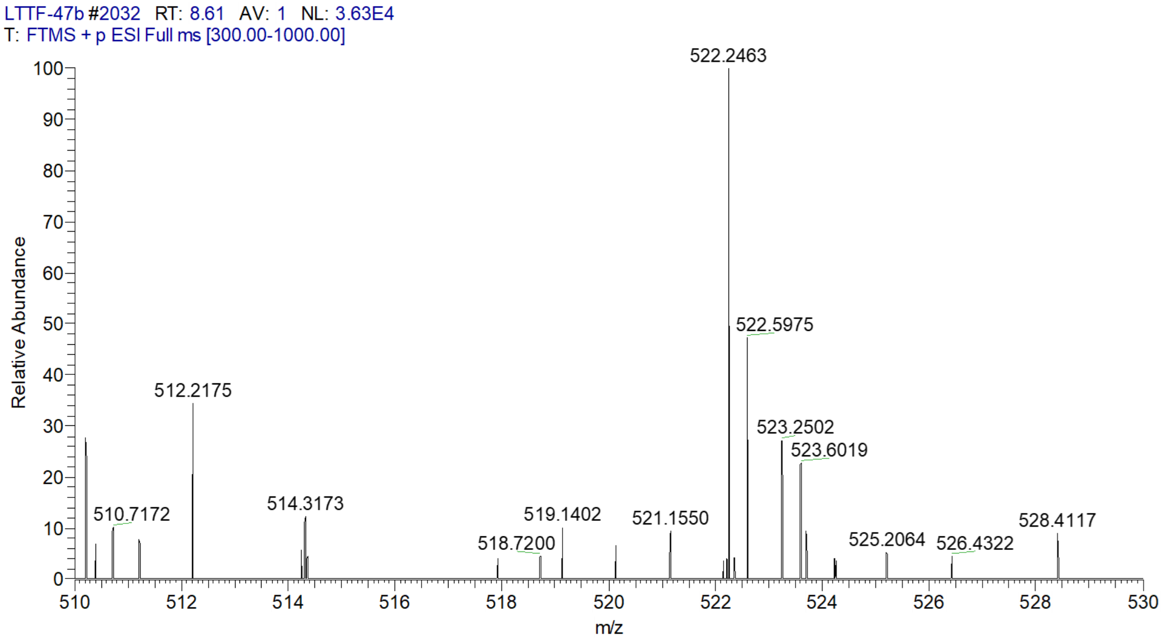

Figure S2.1 ^{1}H NMR spectrum of **2** in DMSO-d_6

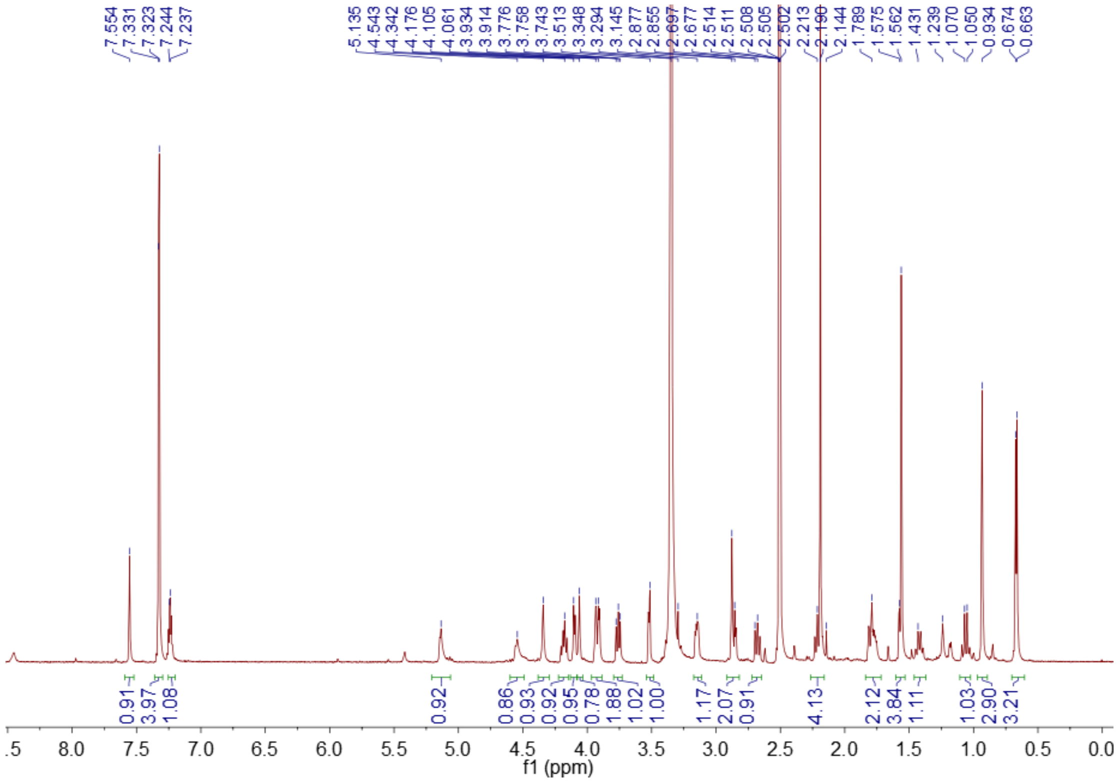

Figure S2.2 ^{13}C NMR spectrum of **2** in DMSO-d_6

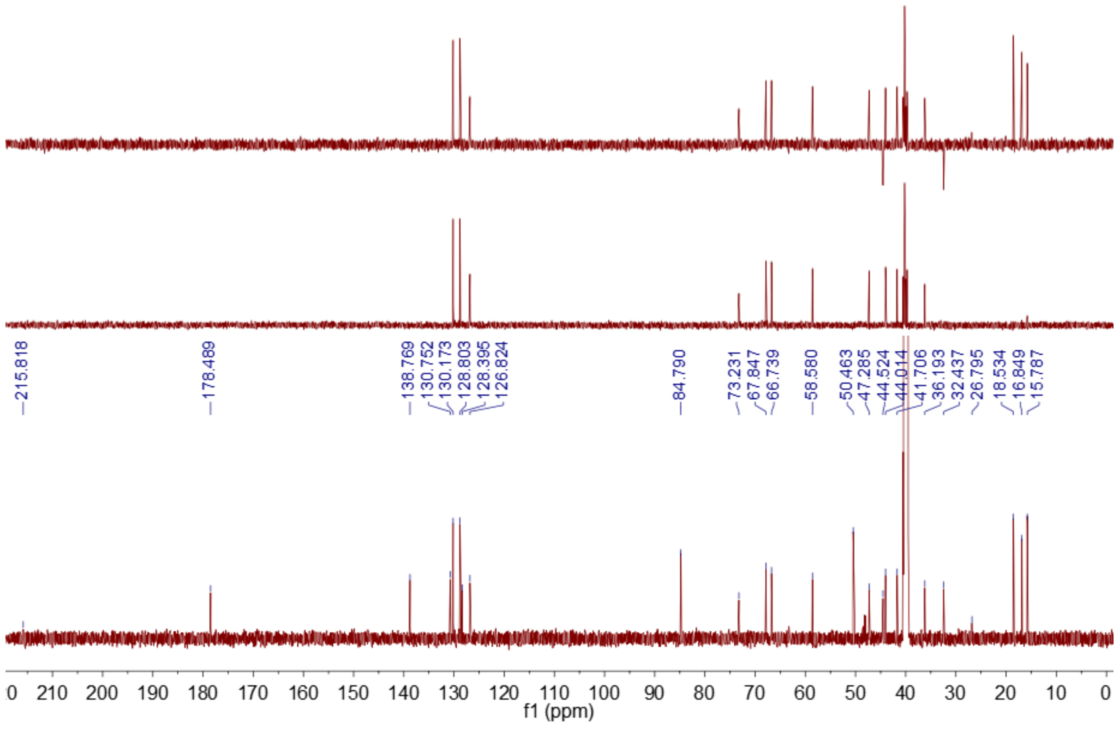

Figure S2.3 HSQC spectrum of **2** in DMSO-d_6

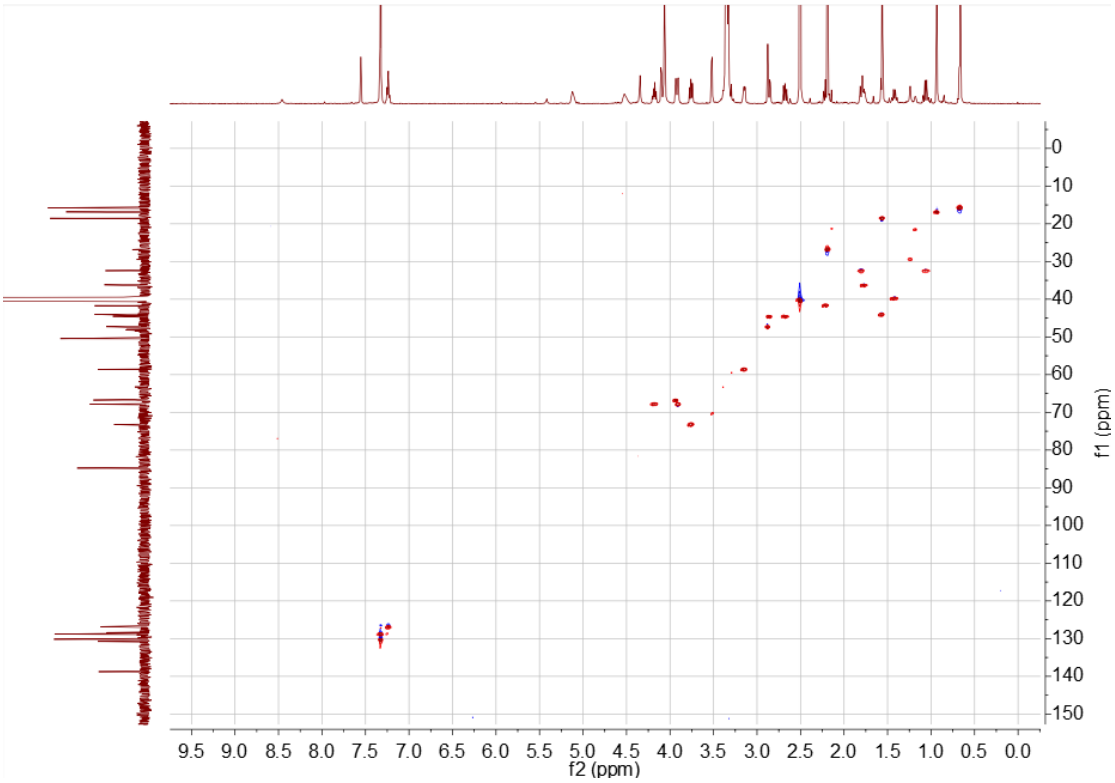

Figure S2.4 HMBC spectrum of **2** in DMSO-d_6

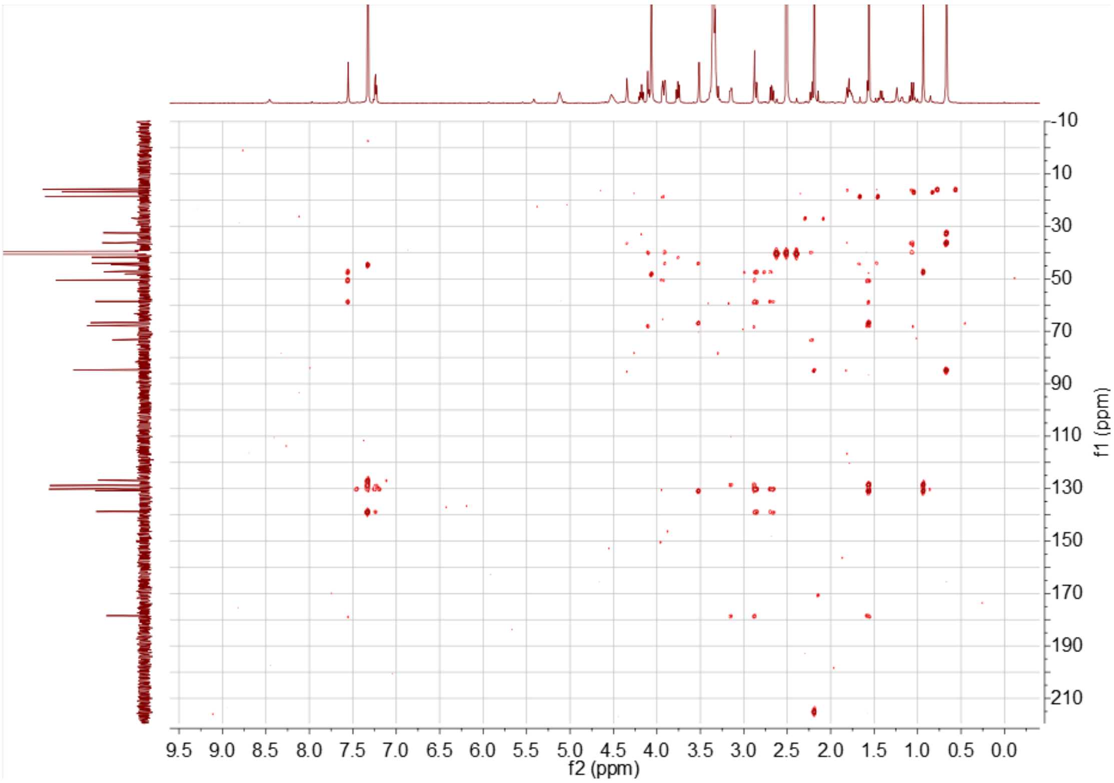

Figure S2.5 COSY spectrum of **2** in DMSO-d_6

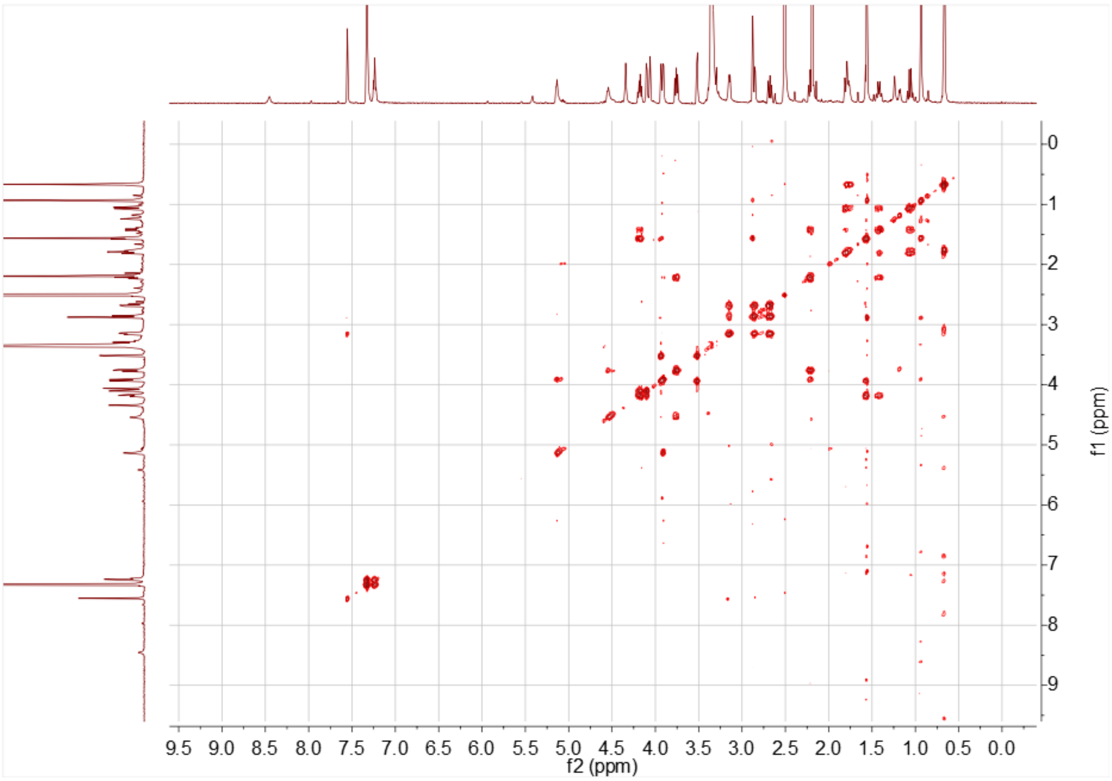

Figure S2.6 Roesy spectrum of **2** in DMSO-d_6

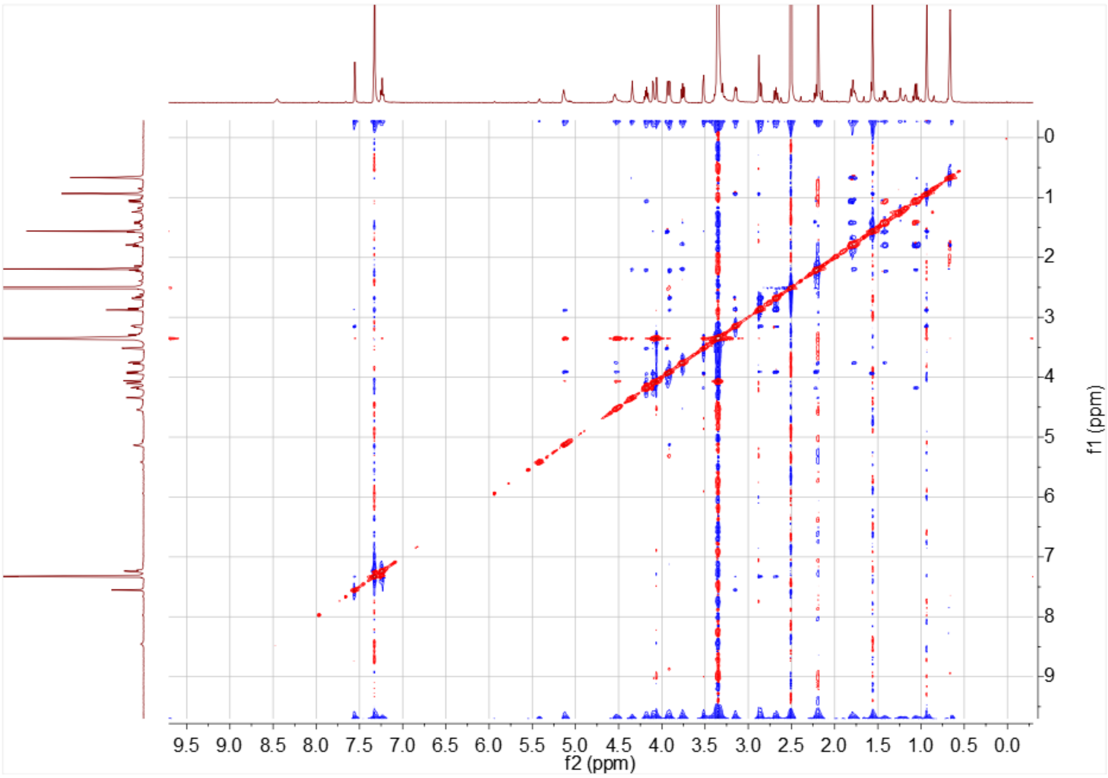

Figure S2.7 HRMS spectrum of **2**

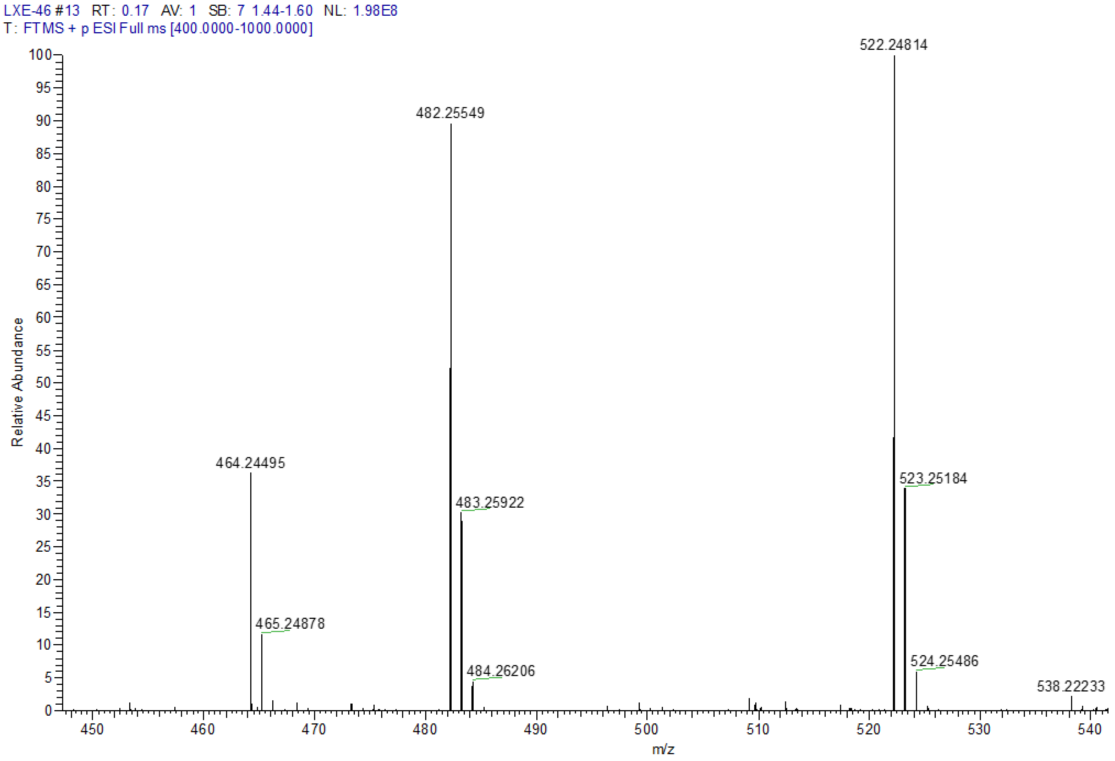

Figure S3.1 ^{1}H NMR spectrum of **3** in CD_3OD

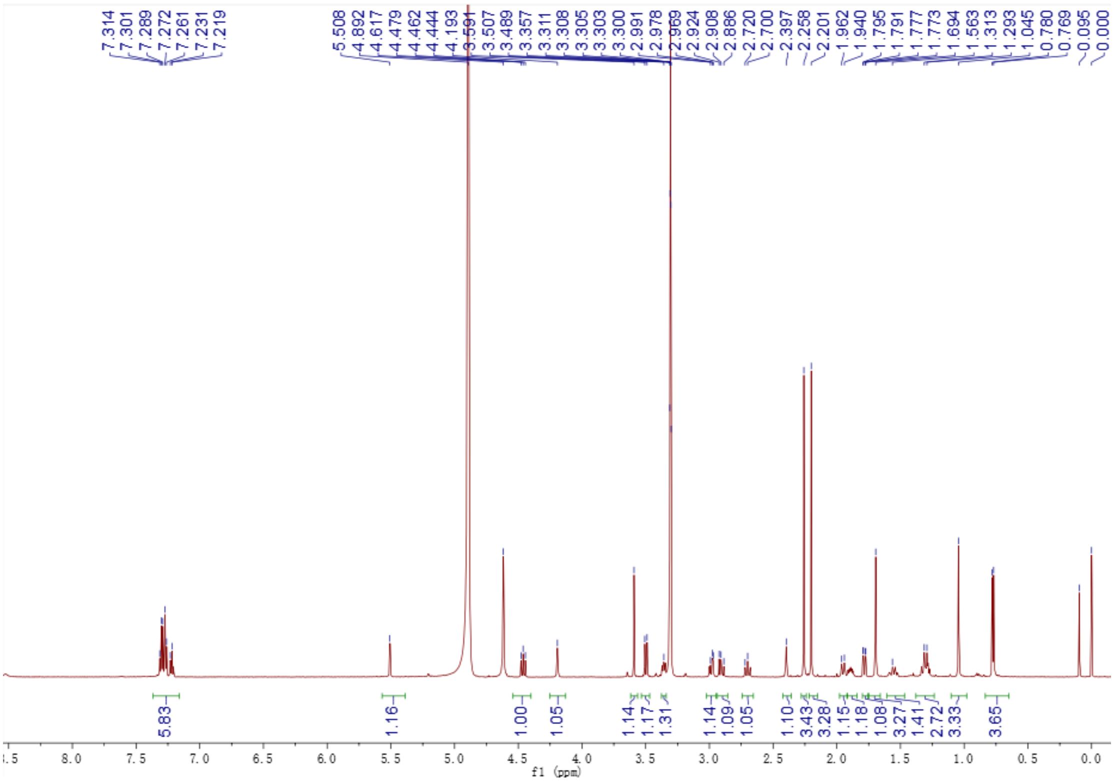

Figure S3.2 ^{13}C NMR spectrum of **3** in CD_3OD

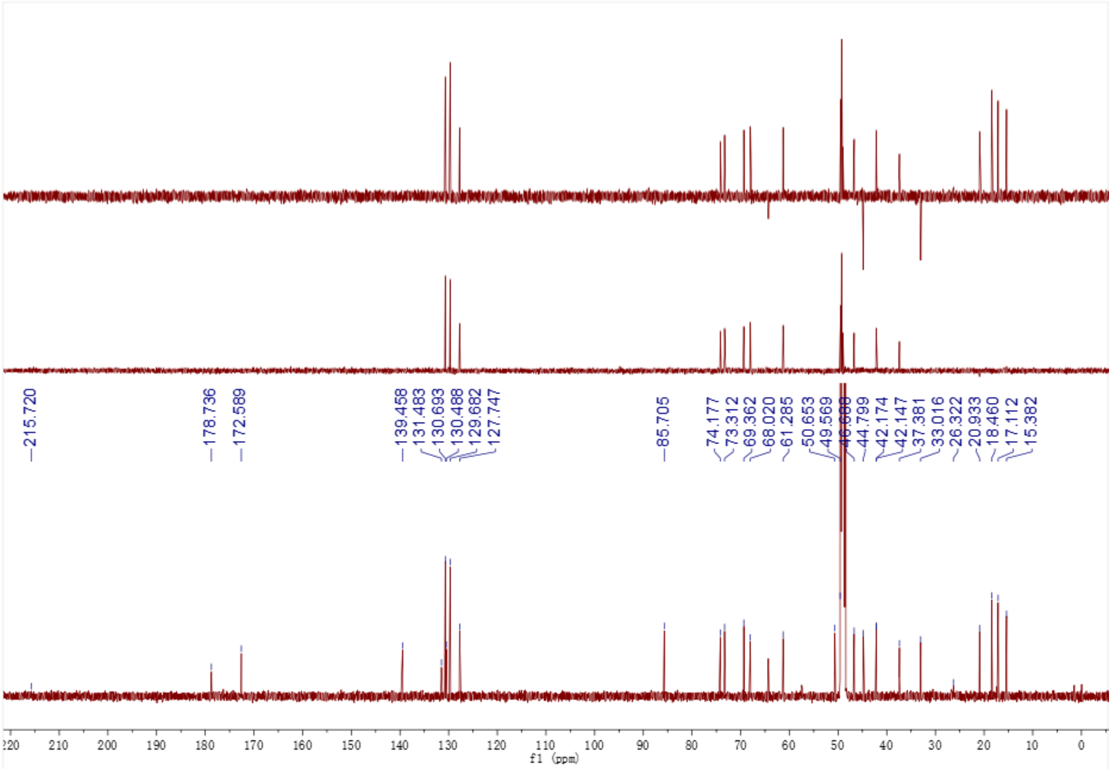

Figure S3.3 HSQC spectrum of **3** in CD_3OD

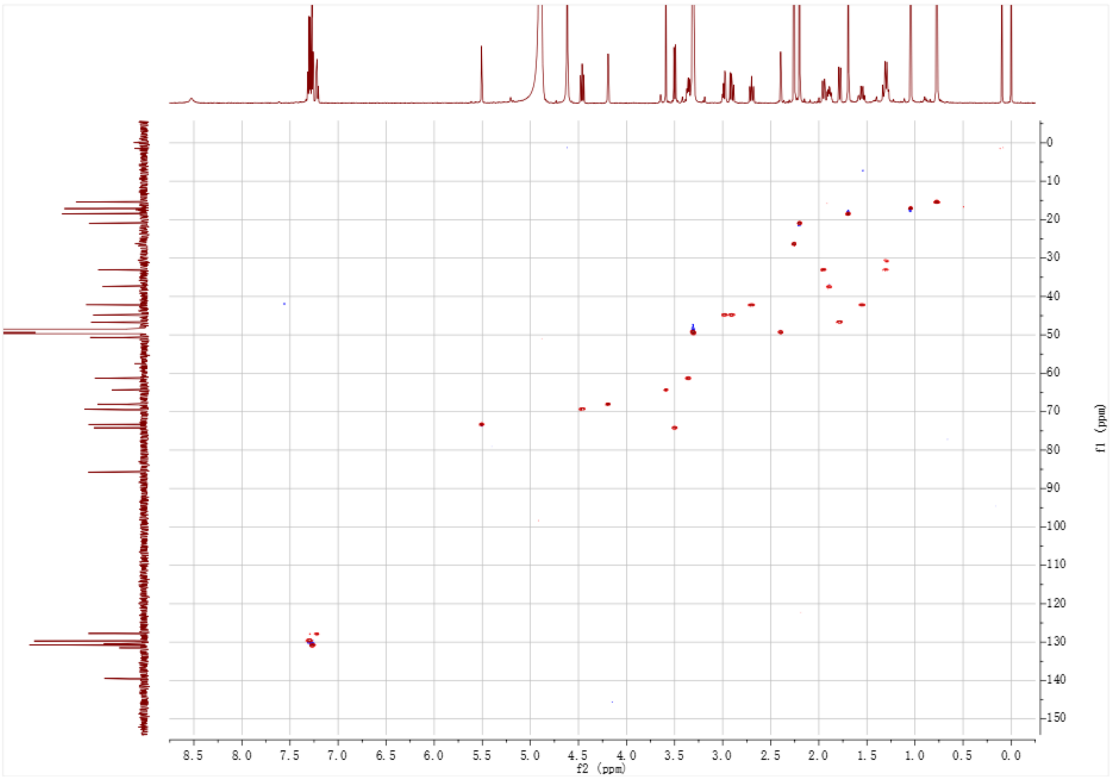

Figure S3.4 HMBC spectrum of **3** in CD_3OD

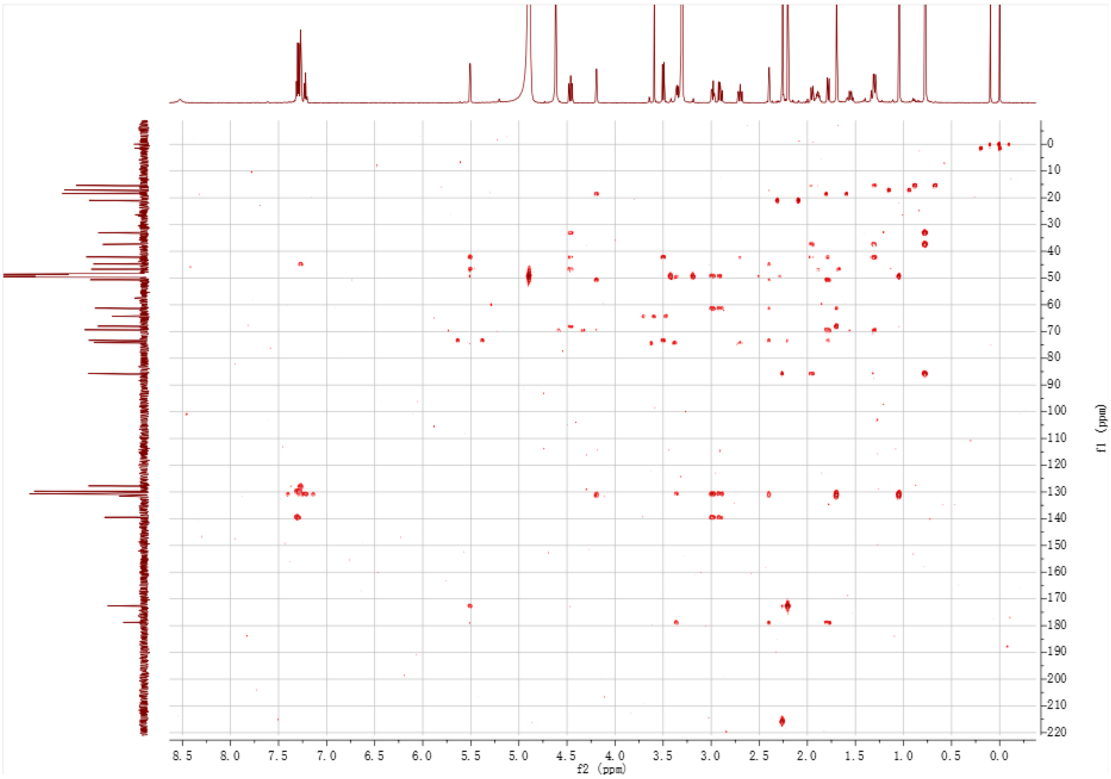

Figure S3.5 COSY spectrum of **3** in CD_3OD

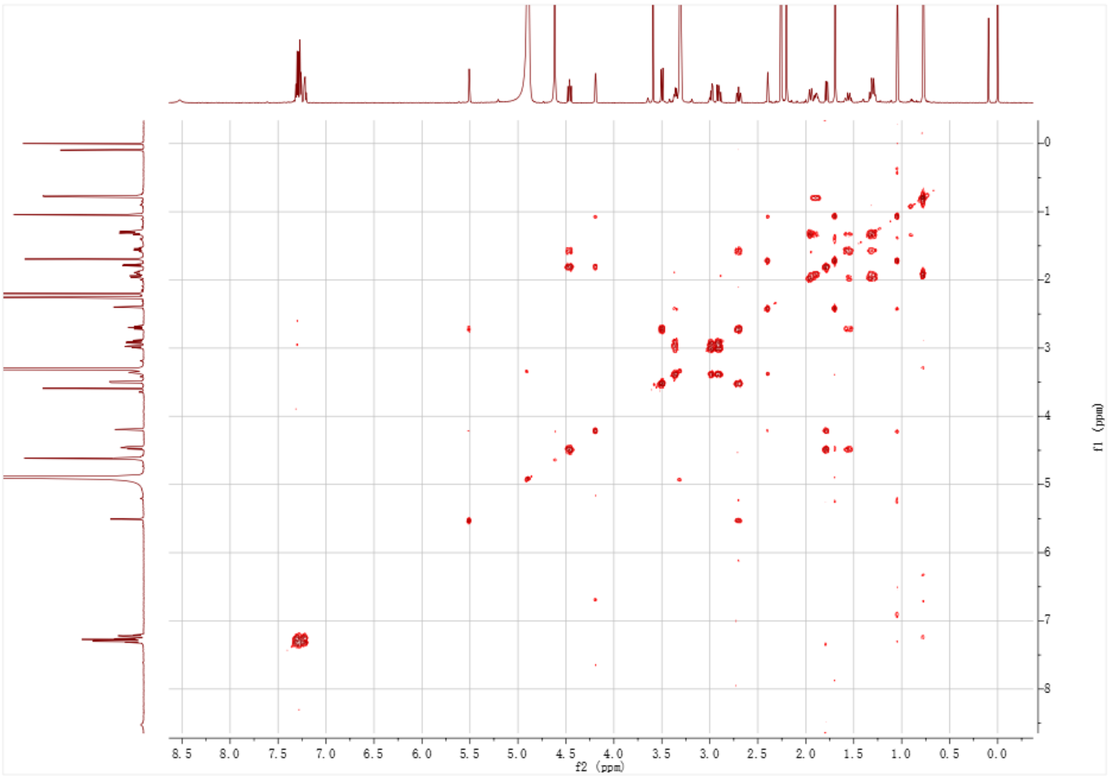

Figure S3.6 Roesy spectrum of **3** in CD_3OD

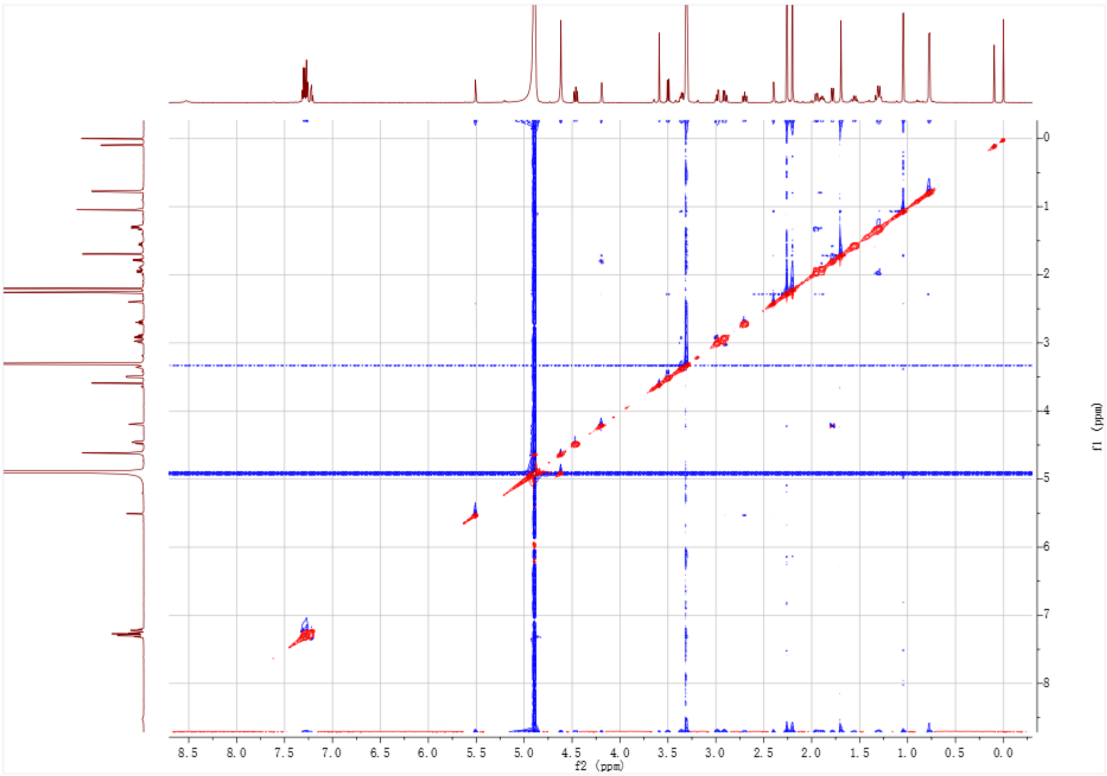

Figure S3.7 HRMS spectrum of **3**

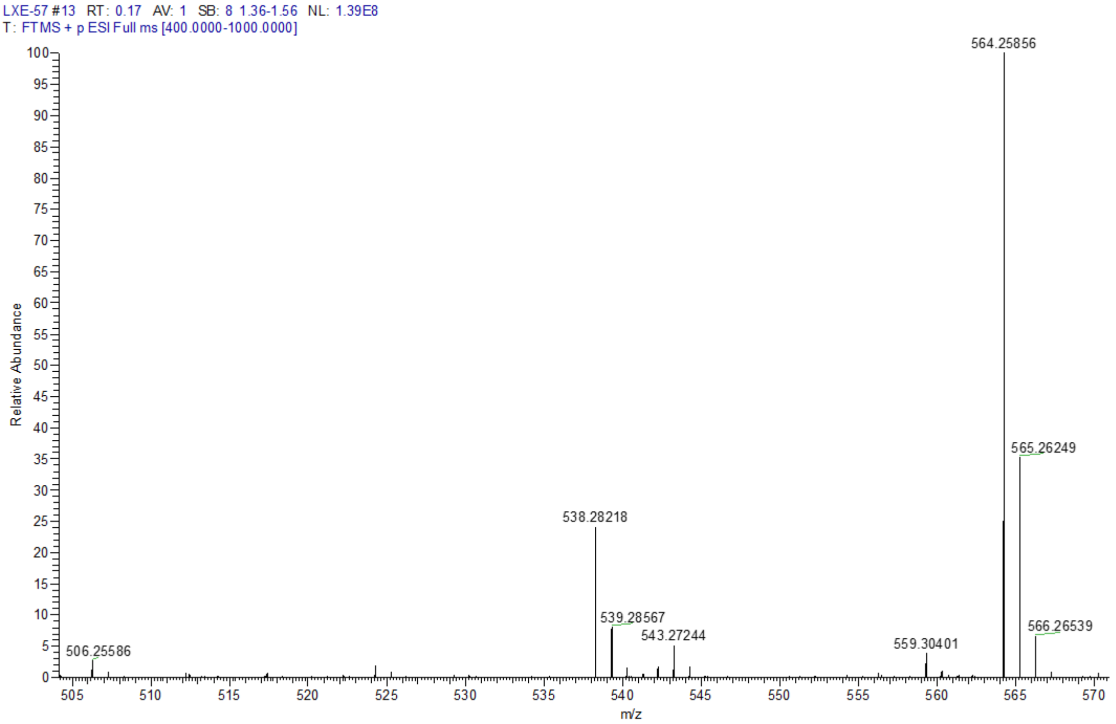

Figure S4.1 ^{1}H NMR spectrum of **4** in CD_3OD

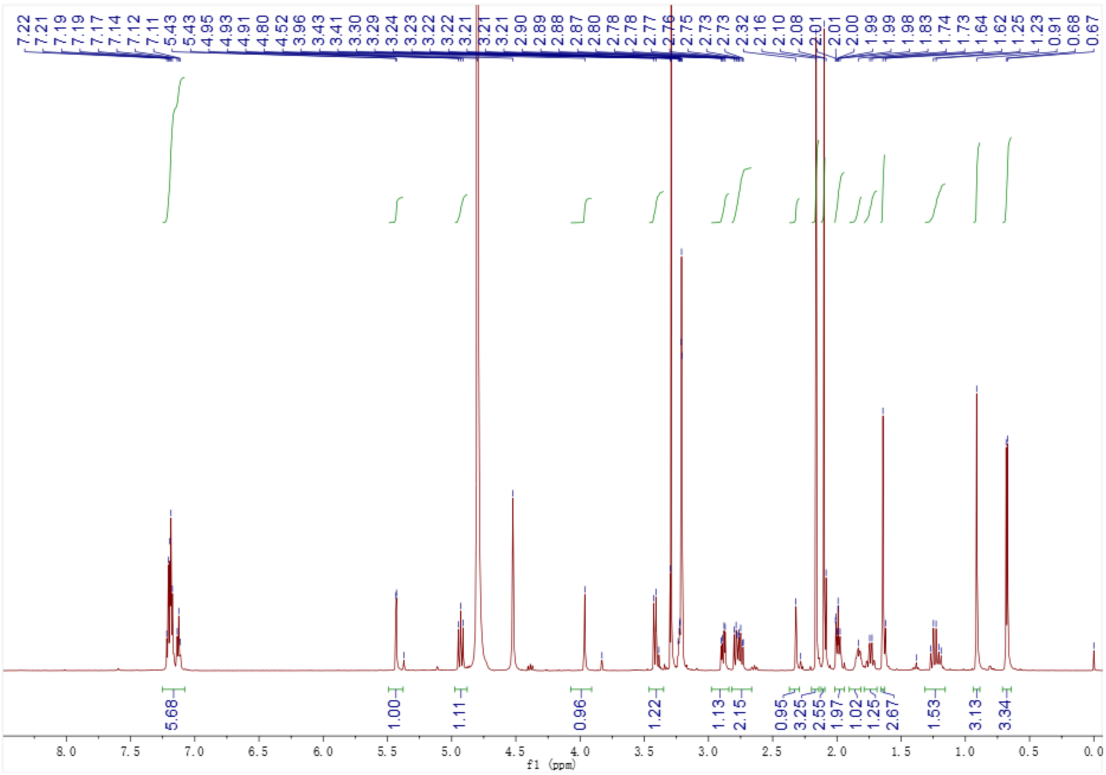

Figure S4.2 ^{13}C NMR spectrum of **4** in CD_3OD

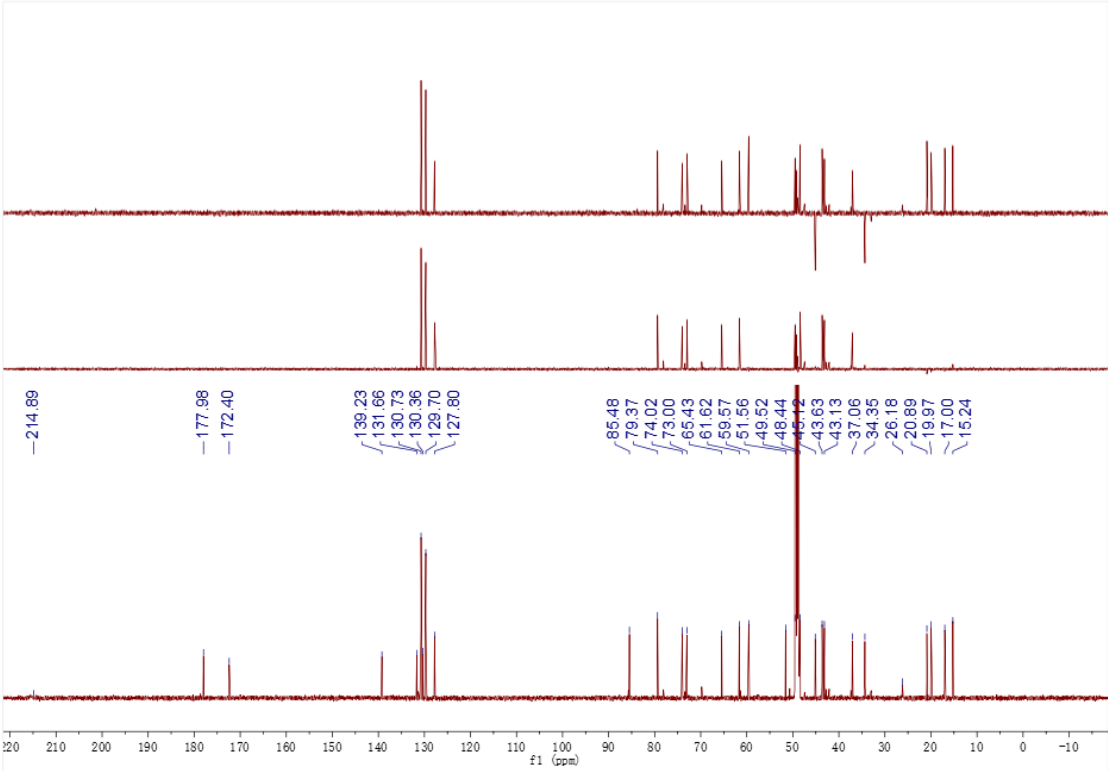

Figure S4.3 HSQC spectrum of **4** in CD_3OD

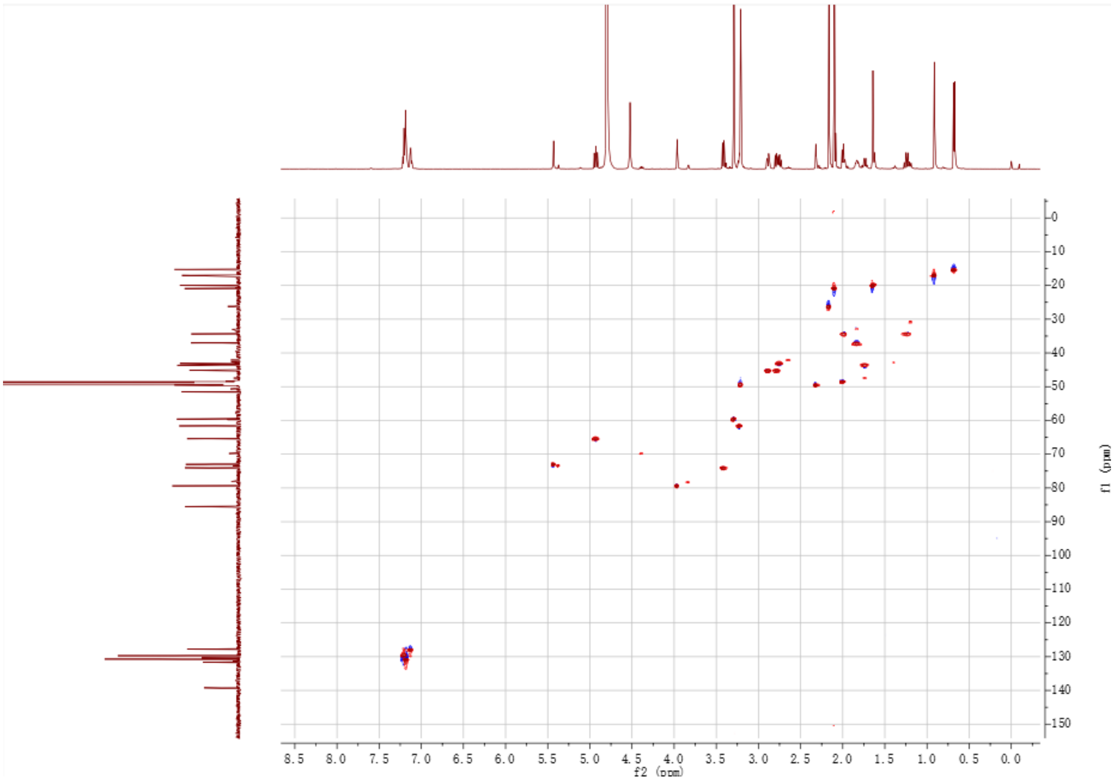

Figure S4.4 HMBC spectrum of **4** in CD_3OD

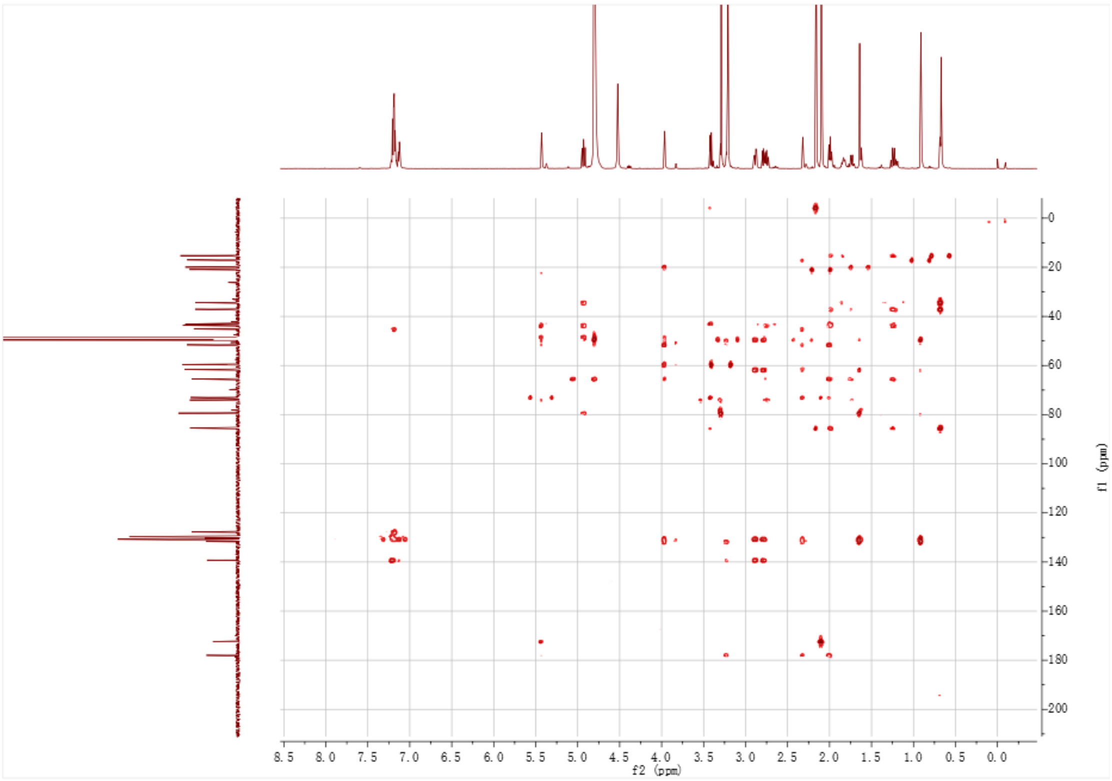

Figure S4.5 COSY spectrum of **4** in CD_3OD

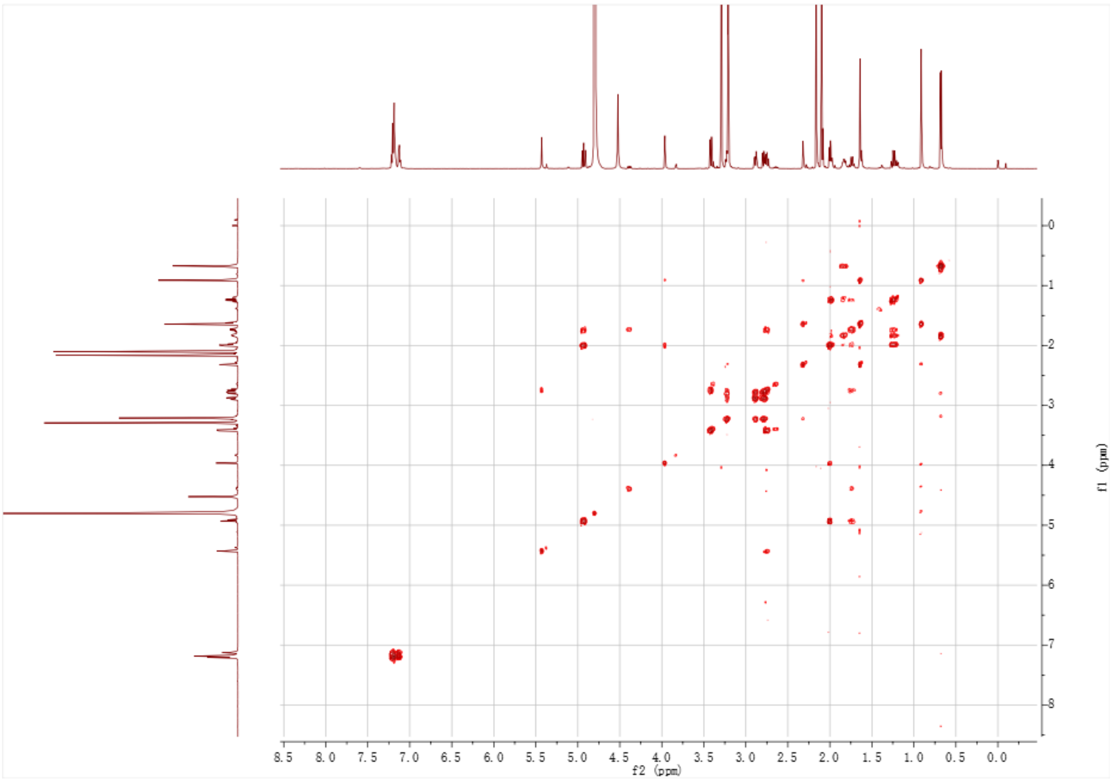

Figure S4.6 Roesy spectrum of **4** in CD_3OD

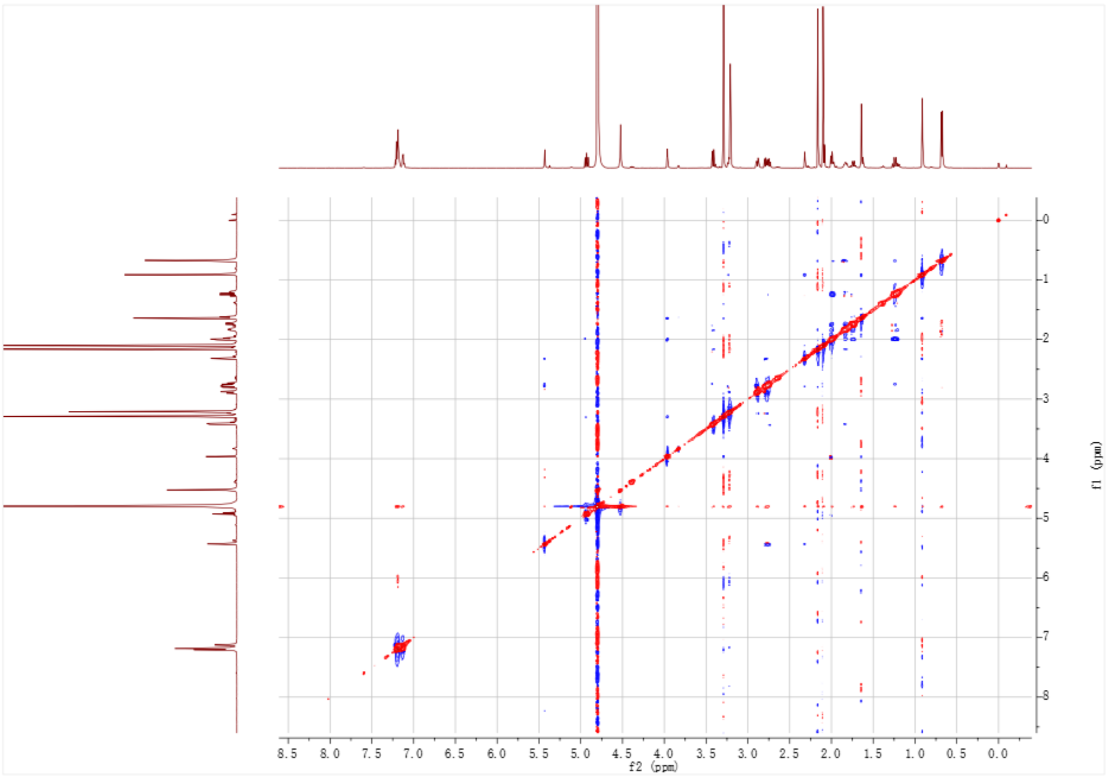

Figure S4.7 ^{1}H NMR spectrum of **4** in DMSO-d_6

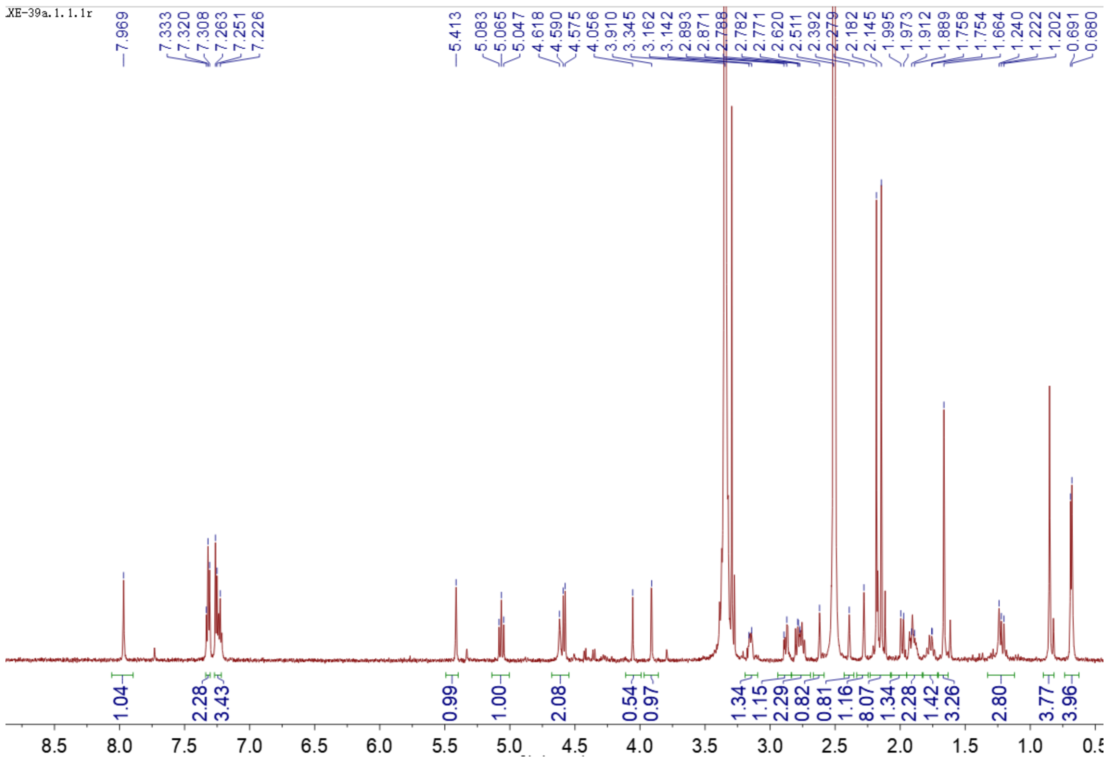

Figure S4.8 ^{13}C NMR spectrum of **4** in DMSO-d_6

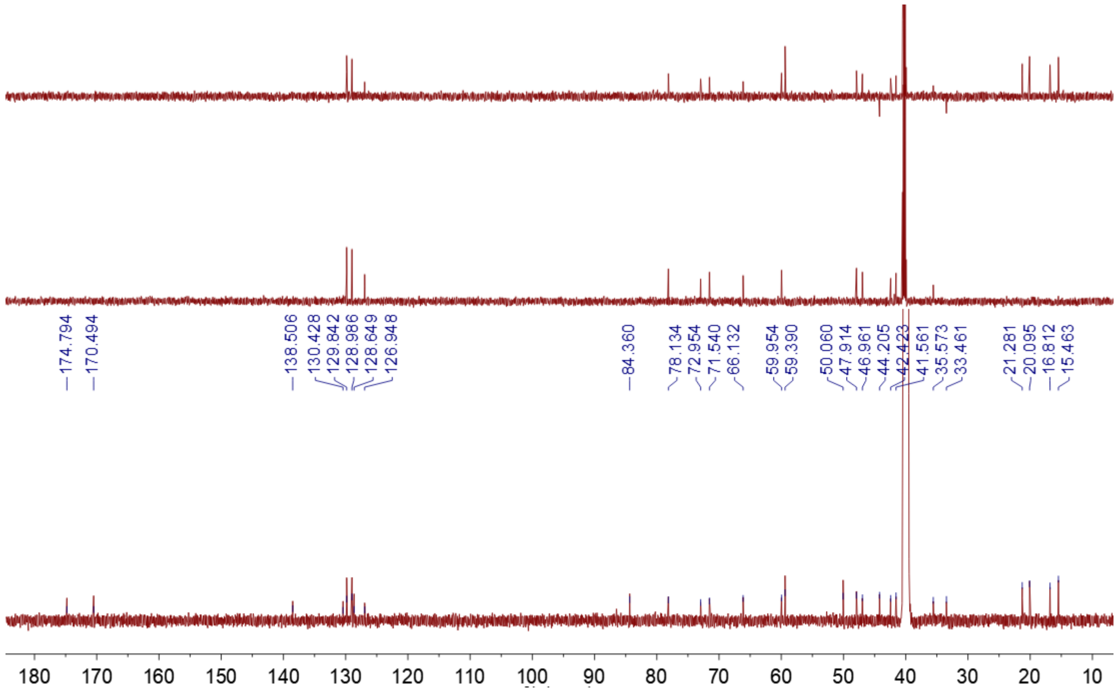

Figure S4.9 HSQC spectrum of **4** in DMSO-d_6

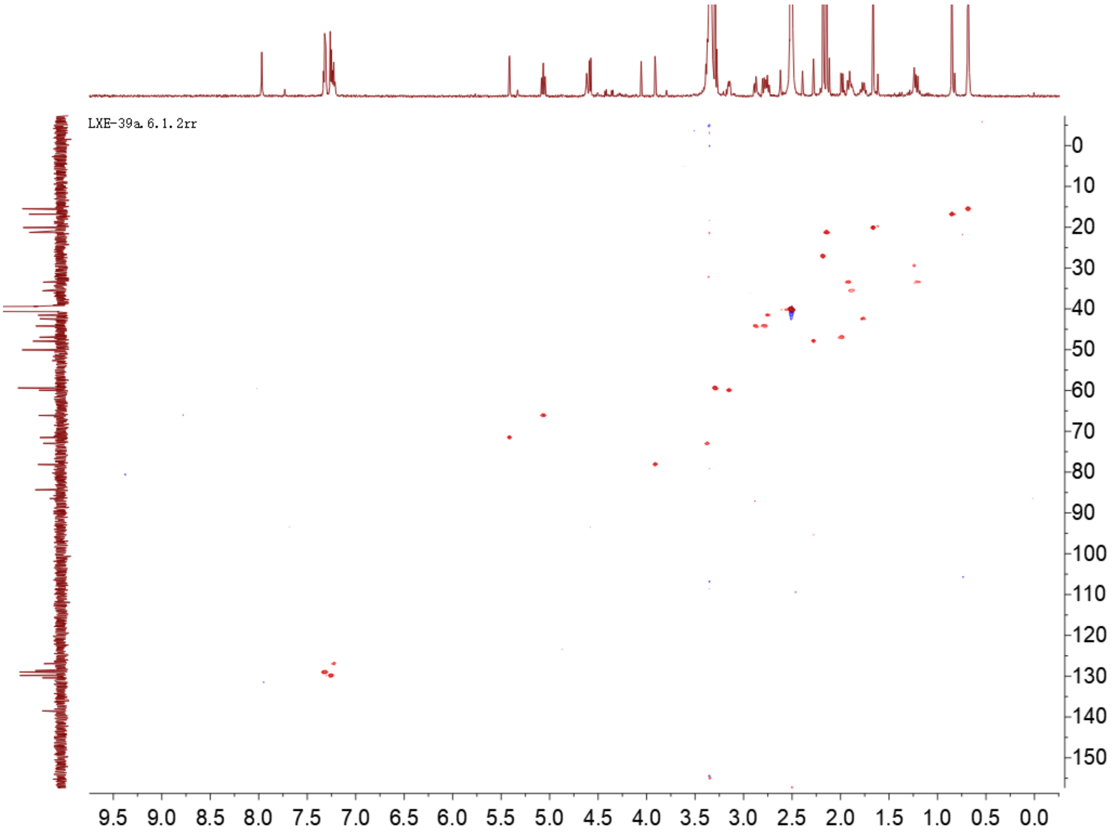

Figure S4.10 HMBC spectrum of **4** in DMSO-d_6

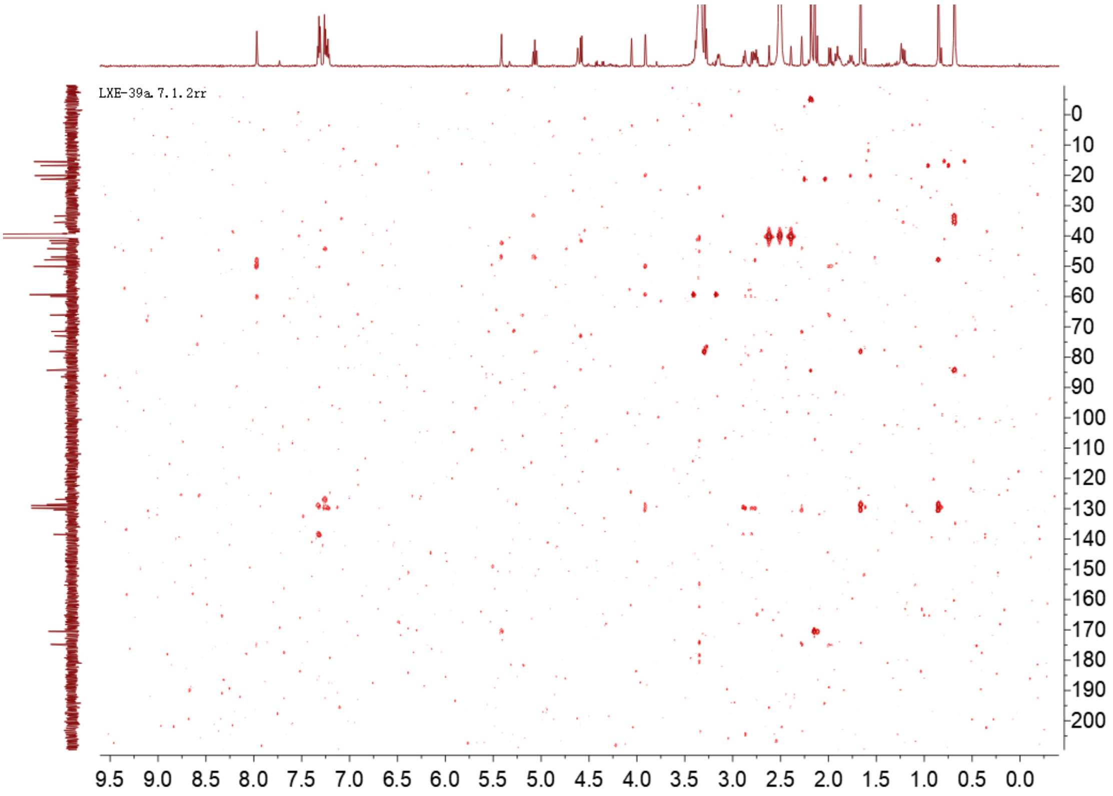

Figure S4.11 COSY spectrum of **4** in DMSO-d_6

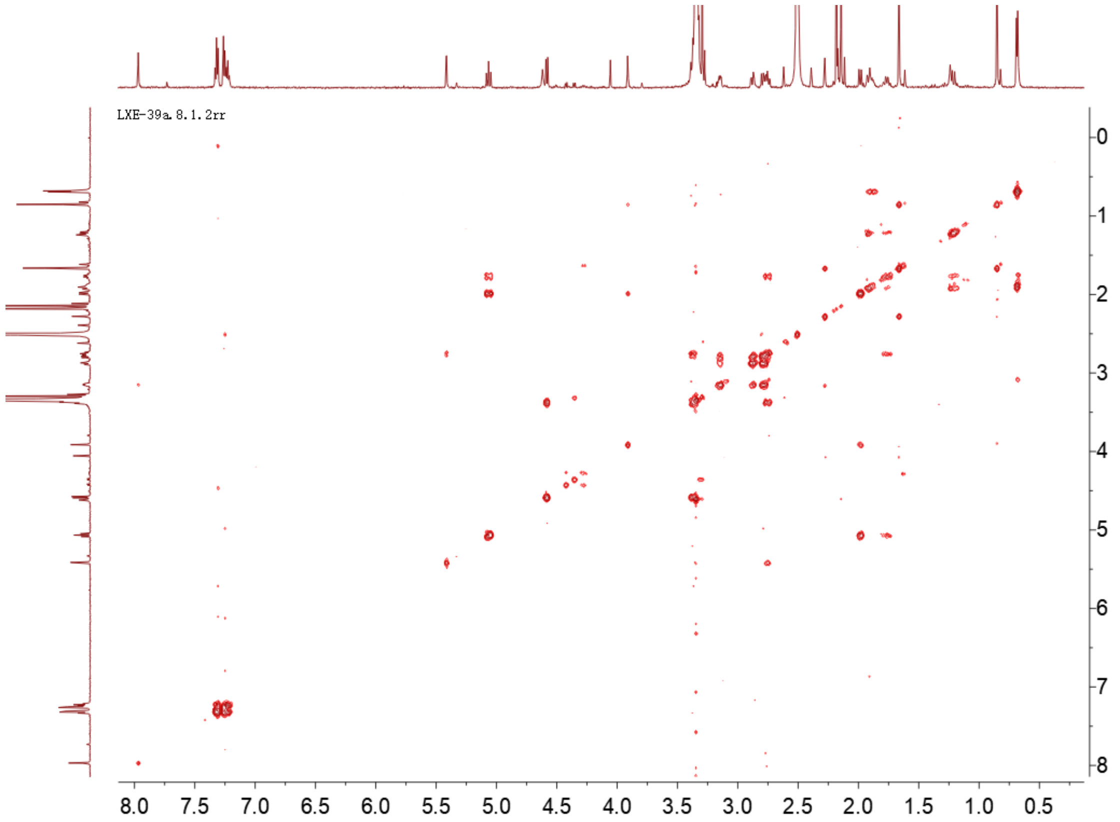

Figure S4.12 Roesy spectrum of **4** in DMSO-d_6

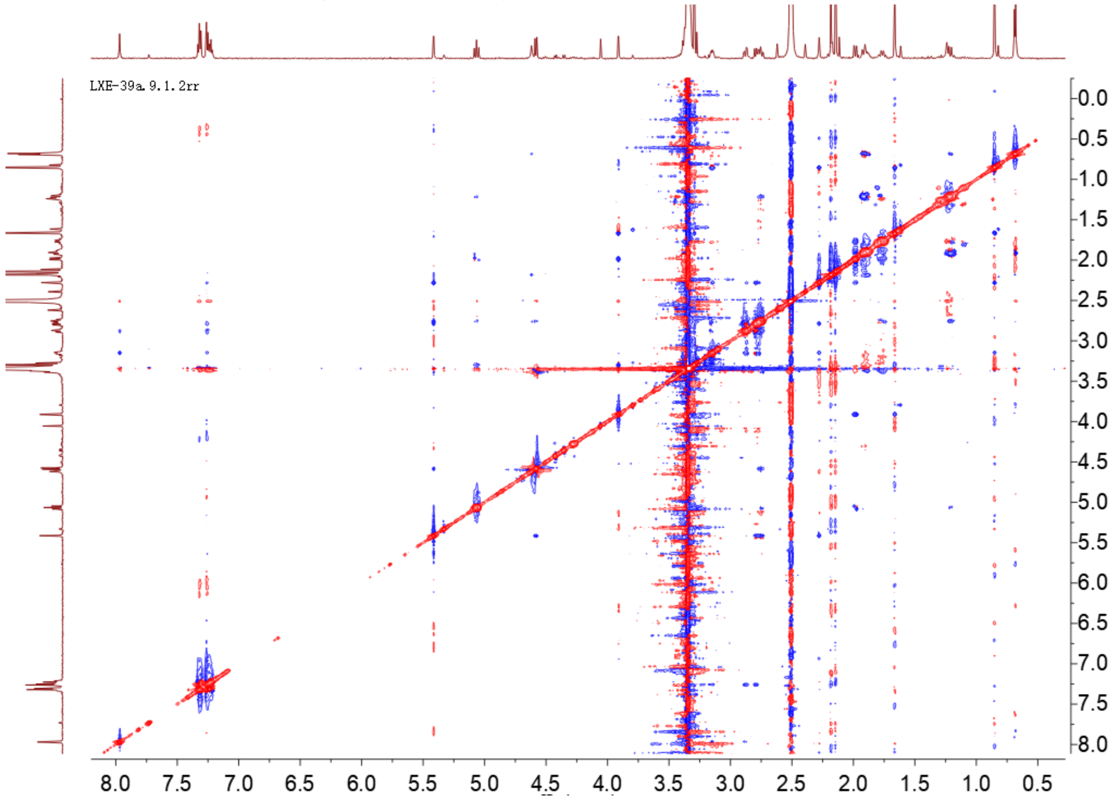

Figure S4.13 HRMS spectrum of **4**

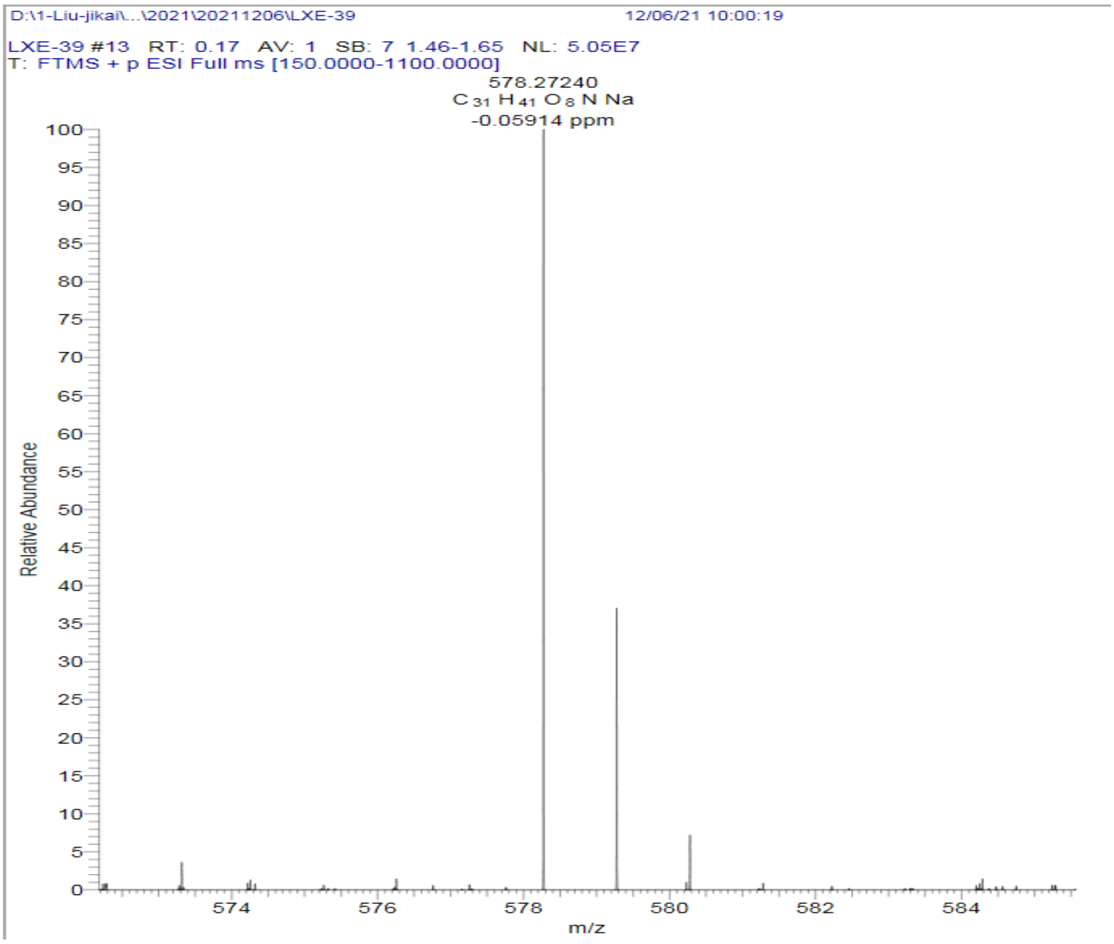

Figure S5.1 ^{1}H NMR spectrum of **5** in CD_3OD

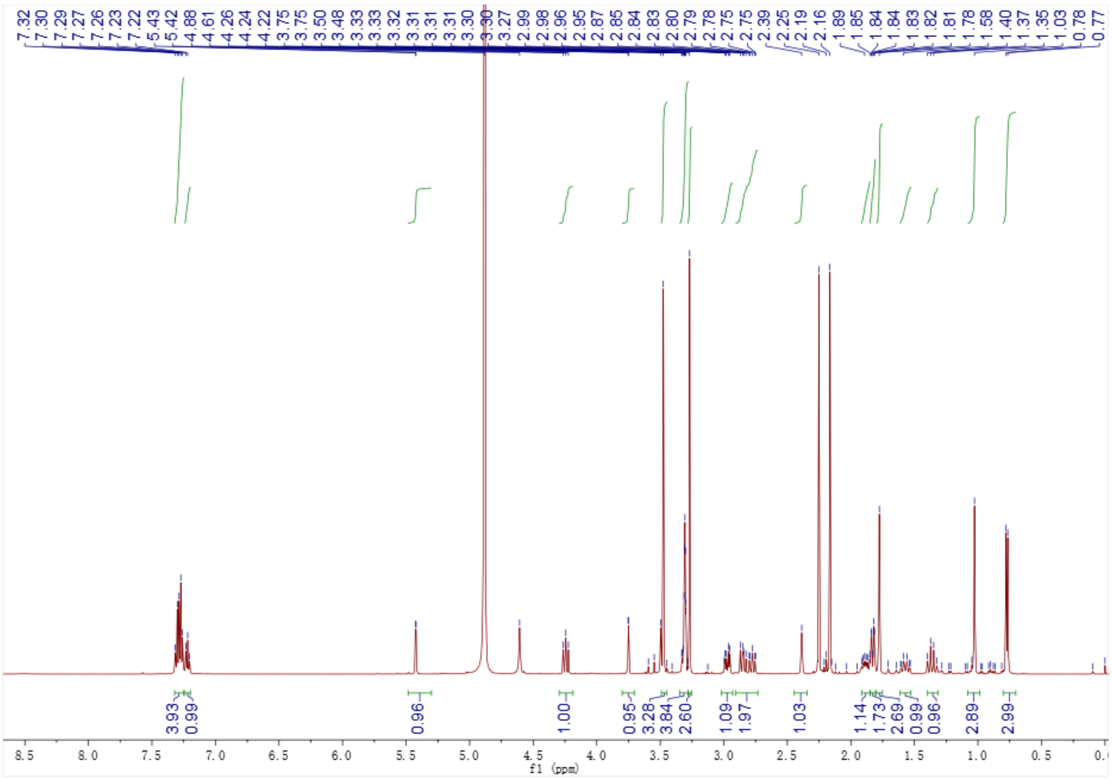

Figure S5.2 ^{13}C NMR spectrum of **5** in CD_3OD

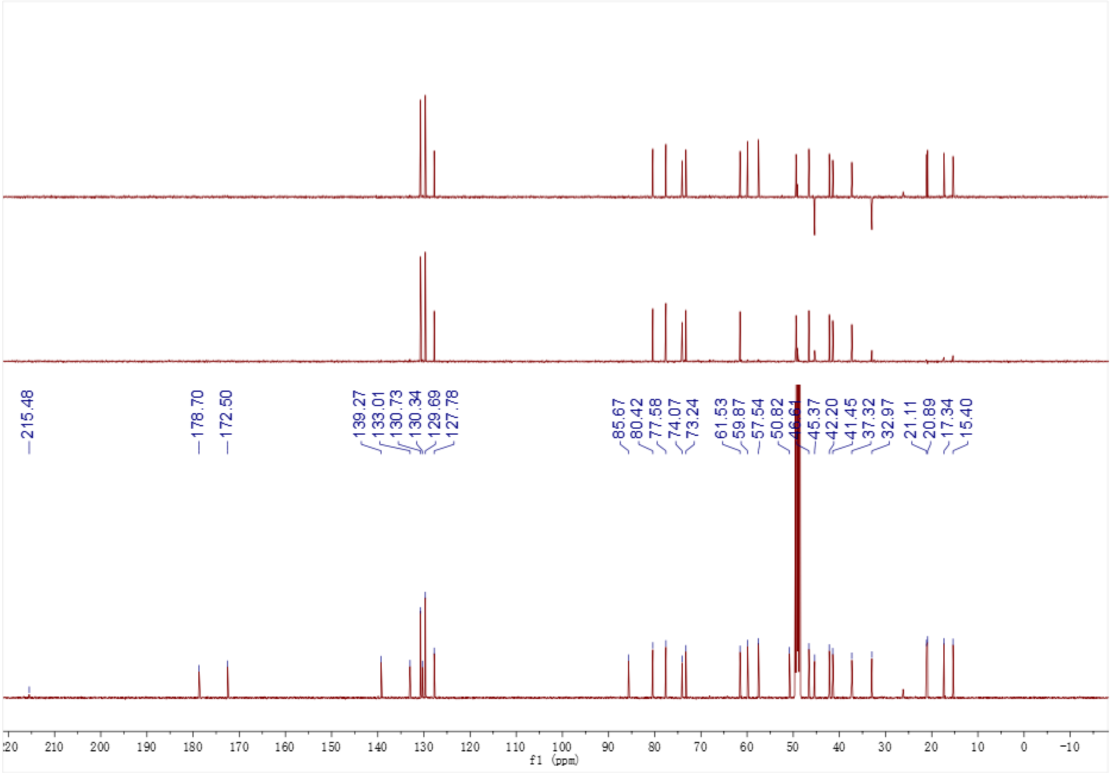

Figure S5.3 HSQC spectrum of **5** in CD_3OD

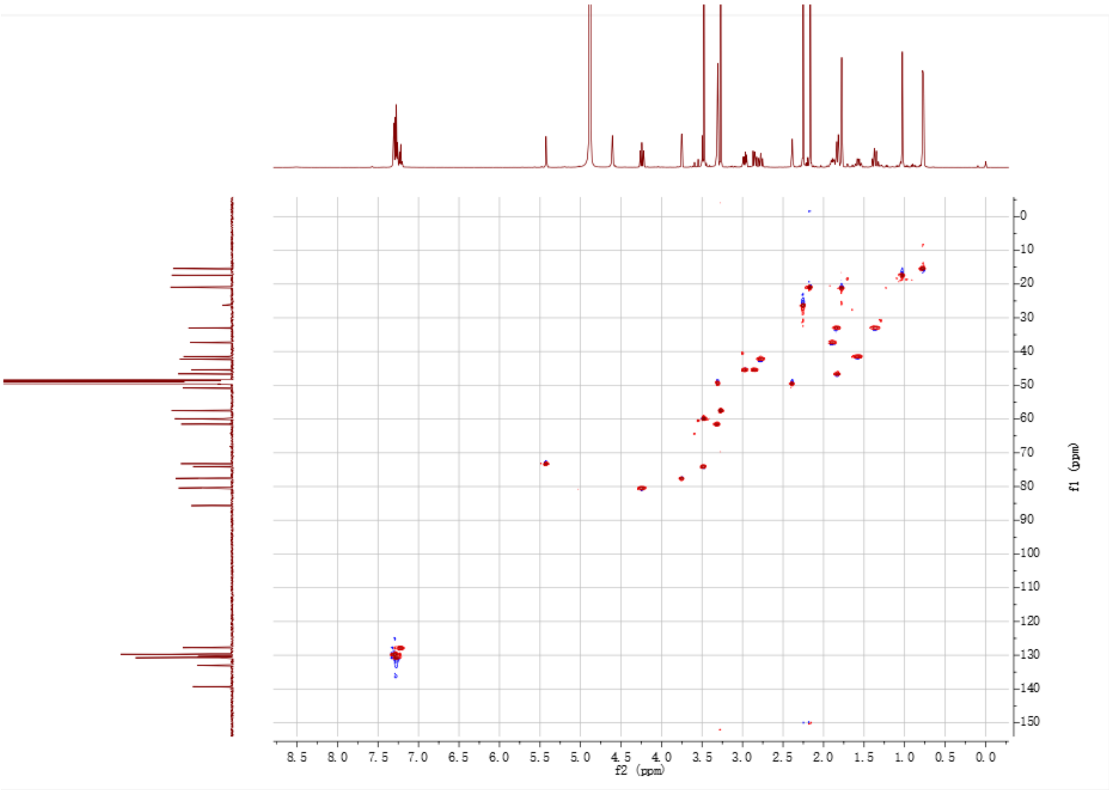

Figure S5.4 HMBC spectrum of **5** in CD_3OD

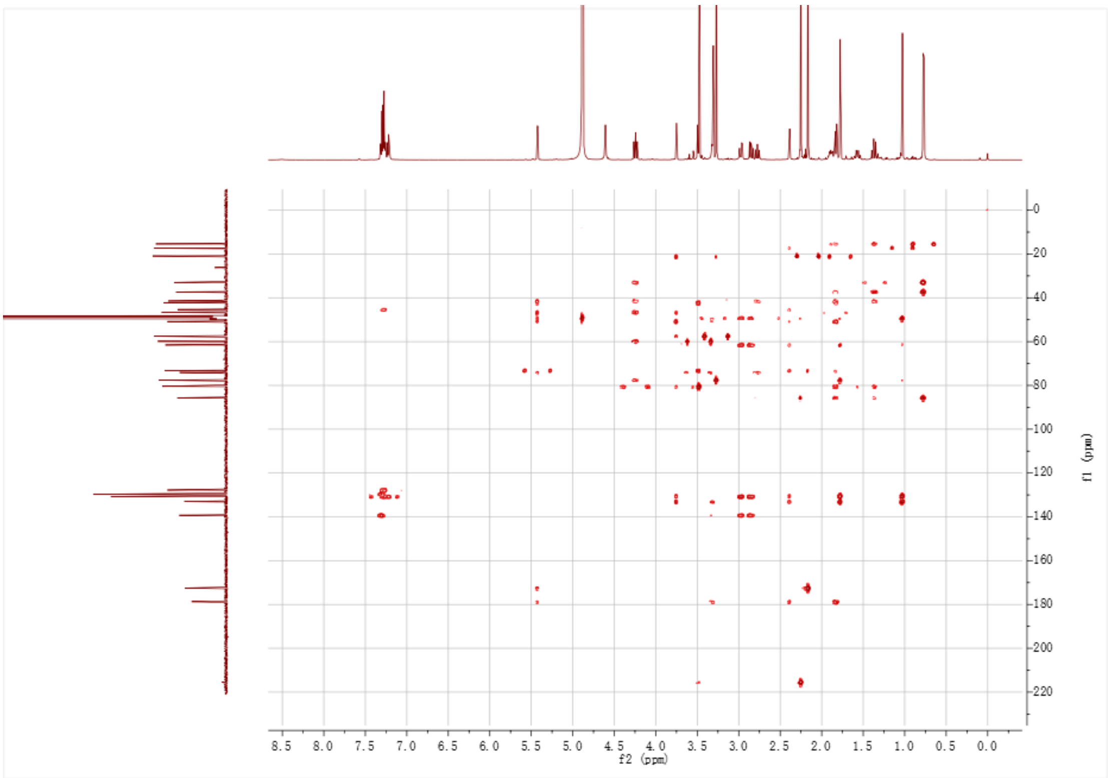

Figure S5.5 COSY spectrum of **5** in CD_3OD

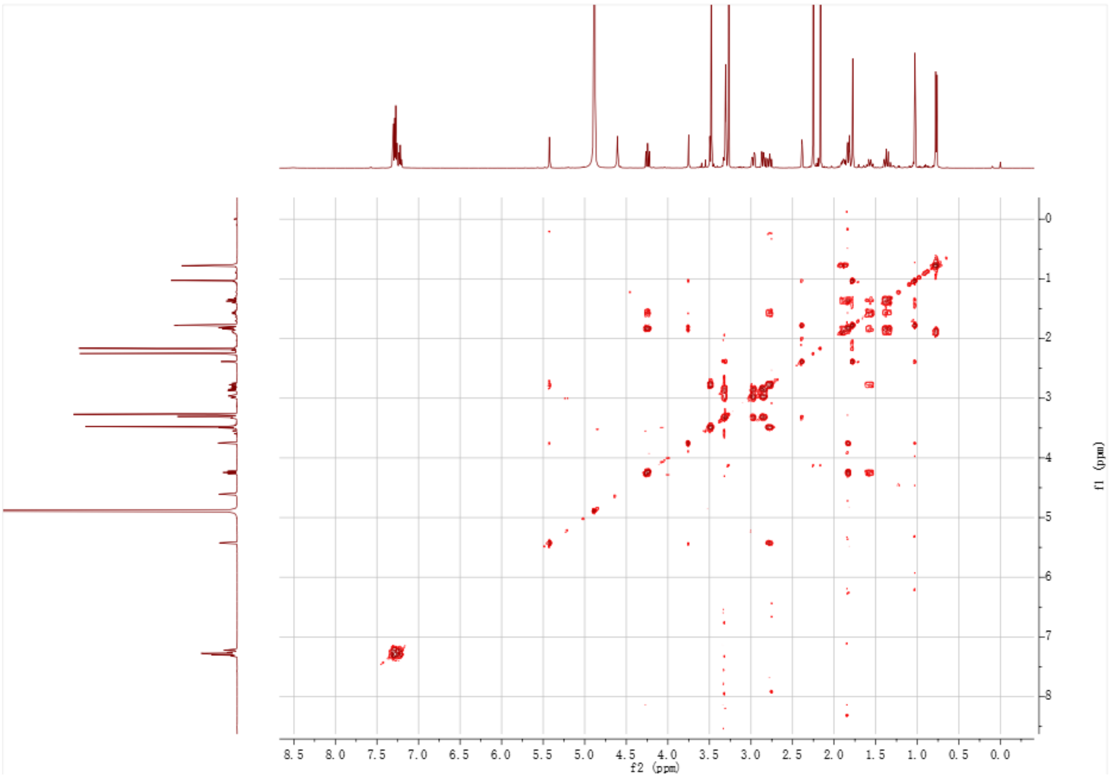

Figure S5.6 Roesy spectrum of **5** in CD_3OD

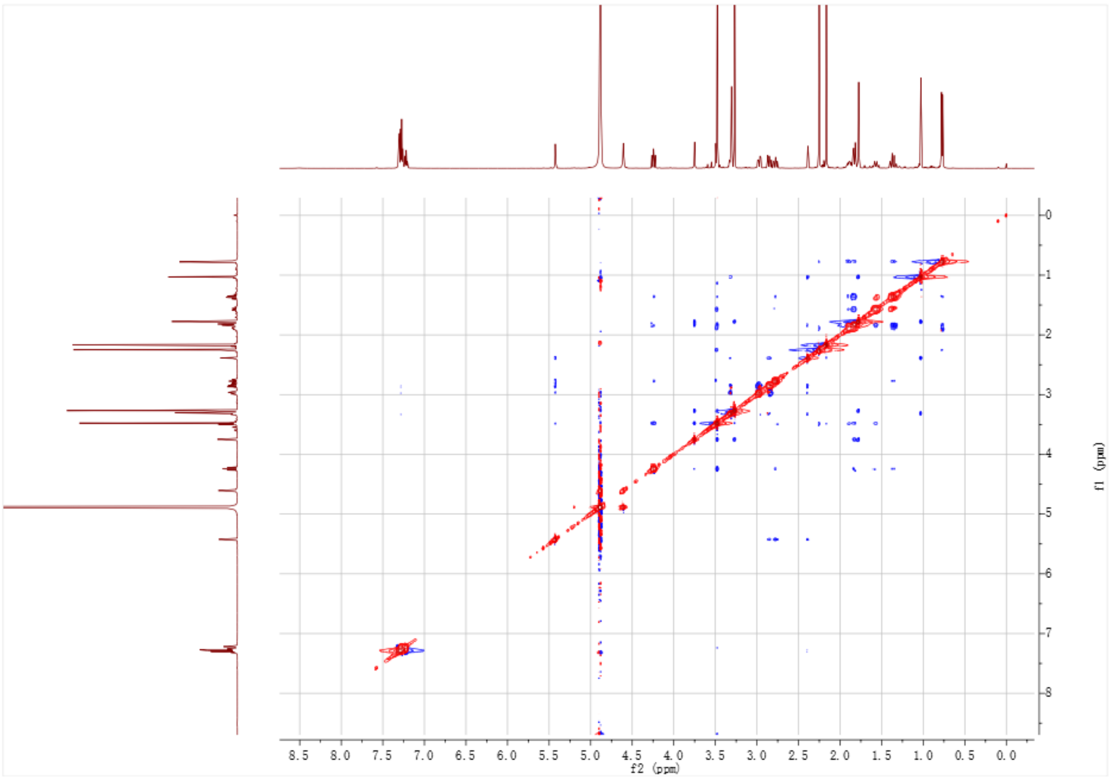

Figure S5.7 HRMS spectrum of **5**

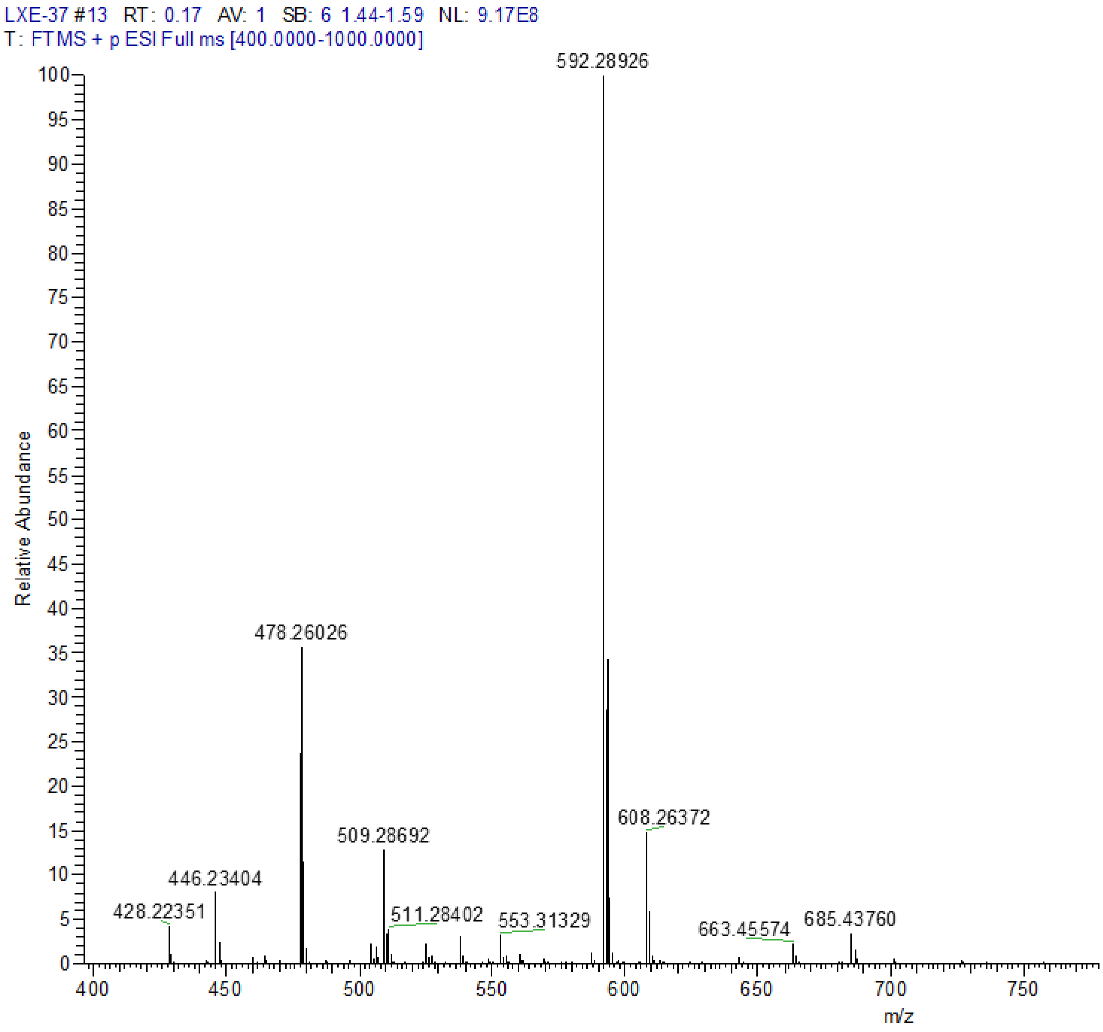

Figure S6.1 ^{1}H NMR spectrum of **6** in DMSO-d_6

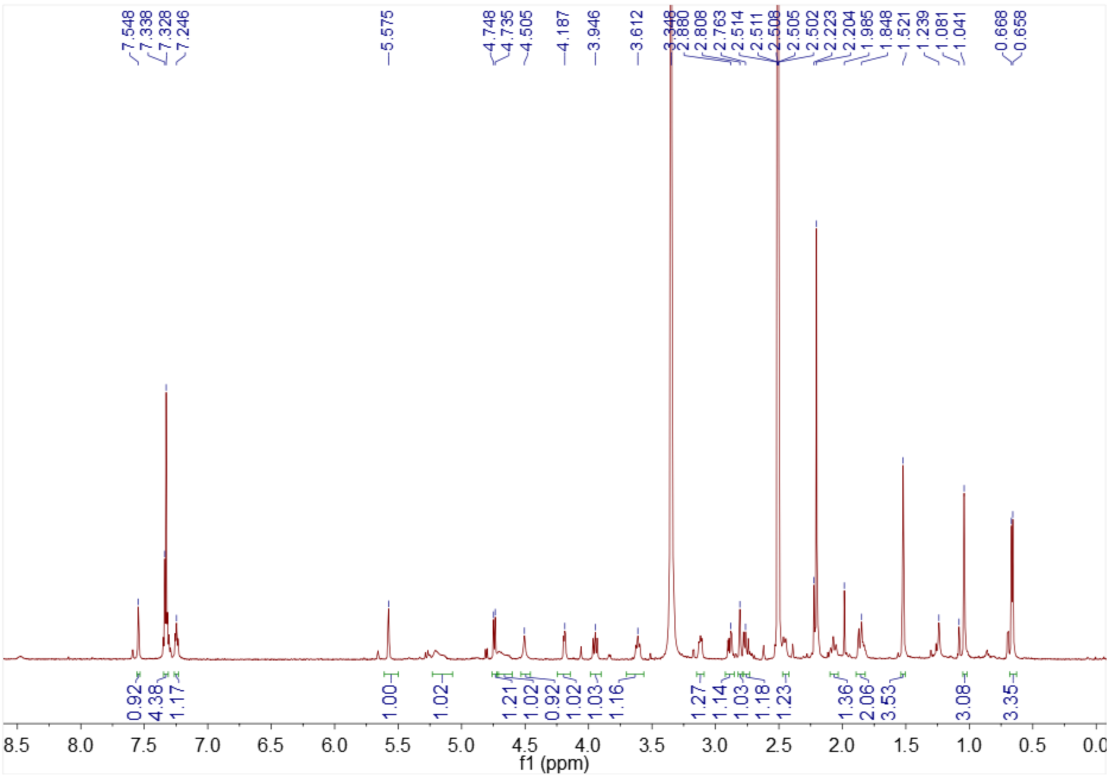

Figure S6.2 ^{13}C NMR spectrum of **6** in DMSO-d_6

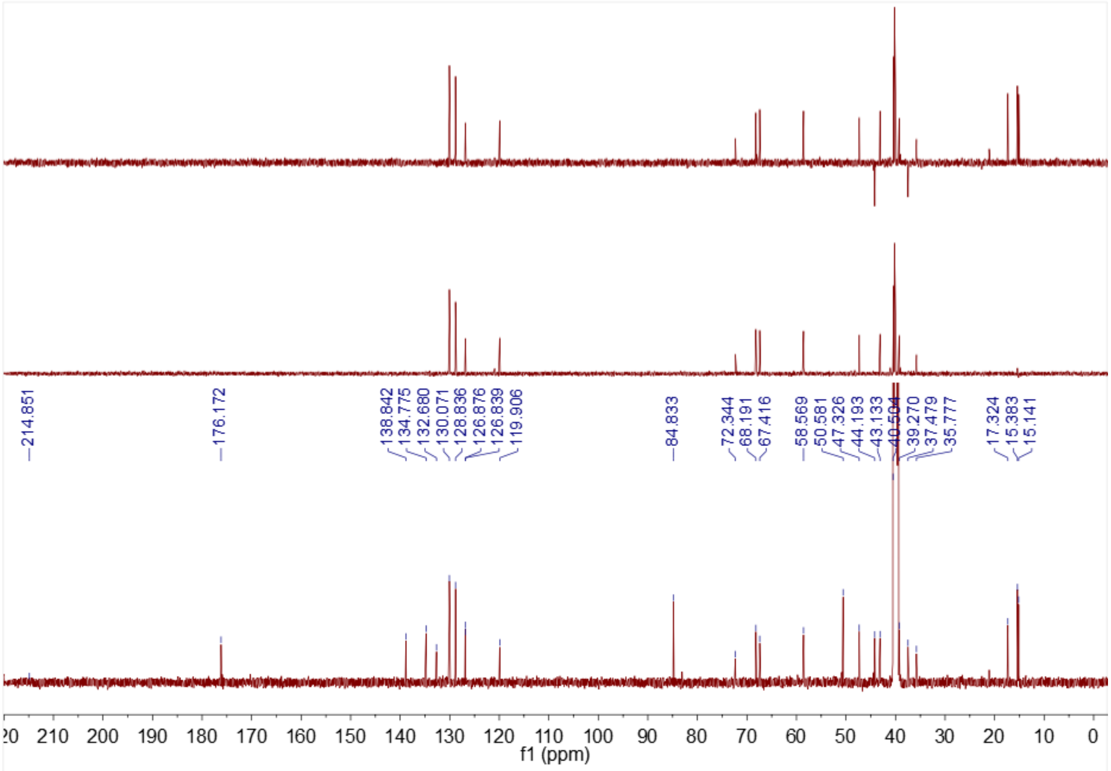

Figure S6.3 HSQC spectrum of **6** in DMSO-d_6

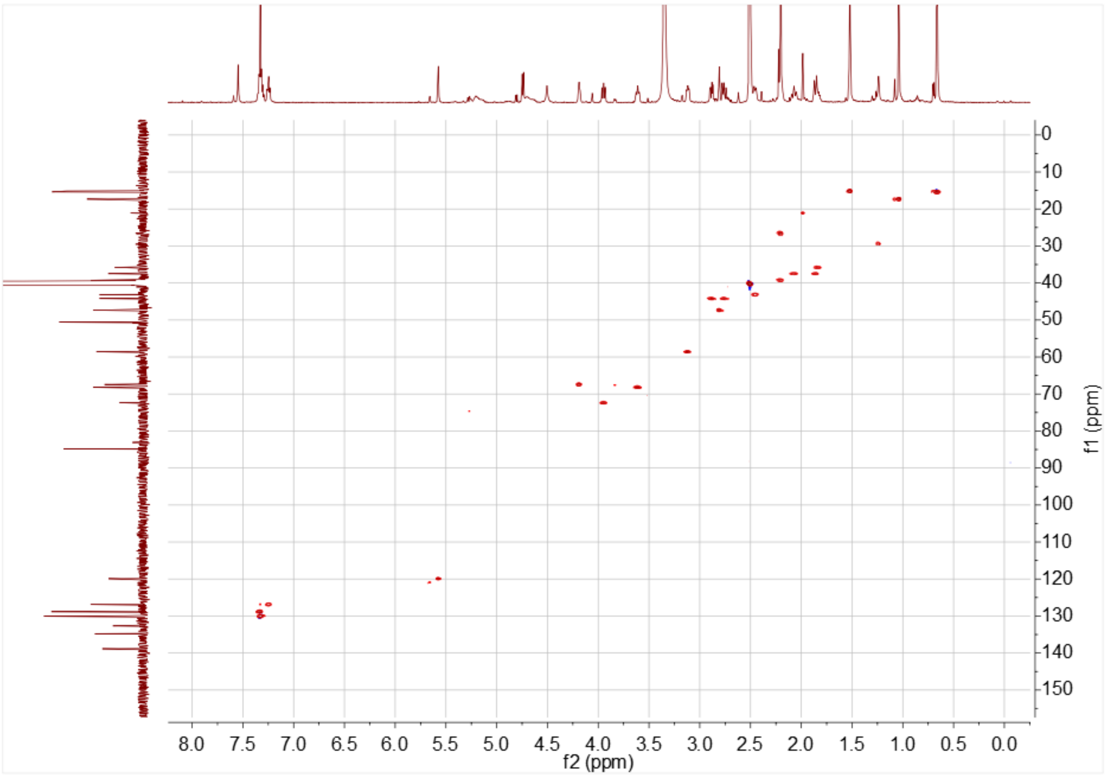

Figure S6.4 HMBC spectrum of **6** in DMSO-d_6

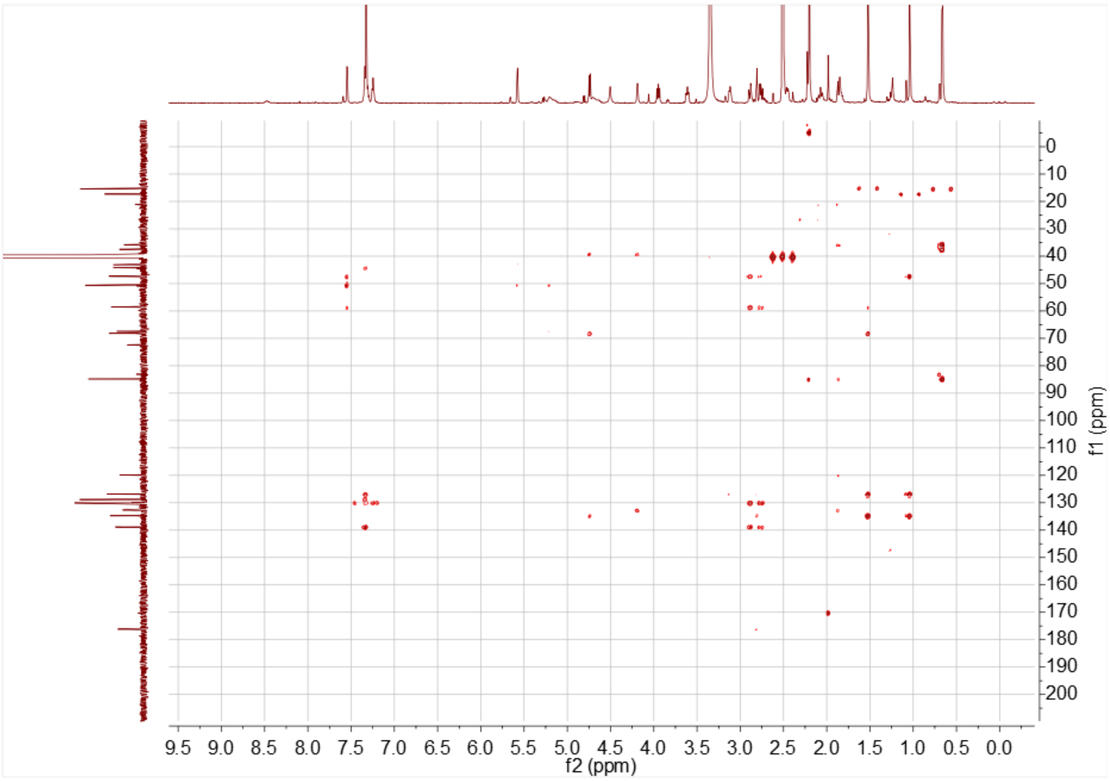

Figure S6.5 COSY spectrum of **6** in DMSO-d_6

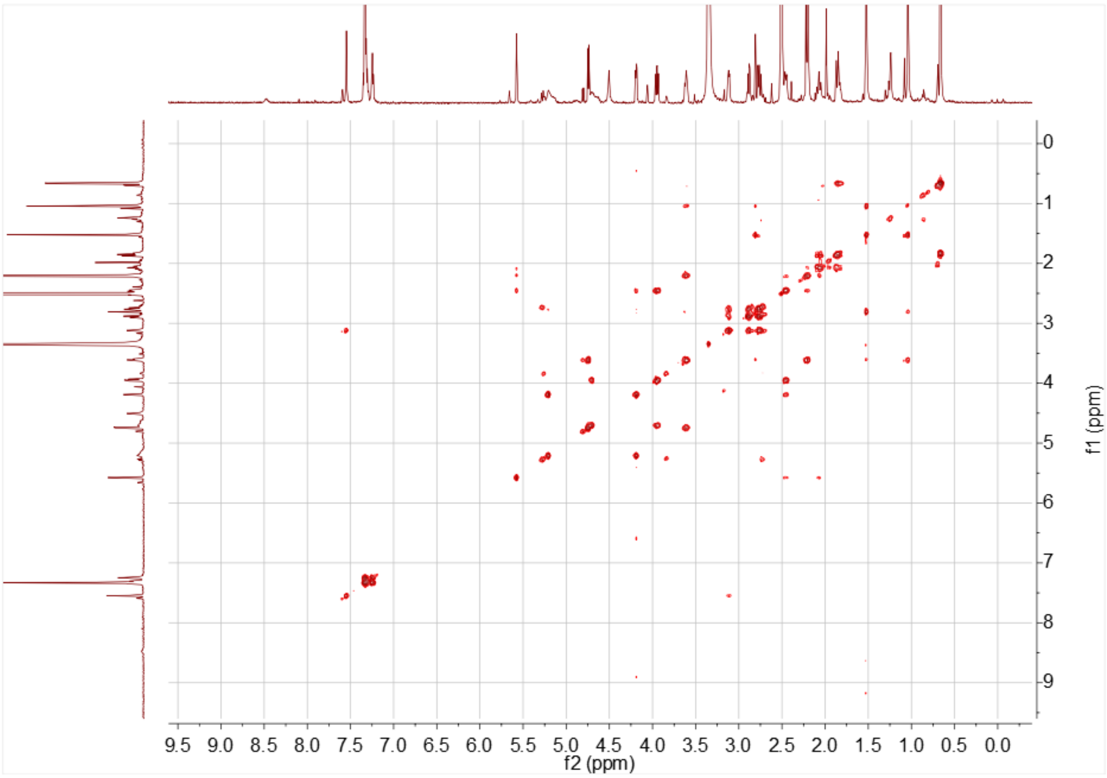

Figure S6.6 Roesy spectrum of **6** in DMSO-d_6

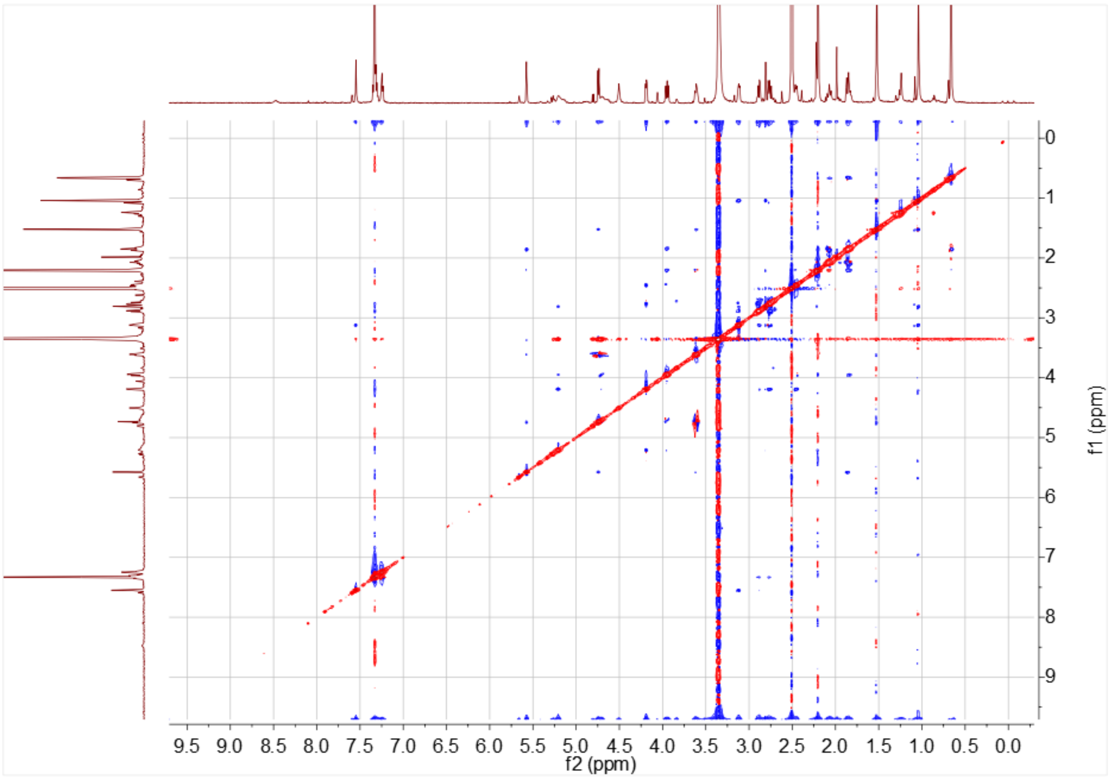

Figure S6.7 HRMS spectrum of **6**

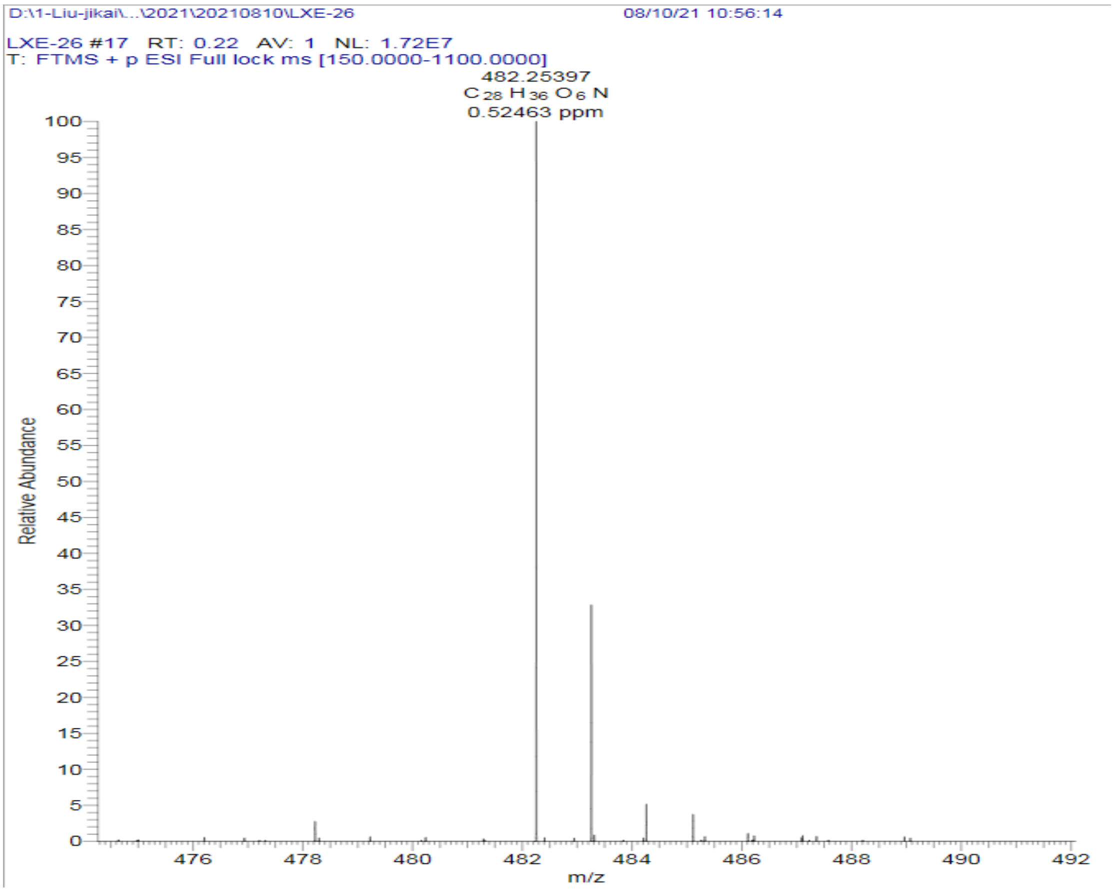

Quantum chemical calculation

The ECD calculation was carried out using Gaussian 16 software package. Systematic conformational analyses were performed via Spartan 14 using the MMFF94s molecular mechanics force field calculation with 3 kcal/mol of cutoff energy. The optimization and frequency of conformers were calculated on B3LYP-D3(BJ)/6-311G* level of theory with IEFPCM solvent model (MeOH) by using Gaussian 16. These primary conformations were subjected to theoretical calculations of ECD utilizing time-dependent density functional theory (TDDFT) calculations at the B3LYP-D3(BJ)/6-311G* level in MeOH. The ECD curves was simulated in SpecDis V1.71 using a Gaussian function. The calculated ECD data of all conformers were Boltzmann averaged by Gibbs free energy.

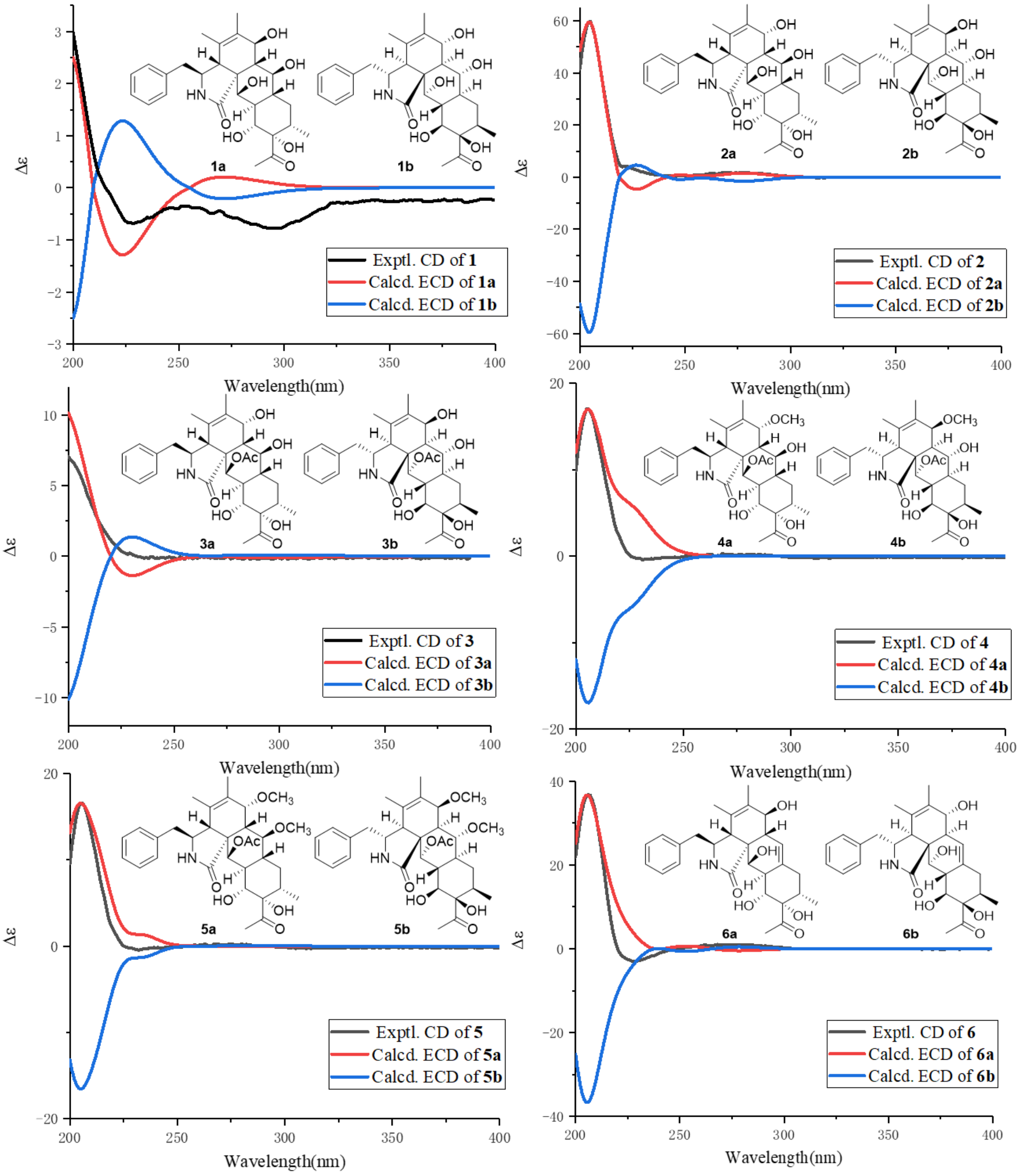

Figure S1. ECD calculations of **1−6**.

Table S1. Cartesian coordinates for the low-energy optimized conformers of **1a** at B3LYP-D3(BJ)/6-311G* level

conformer 1a_1							
C	-1.2284072	2.82156584	1.26878075	C	4.94582393	-2.7152129	-2.6210636
C	1.13852366	1.58881776	0.19096591	H	-1.4374297	0.73516901	1.5967229
C	1.01401138	3.126138	0.18871393	O	0.1082026	-3.2266082	-1.5026308
C	-0.1851098	3.62671256	0.96340921	H	0.97273819	3.51729809	-0.8338385
C	-0.0443751	-0.6169612	-0.2641873	H	-0.9461042	-1.061629	-0.6957561
C	1.15857082	-1.0869475	-1.1156597	H	2.28234224	1.51650798	-1.6718874
C	2.4691868	-0.4133423	-0.690195	H	1.51048088	-2.9404044	-0.0421641
C	2.34426176	1.11901084	-0.6531338	H	4.14041751	-2.6734476	-0.6452087
C	1.33551616	-2.6214314	-1.0752241	H	4.55376937	-0.3758719	-1.3068979
C	2.49154336	-3.1088546	-1.9773635	H	3.41549265	-0.4801096	-2.6431073
C	3.8098486	-2.3721617	-1.6479896	H	-3.2611898	2.16906437	-0.106027
C	3.61522178	-0.8453248	-1.6268953	H	-2.6028669	1.71169722	-2.320302
C	-0.1888877	0.92605931	-0.209836	H	-4.2137834	-0.071357	-0.7778012
C	-1.3639579	1.3892986	0.72154317	H	-2.9102966	-0.8829781	0.0361323
C	-2.625375	1.2802308	-0.187067	H	-2.9794221	-1.2259498	2.43328398
N	-2.0968034	1.32388872	-1.5319727	H	-4.2525323	-1.0668875	4.53921948
C	-0.7217379	1.36627996	-1.5622083	H	-6.2812671	0.35311495	4.67054088
O	-0.0690861	1.66282195	-2.5470846	H	-7.041441	1.61611553	2.67647461
C	-3.5008122	0.03788992	0.05101202	H	-5.7848001	1.46914122	0.55805424
C	-4.2864157	0.11210026	1.33844421	H	-2.6510203	2.40684698	2.83335014
C	-3.8688684	-0.6003709	2.47060441	H	-2.0343343	4.03846244	2.88847258
C	-4.5845078	-0.5119876	3.66541281	H	-3.2127622	3.59660046	1.63816859
C	-5.7239282	0.28649461	3.73979483	H	0.51361939	5.24585276	2.20170664
C	-6.1502188	0.99639188	2.61927569	H	0.26841468	5.67639988	0.49422392
C	-5.4359091	0.91008337	1.42357929	H	-1.1162118	5.51179176	1.54988939
C	-2.3431038	3.24566699	2.20065125	H	2.1456536	3.45170826	1.74211999
C	-0.130626	5.09156933	1.3305998	H	3.44280243	2.57935997	0.0471136
O	2.1967973	3.66565367	0.79169782	H	-0.209793	-2.0566891	1.03964864
H	1.36681357	1.29281589	1.22791289	H	3.12412287	-6.2916545	-0.5868561
O	3.56257531	1.61481064	-0.0854212	H	3.40965465	-4.7128964	0.15765024
O	0.07289704	-1.1228467	1.06861871	H	1.73557092	-5.3157604	-0.0617951
H	2.73573336	-0.7536835	0.32144642	H	2.5227395	-3.5217228	-3.8880361
H	0.93701742	-0.7992883	-2.1528174	H	5.16637895	-3.7869887	-2.6140882
C	2.67755532	-4.6386754	-1.8606538	H	4.70223308	-2.4226181	-3.6476439
O	2.79118208	-5.3254358	-2.8829209	H	5.86593741	-2.1930117	-2.3363326
C	2.73711937	-5.2724949	-0.4952892	H	0.10435778	-3.1384161	-2.4805562
O	2.09825697	-2.8294112	-3.3373231				

conformer 1a_2							
C	-0.7225607	3.10906265	1.62281978	C	4.71781208	-3.3164783	-1.9836849
C	1.46199227	1.57629027	0.5431688	H	-1.0811046	1.09437702	2.190211
C	1.4550631	3.10955303	0.37859447	O	-0.0535049	-3.2906144	-0.5200816
C	0.35769527	3.78637589	1.171074	H	1.36613646	3.3924935	-0.6761278

C	0.08605139	-0.5580501	0.42259352	H	-0.8769026	-0.9722931	0.1097389
C	1.1834916	-1.2187434	-0.4443509	H	2.46058821	1.20546305	-1.3674767
C	2.56919546	-0.6146346	-0.1837355	H	1.46407129	-2.9662045	0.81213676
C	2.56472261	0.91795271	-0.3157659	H	4.06104733	-2.9871528	0.01996156
C	1.24377314	-2.748749	-0.2384173	H	4.60021477	-0.8204703	-0.9314197
C	2.29285963	-3.4285706	-1.14695	H	3.36339408	-0.9771887	-2.1715235
C	3.68364975	-2.7729322	-0.9886514	H	-2.8818729	2.49921634	0.45631228
C	3.60799676	-1.2423482	-1.1343198	H	-2.4482089	1.70327037	-1.7273413
C	0.06279219	0.98771949	0.30296418	H	-2.8184749	-0.5549821	0.80692189
C	-1.0048338	1.64565703	1.24514046	H	-3.4543717	0.55633519	1.99511517
C	-2.3297227	1.55258589	0.43398458	H	-5.5073064	1.90751299	1.52352001
N	-1.8965119	1.39504617	-0.9339169	H	-7.6610437	1.94908206	0.31905887
C	-0.5294894	1.31474004	-1.0577747	H	-7.9984597	0.53168485	-1.6874133
O	0.07337391	1.43390519	-2.1094331	H	-6.1676289	-0.9354127	-2.4915773
C	-3.2685493	0.43580065	0.91981565	H	-4.0051869	-0.9907735	-1.3026174
C	-4.5988985	0.45042379	0.20169081	H	-2.1506017	2.97143896	3.23304796
C	-5.6385334	1.27880884	0.64588376	H	-1.3046537	4.50183139	3.19051394
C	-6.8579942	1.30631225	-0.0323259	H	-2.5700472	4.16568165	1.98801926
C	-7.0482787	0.5100424	-1.1601354	H	1.28317173	5.4585753	2.16624833
C	-6.0197378	-0.3146328	-1.6115611	H	0.91081021	5.73560453	0.44975187
C	-4.799697	-0.3443901	-0.9351023	H	-0.3762258	5.78994972	1.63447512
C	-1.7423854	3.72730222	2.553233	H	2.72654135	3.49271705	1.80591838
C	0.55091569	5.27132902	1.37473022	H	3.82048475	2.34918105	0.13552143
O	2.71668786	3.60995872	0.83759749	H	-0.0948694	-1.8151961	1.90258782
H	1.74039451	1.37462648	1.59049661	H	3.23430463	-4.8548554	1.1004274
O	3.85465217	1.36911842	0.114277	H	1.50850419	-5.3383652	1.05582375
O	0.26743156	-0.9149486	1.79592795	H	2.77977393	-6.4778299	0.56412659
H	2.88155327	-0.8626841	0.84156276	H	2.157786	-4.0525295	-2.9948939
H	0.90966006	-1.0298775	-1.4916011	H	4.42468353	-3.1206515	-3.0202857
C	2.36968016	-4.9460227	-0.8640523	H	5.69306208	-2.8449446	-1.8196101
O	2.35785028	-5.749598	-1.8041122	H	4.85586351	-4.3955134	-1.8652876
C	2.4775239	-5.4264188	0.55969854	H	-0.1157521	-3.3121982	-1.4997967
O	1.82615189	-3.2707716	-2.5033485				
conformer **1a**_3							
C	-0.8226621	3.02739988	1.19882688	C	3.89567862	-3.1950311	-3.6023851
C	1.08657725	1.59529494	-0.4238379	H	-0.8502283	1.01905304	1.87632022
C	0.95858701	3.12633344	-0.5743055	O	-0.3222226	-3.388296	-0.9428932
C	0.05128144	3.75216669	0.46336189	H	0.59745259	3.39225085	-1.5744571
C	-0.1258852	-0.6199884	-0.1279337	H	-1.1083495	-1.0999014	-0.1706754
C	0.74350313	-1.2428121	-1.2476686	H	1.44657505	1.19504954	-2.5416298
C	2.10120599	-0.5366831	-1.3925995	H	1.46918391	-2.9199529	-0.0751938
C	1.91532751	0.98075384	-1.5757261	H	3.74739384	-2.8157173	-1.5070085
C	0.96161252	-2.7573972	-1.0320533	H	3.88481791	-0.6724407	-2.6268568
C	1.77528117	-3.4073233	-2.1721533	H	2.38530162	-0.9465221	-3.5051076
C	3.1137114	-2.6678629	-2.3914855	H	-3.1784752	2.23048024	0.69654266
C	2.90091251	-1.1515979	-2.557653	H	-3.2852673	1.45082146	-1.5185461

C	-0.2835966	0.91969357	-0.2508051	H	-4.2284354	-0.0726176	0.70275784
C	-1.091536	1.53752363	0.94237501	H	-2.7051928	-0.7726571	1.16097297
C	-2.5807627	1.32621746	0.53562941	H	-1.9702173	-0.7271165	3.47310552
N	-2.5352789	1.16269875	-0.9003952	H	-2.4997039	-0.2284426	5.82904444
C	-1.2511217	1.17231913	-1.3945794	H	-4.4367046	1.21130116	6.39845827
O	-0.9761784	1.30067333	-2.5748812	H	-5.8542468	2.15242777	4.5944984
C	-3.2896825	0.14971487	1.22864919	H	-5.3445866	1.66416866	2.23015796
C	-3.6189343	0.43143633	2.67507106	H	-1.654663	2.87560302	3.18124563
C	-2.827332	-0.0977646	3.70314508	H	-1.0722519	4.4771852	2.80893691
C	-3.1213679	0.18331948	5.03809961	H	-2.5876694	3.9120322	2.0794056
C	-4.2089813	0.99318641	5.35834247	H	1.08710216	5.49424999	1.19769518
C	-5.0046688	1.52221815	4.34435265	H	0.31367356	5.71069619	-0.389184
C	-4.7121324	1.2432266	3.00870041	H	-0.6677	5.72589823	1.0570318
C	-1.5783707	3.61055811	2.37335677	H	2.89487533	3.10826223	-0.8086245
C	0.20402712	5.24989342	0.59905875	H	3.58985012	1.44799586	-2.4790856
O	2.24666352	3.72876338	-0.4140775	H	0.14558339	-1.8396354	1.3698364
H	1.67058923	1.42425892	0.49524045	H	2.86901707	-6.3749472	-0.6022259
O	3.20393288	1.60944568	-1.5982432	H	3.36118474	-4.7162358	-0.2340378
O	0.43857833	-0.9355621	1.14759005	H	1.71358561	-5.2823466	0.1896292
H	2.69271698	-0.6974484	-0.4795046	H	1.17434495	-4.1150861	-3.8929561
H	0.18248369	-1.1124316	-2.1837513	H	3.33494823	-3.0665293	-4.5339969
C	2.01515606	-4.9087153	-1.8963474	H	4.84523198	-2.6590518	-3.7093297
O	1.80692523	-5.7376026	-2.7902302	H	4.13447176	-4.2570785	-3.4907468
C	2.52036029	-5.3396214	-0.5443151	H	-0.6538149	-3.4386009	-1.8652851
O	0.96851489	-3.313554	-3.3646661				
conformer 1a_4							
C	-0.5306435	3.17912163	1.62964555	C	4.40464227	-3.2957702	-2.581849
C	1.47808309	1.65271817	0.22628937	H	-0.7202142	1.18330669	2.3207528
C	1.42791162	3.18528738	0.05090272	O	-0.0662891	-3.2814922	-0.3672372
C	0.44604716	3.85880056	0.98656287	H	1.18039316	3.45262537	-0.9828984
C	0.15892335	-0.5088881	0.43057161	H	-0.8321192	-0.9548155	0.30384342
C	1.10941292	-1.1843267	-0.5880005	H	2.03464163	1.20413972	-1.8388695
C	2.50141445	-0.5317517	-0.6061532	H	1.64284605	-2.8664553	0.67674932
C	2.39448828	0.98865429	-0.8276593	H	4.06060937	-2.8705947	-0.5185256
C	1.24423899	-2.7017666	-0.3299921	H	4.39229055	-0.7559742	-1.653438
C	2.14431246	-3.4025682	-1.3708508	H	2.97813429	-0.9919113	-2.6730482
C	3.52540588	-2.7169008	-1.4649136	H	-2.8412412	2.47944385	0.88892133
C	3.38874814	-1.197372	-1.6756866	H	-2.7811986	1.61130674	-1.3044812
C	0.07202675	1.03237305	0.26518451	H	-2.6247436	-0.5545408	1.33809937
C	-0.8295707	1.69947041	1.35922199	H	-3.0765943	0.59232699	2.57556631
C	-2.2744376	1.54518015	0.80265759	H	-5.2170026	1.88459377	2.41114653
N	-2.0889427	1.34097077	-0.6143765	H	-7.5496117	1.82193433	1.60737781
C	-0.7636172	1.29343669	-0.977551	H	-8.1946702	0.2980187	-0.2394655
O	-0.363834	1.37959859	-2.1254428	H	-6.4927971	-1.1708816	-1.2869299
C	-3.078856	0.42924706	1.48986136	H	-4.1537344	-1.1221982	-0.4990884
C	-4.5145803	0.38057859	1.01845587	H	-1.6761716	3.07163668	3.45428554

C	-5.4830783	1.20938868	1.60105232	H	-0.8659135	4.60624288	3.23851506
C	-6.8031941	1.1780518	1.14934119	H	-2.3060196	4.2300554	2.26569303
C	-7.1662432	0.32195011	0.11132287	H	1.48553383	5.5656341	1.79483514
C	-6.2100115	-0.5036751	-0.4767305	H	0.85125296	5.79917553	0.1494971
C	-4.8894957	-0.4745542	-0.0267634	H	-0.2405886	5.86330978	1.51454272
C	-1.3923083	3.81277988	2.69907931	H	3.37532875	3.09007557	0.01018679
C	0.64478364	5.35070709	1.12780714	H	4.16909832	1.37586504	-1.5614468
O	2.71625111	3.73821458	0.33664608	H	0.23300459	-1.6967469	1.97714279
H	1.95835775	1.47216752	1.20192997	H	3.47349405	-4.7293751	0.73968808
O	3.70220821	1.56837337	-0.727248	H	1.77163896	-5.2294174	1.00086046
O	0.58823006	-0.8137971	1.7602969	H	2.96087272	-6.3765246	0.34828872
H	2.99219016	-0.6982503	0.36386273	H	1.6952109	-4.1207589	-3.1332601
H	0.64988242	-1.0505613	-1.5773317	H	3.9461093	-3.1648063	-3.5674585
C	2.2975663	-4.9060671	-1.0486463	H	5.38005824	-2.7971786	-2.6001611
O	2.14950781	-5.7440699	-1.9460206	H	4.58993552	-4.3635031	-2.4300631
C	2.64633381	-5.328746	0.35441765	H	-0.2999562	-3.337561	-1.3188681
O	1.46625902	-3.3029606	-2.6406466				

conformer 1a_5							
C	-0.4987198	3.21259912	0.79523314	C	3.68742713	-4.0208226	-2.9493119
C	1.31768556	1.38945925	-0.4867078	H	-0.8528317	1.31152006	1.66781813
C	1.38473592	2.89040392	-0.8383925	O	-0.7039861	-3.4120387	-0.6542098
C	0.51016277	3.73751735	0.06163183	H	1.10473637	3.07041422	-1.8827413
C	-0.187241	-0.6198924	-0.074126	H	-1.2186069	-0.9797667	-0.134945
C	0.66430679	-1.4633518	-1.0528402	H	1.84889012	0.71068652	-2.4896303
C	2.1125829	-0.9609273	-1.131244	H	1.06198863	-3.0931409	0.3249169
C	2.16836463	0.54153646	-1.4562063	H	3.44279102	-3.4265293	-0.9139913
C	0.65682464	-2.9639597	-0.6844143	H	3.94511539	-1.4266535	-2.1992581
C	1.46016364	-3.8233507	-1.6865735	H	2.48935789	-1.600812	-3.170527
C	2.8993963	-3.2845772	-1.8575239	H	-2.8918679	2.66712798	0.15274748
C	2.90569784	-1.7766478	-2.169723	H	-2.9312904	1.64401905	-1.9655578
C	-0.1363837	0.90213733	-0.3738228	H	-4.2501303	0.54052703	0.31150121
C	-0.9471214	1.74818852	0.66782725	H	-2.8776372	-0.2978893	0.97008827
C	-2.4139516	1.68091651	0.14703726	H	-2.3341961	-0.1073619	3.32366806
N	-2.2777312	1.34192735	-1.2517439	H	-2.9589303	0.72075516	5.56067914
C	-0.9693007	1.13429276	-1.6225919	H	-4.6918966	2.47727526	5.8054908
O	-0.5864004	1.09037337	-2.7783212	H	-5.8092035	3.40488717	3.79546446
C	-3.3303027	0.69517642	0.89223798	H	-5.2020596	2.58900698	1.54752926
C	-3.7213245	1.18018316	2.26761752	H	-1.4688087	3.38480691	2.71181426
C	-3.0997617	0.66054714	3.41098763	H	-0.6670003	4.85437012	2.22138664
C	-3.4481742	1.12727721	4.67921549	H	-2.1911055	4.39300813	1.43888373
C	-4.4212242	2.11494193	4.81717207	H	1.72579062	5.40275929	0.68896585
C	-5.048228	2.63622637	3.68746613	H	1.07988867	5.54213491	-0.962266
C	-4.7010669	2.17156194	2.41825387	H	0.02886707	5.83936088	0.40222491
C	-1.2487326	4.00991643	1.84029118	H	3.28170159	2.76942662	-1.2411946
C	0.85340161	5.20927604	0.05699416	H	3.85827855	0.78567007	-0.4855635
O	2.72993788	3.34540227	-0.6724537	H	-0.1666929	-1.672617	1.56702593

H	1.79077857	1.28136782	0.50297568	H	2.70329928	-5.1104928	0.49841181
O	3.53553142	0.9718693	-1.3878507	H	0.96842734	-5.4283869	0.82045444
O	0.26228718	-0.8493507	1.26480887	H	2.03266327	-6.7266411	0.23913752
H	2.60134979	-1.1178251	-0.158609	H	0.92007359	-4.6109867	-3.3931177
H	0.20062656	-1.3573396	-2.0433163	H	3.21786974	-3.9104192	-3.9322358
C	1.47845571	-5.3071297	-1.2548652	H	4.70610175	-3.6239638	-3.0212509
O	1.23368791	-6.1921305	-2.0831385	H	3.77116869	-5.0892641	-2.7283667
C	1.81637904	-5.6564086	0.17095782	H	-0.9640932	-3.5175044	-1.5950841
O	0.75920803	-3.7526724	-2.9452002				
conformer 1a_6							
C	-0.9651817	3.20739242	1.32007921	C	4.96380929	-3.1981021	-1.4504897
C	1.34320274	1.71532545	0.47261764	H	-1.2515864	1.23125838	2.03921321
C	1.27683893	3.22901823	0.18221571	O	0.11680239	-3.2966655	-0.2618909
C	0.10500476	3.90084994	0.86745636	H	1.22492397	3.42846256	-0.8943851
C	0.08567447	-0.4940664	0.48094329	H	-0.8429016	-0.9864923	0.17768241
C	1.25081025	-1.166078	-0.2838294	H	2.41796417	1.21907209	-1.35885
C	2.59164338	-0.4712318	-0.0079322	H	1.53873473	-2.7967079	1.11981643
C	2.50819148	1.04044446	-0.2825244	H	4.16955032	-2.7433992	0.47769911
C	1.3691842	-2.6709798	0.04512584	H	4.66882987	-0.6331141	-0.6195417
C	2.49864114	-3.3637635	-0.7504618	H	3.51801299	-0.9524586	-1.9105621
C	3.84661447	-2.629068	-0.565529	H	-3.0278575	2.39587737	0.10864152
C	3.71145285	-1.1203059	-0.8427411	H	-2.4528102	1.43576281	-1.9706206
C	-0.0122888	1.03343268	0.2241368	H	-2.8422568	-0.6080593	0.73874954
C	-1.1563592	1.70758571	1.05576753	H	-3.5884579	0.57094113	1.7898789
C	-2.433562	1.47898525	0.19722299	H	-5.6709943	1.77734482	1.09560519
N	-1.9275892	1.22114147	-1.1298203	H	-7.7618898	1.6013674	-0.2035821
C	-0.5539448	1.20940575	-1.1848785	H	-7.937028	-0.0135248	-2.0772039
O	0.09244213	1.26661679	-2.2158334	H	-6.0061138	-1.4597785	-2.6524665
C	-3.3435442	0.36455444	0.73975535	H	-3.9050375	-1.2984202	-1.3669076
C	-4.6355019	0.24714309	-0.0365314	H	-2.4649365	3.12203996	2.86776165
C	-5.7311145	1.06266101	0.27798687	H	-1.6833616	4.68197514	2.7513258
C	-6.915322	0.96735848	-0.4543555	H	-2.8710922	4.2014552	1.51837523
C	-7.0143095	0.06009634	-1.5075676	H	0.90793386	5.67266598	1.79498937
C	-5.9295027	-0.7528236	-1.8302985	H	0.61490383	5.82307333	0.04700657
C	-4.7444787	-0.6598464	-1.0994592	H	-0.732975	5.89608009	1.1594719
C	-2.0549461	3.84426121	2.15327188	H	3.21250888	3.38324384	0.24061397
C	0.2268582	5.40294027	0.98175069	H	3.87562863	1.45232429	1.06590741
O	2.46745152	3.84780805	0.67526126	H	-0.1295378	-1.6031066	2.07130349
H	1.58238018	1.61698435	1.54415639	H	3.01358333	-6.2551809	1.20879939
O	3.74619784	1.64548386	0.11786394	H	3.36571078	-4.5779874	1.64686956
O	0.24609257	-0.7213509	1.88467967	H	1.66777411	-5.1438558	1.54029106
H	2.85832971	-0.6110278	1.04984982	H	2.49070957	-4.1307875	-2.5494467
H	1.01943996	-1.0732606	-1.3538679	H	4.72570363	-3.1029627	-2.5149448
C	2.62633625	-4.8512026	-0.3510309	H	5.90426796	-2.6653087	-1.2714887
O	2.71023646	-5.7189853	-1.2280115	H	5.14442993	-4.2553258	-1.2335863
C	2.6678648	-5.2221204	1.108365	H	0.10577485	-3.3892201	-1.2393317

O	2.10803657	-3.3258647	-2.1385371				
conformer 1a_7							
C	-0.9860546	3.00157113	1.01267091	C	4.52458657	-3.2572859	-2.7640066
C	1.18628451	1.55034558	-0.170397	H	-1.1983706	0.9892877	1.65208872
C	1.07931772	3.07343529	-0.4090832	O	-0.15687	-3.4125397	-1.0416512
C	0.01945311	3.72115235	0.46033794	H	0.87388192	3.29298399	-1.4630535
C	-0.0898373	-0.6574873	-0.1576335	H	-1.0480373	-1.1199192	-0.4133887
C	0.98652769	-1.2856105	-1.0728402	H	2.10832437	1.17161758	-2.1118139
C	2.35452703	-0.6157032	-0.8994002	H	1.41551571	-2.9931705	0.19700822
C	2.27610299	0.9117332	-1.0609757	H	3.96122071	-2.9167403	-0.7339343
C	1.1276525	-2.8071441	-0.8431956	H	4.35091415	-0.7487971	-1.7516028
C	2.15518907	-3.4584271	-1.7957536	H	3.05832791	-0.9845814	-2.920095
C	3.52145842	-2.7397468	-1.7243333	H	-3.1953998	2.25159994	0.01176424
C	3.36907583	-1.2174796	-1.8928771	H	-2.8437223	1.46615847	-2.1839579
C	-0.1916782	0.88244765	-0.3034285	H	-4.2708775	-0.0273173	-0.2211552
C	-1.224916	1.51515221	0.69186118	H	-2.8928613	-0.7637266	0.54001203
C	-2.595947	1.33475819	-0.0250959	H	-2.6609864	-0.748142	2.95424748
N	-2.246237	1.17412144	-1.4189179	H	-3.6616382	-0.2480678	5.1516262
C	-0.8862086	1.15275803	-1.6271177	H	-5.6422893	1.2350383	5.31625553
O	-0.3601409	1.28749478	-2.717867	H	-6.6287902	2.21787045	3.2642982
C	-3.4591228	0.17119464	0.49218527	H	-5.6455096	1.72847563	1.05456325
C	-4.0785598	0.45439166	1.83983269	H	-2.1879319	2.83230463	2.79477664
C	-3.532391	-0.0985211	3.00587563	H	-1.5236858	4.42888583	2.57226667
C	-4.0935299	0.18330112	4.25214146	H	-2.8767851	3.89920649	1.55197073
C	-5.2056749	1.01749476	4.34499439	H	0.9503153	5.44630416	1.35178658
C	-5.7590105	1.56998798	3.19180739	H	0.38985832	5.68906147	-0.3194138
C	-5.1991078	1.29032312	1.94451888	H	-0.7685091	5.69072011	0.98686186
C	-1.9471825	3.57824072	2.03036404	H	2.36089378	4.54410272	-0.481146
C	0.15240979	5.21472071	0.63940578	H	3.44492066	2.34806193	-0.4167836
O	2.35480309	3.64833932	-0.1002597	H	-0.1283674	-1.9010924	1.34391605
H	1.53398374	1.41620679	0.86703612	H	3.28008676	-4.7980474	0.42093058
O	3.5698359	1.41981728	-0.7097611	H	1.57729301	-5.3558779	0.48001813
O	0.17738668	-0.9843981	1.20845907	H	2.8687179	-6.44932	-0.0610614
H	2.73158022	-0.8274594	0.11219811	H	1.94792605	-4.1257744	-3.6213945
H	0.64760933	-1.1295024	-2.1064003	H	5.4849549	-2.7400363	-2.6614318
C	2.31485466	-4.9651613	-1.4910234	H	4.71723197	-4.3265728	-2.6339621
O	2.28809992	-5.7869313	-2.4149981	H	4.16811516	-3.0956555	-3.7865627
C	2.52084804	-5.412116	-0.0671852	H	-0.273929	-3.4541564	-2.0155748
O	1.610074	-3.3481584	-3.1275463				
conformer 1a_8							
C	-0.4302685	3.27720458	1.35357659	C	4.36680972	-3.712336	-2.0987137
C	1.55685339	1.56080081	0.19915908	H	-0.7772445	1.3502027	2.1746781
C	1.55375756	3.06195419	-0.1664523	O	-0.2117585	-3.3318546	-0.1514384
C	0.59964809	3.86320692	0.69828709	H	1.30259222	3.2084937	-1.2232531
C	0.11963737	-0.5250809	0.47811766	H	-0.8845115	-0.9299227	0.32058293
C	1.08981767	-1.3172054	-0.4282351	H	2.33864285	0.94148875	-1.7422747

C	2.51296102	-0.748278	-0.3849177	H	1.45157484	-2.936292	0.97161221
C	2.54351756	0.76092251	-0.681485	H	3.9445443	-3.1475607	-0.0844595
C	1.12483171	-2.8190113	-0.0670838	H	4.44177946	-1.1178338	-1.3186502
C	2.0462783	-3.6334182	-1.0027095	H	3.07088245	-1.350904	-2.3948558
C	3.46533735	-3.0244647	-1.0646719	H	-2.7311936	2.6420892	0.52317964
C	3.4206192	-1.5159632	-1.3670465	H	-2.5921474	1.59732468	-1.5970596
C	0.12645351	0.99822289	0.19213838	H	-2.7159503	-0.3592345	1.19439229
C	-0.7998752	1.79584787	1.17276226	H	-3.1716685	0.89639705	2.32011271
C	-2.2149894	1.67499462	0.53866553	H	-5.224721	2.27843573	1.94302047
N	-1.9556238	1.35609259	-0.8452761	H	-7.5058307	2.28369719	1.00055629
C	-0.616838	1.20177885	-1.1180006	H	-8.1250613	0.67547193	-0.7825069
O	-0.1415665	1.18565125	-2.2397148	H	-6.449404	-0.9469778	-1.6257941
C	-3.1203276	0.65586286	1.25015109	H	-4.1620342	-0.9678118	-0.6979499
C	-4.5265162	0.65082077	0.69464583	H	-1.650131	3.3415264	3.13268607
C	-5.4796714	1.56601074	1.1618444	H	-0.751425	4.81577276	2.85963979
C	-6.7705892	1.57327267	0.63148819	H	-2.1712511	4.45246438	1.85062336
C	-7.1192344	0.66975973	-0.3706881	H	1.72621908	5.56623057	1.38188137
C	-6.177858	-0.2421274	-0.8440657	H	1.06112843	5.72995013	-0.2604381
C	-4.8866439	-0.2518295	-0.3152404	H	0.01015802	5.92527837	1.12219389
C	-1.2980222	4.01858083	2.34667535	H	2.93869945	4.40809556	-0.4443748
C	0.86097776	5.34979508	0.74753212	H	3.85323613	2.14823321	-0.2294484
O	2.88492702	3.55578341	0.02300844	H	0.07175656	-1.6121223	2.09745189
H	1.94975162	1.48730739	1.22651318	H	3.18500261	-4.8556236	1.27330205
O	3.88981175	1.19053809	-0.4401843	H	1.4509798	-5.2687685	1.46473008
O	0.44517387	-0.7442147	1.85314337	H	2.6255324	-6.505273	0.96694822
H	2.92903306	-0.9007019	0.62207398	H	1.68958869	-4.4414542	-2.7466963
H	0.705849	-1.2256608	-1.4536692	H	3.96771588	-3.613936	-3.1135324
C	2.10711555	-5.114952	-0.5668436	H	5.36815257	-3.2677764	-2.0911062
O	1.96693926	-6.0110026	-1.4077965	H	4.48396906	-4.7779009	-1.8790778
C	2.35644293	-5.4483722	0.88097142	H	-0.3807335	-3.4508964	-1.1112814
O	1.4383916	-3.5987991	-2.3113532				

conformer 1a_9							
C	-1.3874387	3.09553592	1.83914769	C	4.47087974	-2.1084957	-2.8792231
C	0.82193679	2.03118437	0.34998284	H	-1.5461057	0.99080088	2.07248273
C	0.60177685	3.55598598	0.38570343	O	-0.1450543	-2.8678375	-1.1691234
C	-0.452471	3.96547561	1.39314904	H	0.33097038	3.9419161	-0.6030874
C	-0.2659547	-0.2461638	0.11158247	H	-1.1915192	-0.7596827	-0.1541755
C	0.81569929	-0.6629492	-0.91339	H	1.67056611	1.98820982	-1.6674509
C	2.13049577	0.09823396	-0.6929829	H	1.3934209	-2.4803745	0.12270929
C	1.91337707	1.62170919	-0.6645536	H	3.91949458	-2.0528179	-0.8181162
C	1.08305626	-2.1846066	-0.8850113	H	4.10237843	0.24495668	-1.5947329
C	2.14545592	-2.6218335	-1.918983	H	2.80018048	0.02871797	-2.7566822
C	3.44747955	-1.802302	-1.77733	H	-3.3983409	2.63923087	0.50539622
C	3.16097675	-0.2904121	-1.7699111	H	-3.2093726	1.81310652	-1.6528818
C	-0.5081338	1.28488856	0.15354716	H	-4.2942631	1.03611805	2.03220854
C	-1.5693966	1.69391392	1.23368721	H	-4.9137107	0.79087842	0.42104135

C	-2.9303741	1.6451726	0.47934121	H	-4.1193214	-1.1818666	-0.9678568
N	-2.6034109	1.47756536	-0.9124851	H	-3.5382284	-3.5759799	-0.8310218
C	-1.2505291	1.63411937	-1.1246231	H	-2.7586682	-4.5543405	1.3057953
O	-0.7643221	1.95128547	-2.1956968	H	-2.5666363	-3.1266715	3.32740361
C	-4.0047015	0.69166027	1.03038948	H	-3.1316312	-0.7294489	3.21156767
C	-3.6593822	-0.7729201	1.10906752	H	-2.5030017	2.5351764	3.59413656
C	-3.7820984	-1.5943196	-0.019457	H	-1.9673323	4.19883763	3.62832333
C	-3.4562973	-2.9493778	0.05354924	H	-3.316072	3.73538225	2.56957944
C	-3.0143587	-3.4991564	1.25518092	H	0.39827784	5.563812	2.56104429
C	-2.9027101	-2.696193	2.38756483	H	-0.1891535	6.0547411	0.95591735
C	-3.2246447	-1.3400292	2.31594996	H	-1.3358547	5.77055123	2.24534579
C	-2.3464707	3.41729747	2.96355268	H	2.01484265	3.92285623	1.67667301
C	-0.3935157	5.41346801	1.82066315	H	3.01004221	3.17062137	-0.194444
O	1.83843215	4.17905438	0.75184796	H	-0.3041832	-1.5745777	1.52499872
H	1.22318456	1.75465504	1.3390085	H	3.14548884	-5.7242706	-0.5658045
O	3.16827649	2.20861584	-0.2997513	H	3.40674758	-4.1130587	0.11540293
O	0.11563819	-0.6982942	1.41476725	H	1.76421565	-4.831845	0.10690147
H	2.55741494	-0.197579	0.27691995	H	1.95262981	-3.0883752	-3.8075894
H	0.42473251	-0.4096439	-1.9083463	H	4.75721939	-3.1646064	-2.8748178
C	2.437637	-4.1348879	-1.8015447	H	4.0822026	-1.8633683	-3.8730304
O	2.46853314	-4.8379246	-2.818878	H	5.38517828	-1.5243327	-2.7268914
C	2.7020944	-4.7316282	-0.443826	H	-0.2855141	-2.7548516	-2.1332697
O	1.56924031	-2.3987119	-3.224561				

conformer 1a_10							
C	-1.7913324	2.59451883	1.10957217	C	5.48834329	-1.9042925	-2.1517582
C	0.80553505	1.77476477	0.2006928	H	-1.6544127	0.51019328	1.49629202
C	0.41888857	3.26669202	0.12757716	O	0.68260539	-3.1037907	-1.6822861
C	-0.8910568	3.56450189	0.82563818	H	0.36680612	3.61339925	-0.9101034
C	0.05727983	-0.6133286	-0.247296	H	-0.7248942	-1.2235347	-0.7048894
C	1.35524868	-0.8720792	-1.0495744	H	2.09648923	1.86674708	-1.5658228
C	2.50928951	-0.0047673	-0.5329401	H	1.81473799	-2.7230448	-0.0155929
C	2.13487735	1.49053744	-0.537561	H	4.44716427	-1.9980895	-0.2902391
C	1.74028928	-2.3682278	-1.0487812	H	4.60948872	0.34942822	-0.9537407
C	3.05413963	-2.6489784	-1.8140095	H	3.63199574	0.07451393	-2.3894969
C	4.20350817	-1.7377366	-1.3287738	H	-3.6099333	1.55384631	-0.3309506
C	3.78683262	-0.256135	-1.3540059	H	-2.7551469	1.19547448	-2.5062873
C	-0.3615947	0.87950892	-0.248051	H	-4.1553343	-0.789547	-0.9874399
C	-1.6489986	1.14602664	0.60494086	H	-2.7315195	-1.3888065	-0.1962324
C	-2.818413	0.79680846	-0.3651454	H	-2.7506415	-1.8529873	2.16382638
N	-2.2320042	0.92819919	-1.6800749	H	-4.0163002	-1.9721697	4.27868471
C	-0.8843406	1.1986286	-1.639123	H	-6.2313121	-0.8726671	4.47483671
O	-0.2321678	1.59128246	-2.5902644	H	-7.1861916	0.34571445	2.53734151
C	-3.4682755	-0.5816913	-0.1555265	H	-5.9388487	0.47390247	0.41155224
C	-4.2493087	-0.6747295	1.13306853	H	-3.2057934	1.97331705	2.61230363
C	-3.7219756	-1.3651536	2.23288287	H	-2.8817565	3.6882712	2.64814231
C	-4.4330426	-1.4335855	3.43147384	H	-3.8967812	3.02210006	1.35427609

C	−5.6771149	−0.8156674	3.54163997	H	−0.5646643	5.3180995	2.03544094
C	−6.2128696	−0.1307105	2.45275151	H	−0.7839482	5.64837296	0.30234844
C	−5.5035566	−0.0605602	1.25297318	H	−2.1755168	5.26692858	1.2909966
C	−3.0121499	2.83917264	1.97057053	H	1.35077529	3.89217994	1.72614797
C	−1.1197496	5.02535333	1.13890659	H	2.93799673	3.12013536	0.18404028
O	1.45718431	4.02014071	0.76546447	H	0.89117315	−0.591232	1.55381034
H	1.00804347	1.56337926	1.2631129	H	3.98949008	−5.7479208	−0.4093241
O	3.20113329	2.17602981	0.1280739	H	3.91768602	−4.172906	0.39277205
O	0.19010626	−1.0869877	1.09852732	H	2.40515515	−5.0239945	−0.0583434
H	2.74586271	−0.2923125	0.50147209	H	3.34311603	−3.0118999	−3.7138729
H	1.14871345	−0.5895992	−2.0911405	H	5.86247162	−2.9314399	−2.1031475
C	3.45037353	−4.1384823	−1.7026379	H	5.33214171	−1.6462583	−3.2042892
O	3.79529682	−4.7607356	−2.7145237	H	6.27965848	−1.253235	−1.7640054
C	3.43420776	−4.8070295	−0.3530649	H	0.79069548	−2.9405815	−2.6433195
O	2.78614544	−2.381274	−3.2085155				

conformer 1a_11							
C	−1.4922248	2.77199864	2.27820783	C	4.4772883	−1.5080665	−3.1807603
C	0.71459898	2.0343681	0.58669601	H	−1.5235928	0.65195307	2.24716657
C	0.4412287	3.52806436	0.85943629	O	−0.0691651	−2.6752068	−1.5214709
C	−0.6128153	3.73833525	1.92845319	H	0.13787687	4.04645646	−0.0571681
C	−0.266095	−0.2512246	0.10061385	H	−1.1731413	−0.7785576	−0.2000403
C	0.79739105	−0.4777275	−1.0022222	H	1.39689229	2.2620048	−1.4781434
C	2.07862925	0.32930159	−0.7301922	H	1.48032001	−2.3833163	−0.2169794
C	1.75307487	1.82393631	−0.5404845	H	3.9494556	−1.7197827	−1.1230636
C	1.13325816	−1.9764642	−1.1726228	H	4.02904666	0.6539186	−1.6294004
C	2.18818256	−2.2294001	−2.2718608	H	2.71717213	0.52634615	−2.7949582
C	3.45575633	−1.3782957	−2.0425065	H	−3.5266627	2.37397492	0.96815298
C	3.10490571	0.10915505	−1.8563756	H	−3.3870818	1.83626439	−1.2786273
C	−0.5860608	1.24773531	0.34854248	H	−4.2771943	0.55122083	2.31073349
C	−1.6208461	1.45264471	1.50700542	H	−4.9416135	0.46087004	0.70144447
C	−3.0079641	1.41941345	0.80171493	H	−4.0885402	−1.2849532	−0.9365694
N	−2.7327112	1.44391001	−0.6111382	H	−3.3718741	−3.6405795	−1.1021855
C	−1.4009974	1.70989251	−0.8476228	H	−2.4651131	−4.8150309	0.88043739
O	−0.9797238	2.17982831	−1.8902447	H	−2.2802508	−3.6244184	3.05106161
C	−4.0063739	0.34508897	1.26655764	H	−2.9790455	−1.2670674	3.23641192
C	−3.5773943	−1.0956062	1.15957099	H	−2.5280029	1.93057023	3.96926838
C	−3.6948475	−1.7850981	−0.0544463	H	−2.0520559	3.59329729	4.21945532
C	−3.2924973	−3.1178906	−0.1523076	H	−3.4173584	3.22608612	3.14379528
C	−2.7789153	−3.777661	0.96239186	H	0.20031475	5.19023109	3.2988285
C	−2.6716765	−3.1075574	2.17852291	H	−0.4434809	5.87903613	1.79011371
C	−3.0696668	−1.7735033	2.27758833	H	−1.5473772	5.36560624	3.04404308
C	−2.4242723	2.8965405	3.46282108	H	2.38464304	3.70938552	0.88565748
C	−0.6011388	5.1127039	2.55748588	H	3.47747334	2.62386355	−1.0122355
O	1.63546804	4.15905714	1.33033891	H	−0.2408618	−1.719892	1.37062102
H	1.18696921	1.64372961	1.50318126	H	1.94200093	−4.6973326	−0.5478175
O	2.95530105	2.52490472	−0.19471	H	3.33623376	−5.4404684	−1.3605703

O	0.1942826	-0.8459085	1.31794597	H	3.55787864	-3.9232654	-0.4785247
H	2.54530709	-0.0343874	0.19694735	H	1.94459198	-2.4775163	-4.1958932
H	0.3565176	-0.1252643	-1.9449443	H	4.06449455	-1.1691293	-4.1365594
C	2.54229475	-3.730888	-2.3608489	H	5.36633754	-0.9035758	-2.9696593
O	2.57741515	-4.2891922	-3.4640901	H	4.81124104	-2.5432486	-3.3003094
C	2.8614829	-4.4900405	-1.0996377	H	-0.24776	-2.4377713	-2.4557835
O	1.58106797	-1.8564055	-3.5282386				

conformer **1a**_12							
C	-0.9464318	3.47298015	1.437247	C	4.22226573	-3.1210503	-2.2289213
C	1.1422416	1.90123112	0.26070658	H	-1.501773	1.46250388	1.82792339
C	1.21412361	3.43792734	0.15424199	O	-0.5974333	-2.8625878	-0.9992383
C	0.16861002	4.11511551	1.01843501	H	1.10225486	3.77574361	-0.8822695
C	-0.321008	-0.1638056	0.10356044	H	-1.302618	-0.5331251	-0.1973342
C	0.73937303	-0.8593546	-0.7840058	H	2.09728372	1.49875573	-1.6593626
C	2.15047918	-0.3304496	-0.4877346	H	0.85511351	-2.6570119	0.42708511
C	2.20802505	1.2030026	-0.611319	H	3.4913522	-2.7752436	-0.2533938
C	0.70574779	-2.3960719	-0.6259353	H	4.18834799	-0.6291222	-1.1723787
C	1.75805995	-3.1079024	-1.5064522	H	2.98614766	-0.7123085	-2.4535171
C	3.17351466	-2.5318615	-1.2758645	H	-2.8945935	3.24932287	-0.0394055
C	3.1834901	-0.998568	-1.4120114	H	-2.6941258	2.1927944	-2.0897471
C	-0.2831305	1.38451692	-0.0003802	H	-4.1942276	2.00213177	1.51954501
C	-1.3330347	2.07451533	0.93606605	H	-4.7096276	1.6826339	-0.1151858
C	-2.6169659	2.19095997	0.06440823	H	-4.1613072	-0.5441059	-1.2137908
N	-2.2200557	1.82793527	-1.2711179	H	-4.028921	-2.9697477	-0.7813192
C	-0.8481827	1.73144476	-1.3668053	H	-3.6265762	-3.8120804	1.51231218
O	-0.2308455	1.8503738	-2.4104031	H	-3.360573	-2.2129128	3.39266809
C	-3.8876662	1.50172816	0.59121847	H	-3.4766949	0.21571291	2.98147455
C	-3.8170664	0.01858815	0.84932106	H	-2.2879862	3.2881396	3.11222666
C	-3.9860353	-0.8951852	-0.1991679	H	-1.4463935	4.81933128	3.07863174
C	-3.9137192	-2.2680961	0.0410996	H	-2.7680211	4.52144883	1.9294337
C	-3.6830426	-2.7418817	1.33102286	H	1.21219371	5.6184253	2.15652584
C	-3.5295125	-1.8437701	2.38426749	H	0.86417577	6.07652963	0.473105
C	-3.5970702	-0.4703174	2.14551031	H	-0.4172032	6.11761091	1.66106198
C	-1.9131883	4.06588717	2.4377056	H	3.15719952	3.38276302	0.09513612
C	0.47033035	5.55728099	1.35414368	H	3.6612096	1.33062518	0.70218101
O	2.49245729	3.88145405	0.61434474	H	-0.7595433	-1.2604274	1.64401063
H	1.40674927	1.65674092	1.30277554	H	2.50365828	-4.6315872	0.74651098
O	3.51308277	1.64463425	-0.2102848	H	0.76172502	-5.0307968	0.60032516
O	-0.1099479	-0.5512671	1.46552282	H	2.00285529	-6.2177142	0.14463062
H	2.42595144	-0.599081	0.54263321	H	1.65032121	-3.6918254	-3.3696442
H	0.48657916	-0.6260855	-1.8272762	H	3.98606469	-2.904176	-3.275813
C	1.74090048	-4.6335974	-1.2610871	H	5.21186618	-2.7009528	-2.0177371
O	1.73773773	-5.412043	-2.2222403	H	4.30038862	-4.2064011	-2.1136318
C	1.75047152	-5.1532734	0.15275071	H	-0.6235432	-2.8039866	-1.9779684
O	1.3657164	-2.8910148	-2.8790255				

Table S2. Cartesian coordinates for the low-energy optimized conformers of **2a** at B3LYP-D3(BJ)/6-311G* level.

conformer **2a**_1							
C	-1.4074312	3.03848905	-0.2130818	C	5.28167855	-3.1059743	1.03687339
C	1.05063952	1.63279923	0.40496802	H	-1.9511117	1.11430043	0.49958741
C	1.08520723	3.13854853	0.09218329	O	0.52106133	-3.4744386	-0.4193819
C	-0.2754607	3.76837052	-0.0825725	H	1.55489928	3.63225398	0.9532817
C	0.02868112	-0.6443983	-0.0830858	H	-0.5625655	-1.2088322	-0.81039
C	1.44379264	-1.2728435	-0.0594896	H	2.9049345	1.07797843	-0.6264265
C	2.42772662	-0.4684483	0.80198773	H	1.01666625	-2.7828425	1.44023911
C	2.4569001	1.00578479	0.37051028	H	3.59286754	-2.5876301	2.2347179
C	1.41963481	-2.7406046	0.42266379	H	4.52588219	-0.522275	1.36330618
C	2.81590827	-3.4007076	0.38559554	H	4.22003518	-1.0034342	-0.3001702
C	3.85238285	-2.5629388	1.16811994	H	-2.5999456	1.93422222	-2.1758343
C	3.83639382	-1.0890345	0.72523655	H	-0.9914183	1.01059967	-3.5830923
C	0.02439013	0.86535812	-0.4507234	H	-3.2796814	-0.4603404	-2.6291704
C	-1.4042944	1.50659756	-0.3643589	H	-2.6236473	-0.9439377	-1.0942545
C	-2.0783888	1.09149491	-1.7083752	H	-3.875398	-0.5956744	0.95538832
N	-0.964394	0.81161427	-2.5890808	H	-6.0010996	0.05354688	2.02331194
C	0.24408574	0.96206519	-1.9475571	H	-7.7152802	1.30419412	0.73999965
O	1.30454646	1.14786336	-2.519253	H	-7.2976637	1.9025438	-1.6299819
C	-3.0606935	-0.0891643	-1.6185109	H	-5.1790225	1.26045111	-2.7197749
C	-4.3683799	0.28498939	-0.9627332	H	-3.4908132	2.98537026	0.34133887
C	-4.6170697	-0.0508335	0.37486237	H	-2.8044848	4.58455995	0.43011734
C	-5.8174665	0.31625152	0.98459926	H	-3.1595821	3.87737524	-1.1597925
C	-6.7803928	1.01985764	0.26410598	H	-0.0490926	5.61374914	0.99681996
C	-6.5449235	1.35633027	-1.0672929	H	0.54437888	5.67111968	-0.6751146
C	-5.3450212	0.99104318	-1.6790039	H	-1.1747783	5.74110793	-0.3690828
C	-2.786294	3.6604501	-0.1549026	H	1.67893288	2.87021752	-1.7705703
C	-0.2458682	5.27819005	-0.0264058	H	3.58937527	2.52768514	0.80464706
O	1.90485288	3.45953621	-1.0246937	H	-0.9272001	-1.7366473	1.21878671
H	0.69952644	1.56550524	1.4477536	H	0.97598118	-5.0798315	2.08842181
O	3.31573703	1.71252438	1.26785671	H	2.32902133	-6.1171734	2.58763659
O	-0.6010829	-0.8169293	1.18930665	H	2.36573665	-4.3953652	2.99078941
H	2.11212189	-0.5120794	1.85457059	H	3.7613461	-4.2789328	-1.0829055
H	1.80102175	-1.2608369	-1.099283	H	5.97877118	-2.4979143	1.62397947
C	2.75938919	-4.8467157	0.92787842	H	5.35378327	-4.1321615	1.40991306
O	3.3011487	-5.762105	0.29694584	H	5.62555829	-3.0948204	-0.0025707
C	2.05786652	-5.1183979	2.23301395	H	1.02003095	-3.6408511	-1.2483024
O	3.19705171	-3.4798248	-1.0040949				
conformer **2a**_2							
C	-0.413407	3.40907269	1.12882861	C	4.41870665	-3.8819378	-1.6144665
C	1.62006519	1.55570469	0.21705901	H	-0.805313	1.55231268	2.08168232
C	1.7500718	3.05480732	-0.1010715	O	-0.2548358	-3.2912649	0.03528147
C	0.6966065	3.92010767	0.54816043	H	2.71983764	3.38250304	0.29628979
C	0.09836393	-0.4586407	0.51016858	H	-0.9026041	-0.8488987	0.30394741

C	1.10364166	-1.322988	-0.2897064	H	2.37181712	0.78071214	-1.6921796
C	2.53713037	-0.778445	-0.207299	H	1.35423202	-2.859985	1.22228996
C	2.58844012	0.70064526	-0.6214916	H	3.89021462	-3.1810671	0.33105621
C	1.08628121	-2.8002437	0.16202961	H	4.50672657	-1.243377	-0.9971813
C	2.03932394	-3.6870014	-0.6700857	H	3.19720712	-1.5209665	-2.1375596
C	3.47367448	-3.1116416	-0.6824836	H	-2.6969188	2.71694334	0.2733207
C	3.48171606	-1.6253255	-1.0820505	H	-2.4646949	1.59652854	-1.7636774
C	0.16475631	1.05324964	0.15859049	H	-2.7172803	-0.2269457	1.15927277
C	-0.789561	1.92088527	1.04879656	H	-3.2116727	1.10931864	2.1681844
C	-2.1835294	1.75358433	0.37387356	H	-5.2459983	2.45600277	1.61452074
N	-1.8757026	1.3438275	-0.977237	H	-7.4881687	2.39254339	0.58522292
C	-0.5205222	1.23011135	-1.1828491	H	-8.0403132	0.6613242	-1.1020327
O	0.01676065	1.26900435	-2.276129	H	-6.3362173	-1.0155036	-1.7617916
C	-3.11935	0.78972323	1.12190232	H	-4.0868528	-0.9684939	-0.7459308
C	-4.5025824	0.74408959	0.51346715	H	-1.8573016	3.6472537	2.71606124
C	-5.4714831	1.68940599	0.87674142	H	-0.8359241	5.04815458	2.49521162
C	-6.7405749	1.65801889	0.29691886	H	-2.1271593	4.70666595	1.31771686
C	-7.0514851	0.68537723	-0.6515023	H	1.88806229	5.58680115	1.19914227
C	-6.0940864	-0.2570267	-1.0217171	H	1.31389507	5.71136352	-0.4764293
C	-4.8245921	-0.2281204	-0.443303	H	0.21036192	6.03266012	0.84296775
C	-1.3590842	4.25486234	1.95256379	H	1.07047319	2.85273907	-1.9431857
C	1.04016863	5.39158957	0.53471352	H	4.02617339	1.95822584	-0.9936584
O	1.79644809	3.32926269	-1.4953454	H	-0.0932995	-1.4610196	2.17275036
H	1.94162791	1.45892724	1.26706355	H	2.43211703	-6.4539355	1.48719921
O	3.92510613	1.16810065	-0.4285261	H	3.02194545	-4.8042549	1.73034517
O	0.32753985	-0.6192945	1.91270315	H	1.26888736	-5.1606574	1.84843582
H	2.89872355	-0.8573653	0.82831971	H	1.75713706	-4.5881099	-2.3819246
H	0.77559587	-1.283555	-1.3383473	H	5.4275629	-3.4564482	-1.5752345
C	2.03790084	-5.1418925	-0.1490136	H	4.49895685	-4.932907	-1.3204632
O	1.92066859	-6.0807042	-0.9455141	H	4.08206391	-3.842627	-2.6555939
C	2.19888443	-5.3971823	1.32686451	H	-0.3748542	-3.4712744	-0.922333
O	1.50949433	-3.7133094	-2.0123494				
conformer 2a_3							
C	-0.8730751	3.62423182	1.08445327	C	4.07517157	-3.3118313	-2.2623315
C	1.13441094	1.92152009	-0.1011206	H	-1.2716798	1.67933503	1.83855769
C	1.19068093	3.43836352	-0.3366133	O	-0.5715418	-2.9884829	-0.5004088
C	0.18328156	4.2139032	0.47891114	H	2.18477613	3.77563035	-0.0148197
C	-0.2809495	-0.1599593	0.18147069	H	-1.2760713	-0.5819068	0.03233301
C	0.70108711	-0.945529	-0.7230031	H	1.82180503	1.26967107	-2.0804407
C	2.1172293	-0.3498124	-0.6781655	H	1.03030669	-2.5535371	0.69726985
C	2.09283862	1.14819765	-1.0264142	H	3.571293	-2.7068948	-0.2781944
C	0.74785467	-2.4453664	-0.3551957	H	4.06943086	-0.6984233	-1.5623761
C	1.71604685	-3.2500653	-1.2513714	H	2.72708969	-0.9876715	-2.6611799
C	3.12496993	-2.6173361	-1.277386	H	-2.9957811	3.1126104	-0.0477468
C	3.05815094	-1.1195412	-1.6225682	H	-2.9943591	1.82484845	-1.9458089
C	-0.3029551	1.36455367	-0.1138503	H	-4.0083052	2.05419565	1.826644

C	-1.2533587	2.15303987	0.8516618	H	-4.7159352	1.49560015	0.33506112
C	-2.6473387	2.09073512	0.15789628	H	-4.2237776	-0.8444702	-0.5158713
N	-2.4071868	1.56326012	-1.1614708	H	-3.9268322	-3.181653	0.21734312
C	-1.0533565	1.53981921	-1.4184991	H	-3.18016	-3.684399	2.52432645
O	-0.5666799	1.6512443	-2.5307867	H	-2.7302798	-1.8314764	4.11429987
C	-3.8005799	1.43959311	0.94045828	H	-3.0066623	0.51161585	3.39895121
C	-3.6265675	0.00999784	1.38274134	H	-2.1030637	3.66650155	2.85429578
C	-3.8964811	-1.045529	0.50185936	H	-1.2462867	5.16363262	2.5824741
C	-3.7320572	-2.3691263	0.91289429	H	-2.6463175	4.75836935	1.56456133
C	-3.307988	-2.6519137	2.20967971	H	1.38246839	5.82594762	1.24251738
C	-3.05211	-1.6116491	3.09950411	H	0.74555896	6.10145524	-0.3915276
C	-3.2110276	-0.2871318	2.68896734	H	-0.3133849	6.2810603	0.98860957
C	-1.764961	4.34991351	2.06754459	H	0.43164945	3.24214549	-2.1529892
C	0.51227975	5.68363147	0.59379329	H	3.47013229	2.47084336	-1.4151618
O	1.08311708	3.81345737	-1.7030114	H	-0.486976	-1.1064045	1.86377423
H	1.51439621	1.78281416	0.9246445	H	2.2830868	-6.089413	0.76664364
O	3.41894885	1.65966004	-0.8748341	H	2.79200958	-4.4239857	1.07617782
O	0.07342291	-0.3681232	1.55188923	H	1.06239064	-4.8768921	1.21015882
H	2.52575209	-0.4579586	0.3372642	H	1.41451179	-4.0924777	-2.9894798
H	0.31802159	-0.8617243	-1.7498688	H	5.06599527	-2.8451049	-2.2336071
C	1.78716608	-4.7257316	-0.7980875	H	4.20788382	-4.3683443	-2.0102034
O	1.69202322	-5.6321455	-1.6344404	H	3.70846205	-3.2491795	-3.2920963
C	1.99218224	-5.0401402	0.66079791	H	-0.7079006	-3.0816888	-1.4671358
O	1.15410457	-3.2383789	-2.5823646				

conformer 2a_4							
C	-1.8489603	2.73192906	-0.6718042	C	5.54729429	-2.1049351	1.75501758
C	0.74373904	1.81922829	0.2246249	H	-2.1441469	0.84314295	0.25571343
C	0.5806312	3.26030925	-0.2892101	O	1.17258245	-3.3665883	-0.2513719
C	-0.8481255	3.64060491	-0.5967463	H	0.9192869	3.92568868	0.51603475
C	0.11197448	-0.6318874	-0.006598	H	-0.3477454	-1.3690113	-0.6690036
C	1.60653819	-1.0199822	0.1157313	H	2.74702329	1.46169194	-0.5987101
C	2.38166562	0.00291182	0.95755021	H	1.24869697	-2.5388849	1.62324031
C	2.22532881	1.41558884	0.36368723	H	3.65328029	-1.7807927	2.68617679
C	1.78205653	-2.4508575	0.67106414	H	4.3922458	0.33387325	1.70211873
C	3.26537482	-2.8497336	0.8439209	H	4.33680315	-0.33468	0.07663759
C	4.04653116	-1.7971129	1.66110946	H	-2.7490907	1.22598076	-2.522575
C	3.86548075	-0.3890965	1.06681416	H	-0.9262644	0.41397306	-3.7411303
C	-0.1005671	0.80056317	-0.5684317	H	-3.0691301	-1.2417619	-2.7344534
C	-1.6108961	1.21079553	-0.62721	H	-2.3495563	-1.5087205	-1.1777025
C	-2.127911	0.53874611	-1.9372947	H	-3.6588255	-1.3007427	0.82954712
N	-0.9310078	0.35242398	-2.7290414	H	-5.8608314	-0.9242041	1.87800985
C	0.1984628	0.75372384	-2.0549152	H	-7.7311708	0.02483232	0.55346728
O	1.25726639	1.03477675	-2.5900464	H	-7.3943401	0.59205218	-1.8370791
C	-2.914368	-0.7707877	-1.753868	H	-5.2011646	0.22046349	-2.9062213
C	-4.2661084	-0.5617529	-1.1149843	H	-3.9288571	2.42229319	-0.1935155
C	-4.4695489	-0.8836208	0.23383611	H	-3.4964504	4.10801844	-0.2869035

C	-5.7123489	-0.6709804	0.83144131	H	-3.6506805	3.16530134	-1.7842085
C	-6.7628825	-0.1376516	0.08737108	H	-0.9653482	5.61951743	0.23558956
C	-6.572817	0.18139318	-1.2557531	H	-0.2916857	5.55882284	-1.4056394
C	-5.3306961	-0.0300062	-1.8557169	H	-2.0166612	5.39606023	-1.1765519
C	-3.3068299	3.13362294	-0.7464566	H	1.32462385	2.85368034	-2.0730781
C	-1.0499132	5.13152318	-0.7406322	H	3.1138756	3.11288274	0.70997915
O	1.40997522	3.55949377	-1.4024449	H	-0.1823613	-0.1347946	1.89350507
H	0.32990723	1.84180741	1.24557684	H	2.64234644	-3.7556254	3.43857137
O	2.87225921	2.33311425	1.24686102	H	1.53899058	-4.7529036	2.43673838
O	-0.550002	-0.7701307	1.25641993	H	2.97991948	-5.4736771	3.18610465
H	1.98608522	0.01346873	1.98292701	H	4.49949551	-3.6525414	-0.4432472
H	2.01830942	-1.0104967	-0.9037213	H	6.05898265	-1.3422951	2.3524765
C	3.38968487	-4.249136	1.48729511	H	5.72673542	-3.0692241	2.24013598
O	4.16519101	-5.0817044	1.00207714	H	6.01970919	-2.1253919	0.7674867
C	2.57929483	-4.5711225	2.71529177	H	1.80193649	-3.4435857	-0.999377
O	3.82770796	-2.9378801	-0.4845072				
conformer **2a**_5							
C	-1.396596	2.89973667	0.77251883	C	5.2896897	-2.8868631	-1.5316129
C	1.06781438	1.65961865	0.00270393	H	-1.6573913	0.85574487	1.23392733
C	0.89367449	3.17670585	-0.208366	O	0.37579452	-3.43492	-0.8344515
C	-0.3419197	3.69769551	0.48735585	H	1.77072604	3.69262521	0.20133865
C	0.00696206	-0.6451373	-0.1404512	H	-0.8425692	-1.2054638	-0.545218
C	1.30643122	-1.2076375	-0.7672957	H	2.3531929	1.27419045	-1.7226624
C	2.54354967	-0.393056	-0.355548	H	1.60113847	-2.8032142	0.67462194
C	2.35154344	1.10820495	-0.6417864	H	4.26534341	-2.5125082	0.30298727
C	1.52820139	-2.6950713	-0.4128448	H	4.67846707	-0.3618191	-0.7495555
C	2.79150885	-3.2806613	-1.0839067	H	3.70056712	-0.7808071	-2.1501174
C	4.04091971	-2.425209	-0.7683392	H	-3.2230348	2.13176788	-0.8300557
C	3.79778001	-0.9339069	-1.067109	H	-2.3503722	1.27186652	-2.8718175
C	-0.1921835	0.87338428	-0.4004145	H	-4.1729842	-0.1703152	-1.28727
C	-1.4705128	1.43180481	0.32089072	H	-2.976179	-0.8811162	-0.2457324
C	-2.6177549	1.22590493	-0.7096058	H	-3.3756	-1.0026827	2.12865328
N	-1.943349	1.04475144	-1.9720218	H	-4.8712766	-0.5776097	4.04301932
C	-0.5750517	1.01827133	-1.8648856	H	-6.8168484	0.94017151	3.79727117
O	0.17936783	1.00928116	-2.8239057	H	-7.2688342	2.03269265	1.61732084
C	-3.5491827	0.03838711	-0.4069846	H	-5.7873975	1.61872737	-0.3117317
C	-4.4682342	0.27953432	0.76583665	H	-2.8989077	2.57232802	2.28022188
C	-4.2256955	-0.3362346	2.00102175	H	-2.3568478	4.22718135	2.21588439
C	-5.0680607	-0.097554	3.0878867	H	-3.4260181	3.61484118	0.94070353
C	-6.1610336	0.75563638	2.95046088	H	0.40101769	5.33734774	1.65717355
C	-6.4143801	1.3695854	1.72572388	H	0.11175522	5.73095231	-0.0490089
C	-5.5733559	1.13250413	0.63768265	H	-1.2443032	5.60773899	1.04779588
C	-2.5829417	3.36046944	1.58911658	H	1.54915565	3.22930327	-2.0484358
C	-0.2718704	5.16972746	0.81023203	H	3.5505607	1.63687194	0.82419132
O	0.76756792	3.55416859	-1.5694023	H	-0.2835975	-1.794873	1.40662171
H	1.19613257	1.53218466	1.09035157	H	3.36180182	-6.1138402	0.94419916

O	3.4773828	1.82602402	-0.1278197	H	3.50988092	-4.4116633	1.4020048
O	0.03396696	-0.8813342	1.27256286	H	1.89199308	-5.1321265	1.12252872
H	2.71078002	-0.5143109	0.72472366	H	3.03582122	-4.0468304	-2.8666869
H	1.1863006	-1.1387555	-1.8566698	H	6.15432635	-2.2705306	-1.2615913
C	3.01381361	-4.7501723	-0.6600622	H	5.54338506	-3.9236484	-1.2905881
O	3.27118525	-5.605591	-1.5151961	H	5.15329957	-2.8109651	-2.6153317
C	2.93499826	-5.1170759	0.79883651	H	0.46191622	-3.5161081	-1.8091849
O	2.54457017	-3.2783199	-2.5047022				
conformer 2a_6							
C	-1.5940552	2.92325935	-0.6615897	C	5.21672433	-2.5381673	2.12681633
C	0.84400231	1.78430301	0.39985466	H	-2.1034453	1.0418013	0.17783606
C	0.829723	3.24833537	-0.0748466	O	0.83329231	-3.4297273	-0.0114193
C	-0.5368818	3.74822064	-0.4773997	H	1.15414236	3.86238782	0.77574933
C	0.04752853	-0.6034453	0.03630525	H	-0.3785843	-1.2776539	-0.7113574
C	1.47923306	-1.1192457	0.31304595	H	2.86328772	1.28068962	-0.2987395
C	2.27933619	-0.1603623	1.20502017	H	0.90206465	-2.5567867	1.85884689
C	2.27627501	1.26480884	0.62601862	H	3.34004735	-2.0194338	3.00508786
C	1.4747573	-2.5403286	0.92315627	H	4.27924876	0.00509623	2.03677428
C	2.90557383	-3.0835383	1.16797826	H	4.23615705	-0.6704479	0.41352608
C	3.75617461	-2.0873903	1.99022133	H	-2.4558239	1.5421562	-2.6254849
C	3.71996506	-0.675358	1.38268191	H	-0.6080425	0.62689202	-3.7235056
C	-0.0094781	0.85769496	-0.4866953	H	-2.9336413	-0.8967952	-2.9317619
C	-1.4750345	1.38864352	-0.6490574	H	-2.3744421	-1.2423157	-1.3248971
C	-1.9369916	0.79431252	-2.0154135	H	-3.8316459	-0.9476303	0.57533481
N	-0.6989939	0.53234175	-2.7177128	H	-6.0836815	-0.4233965	1.43459228
C	0.40178624	0.82599112	-1.9466607	H	-7.7607271	0.66497487	-0.0329123
O	1.51835623	1.02997959	-2.390625	H	-7.1792514	1.22453075	-2.3776155
C	-2.8311237	-0.4548255	-1.9310307	H	-4.9337289	0.70617739	-3.2596697
C	-4.2142427	-0.155628	-1.4059811	H	-3.7236175	2.76316554	-0.360364
C	-4.5556889	-0.4715759	-0.0838763	H	-3.1586994	4.41190335	-0.3582938
C	-5.827555	-0.1752825	0.40766628	H	-3.2612762	3.5291593	-1.8961879
C	-6.7697195	0.43665765	-0.4165725	H	-0.5689427	5.70526148	0.4124118
C	-6.4421332	0.75134816	-1.7338755	H	0.22584802	5.63973534	-1.173508
C	-5.1706985	0.45653273	-2.2277973	H	-1.5192025	5.60617333	-1.0830307
C	-3.0065238	3.43983168	-0.8363182	H	1.68119323	2.83297018	-1.8092516
C	-0.6128298	5.25401829	-0.5839613	H	3.27714986	2.87879575	1.05787921
O	1.7624213	3.51346256	-1.1128995	H	-0.4122257	-0.206835	1.92131575
H	0.35765744	1.80518672	1.3886134	H	3.10575202	-6.5091312	2.00868144
O	2.9321626	2.1181379	1.56470639	H	2.68213597	-5.8651684	0.40888208
O	-0.768442	-0.7572546	1.20043465	H	4.31591949	-5.5838352	1.09842424
H	1.82633301	-0.1258228	2.20615937	H	2.79998958	-3.6269509	-0.6871565
H	1.97841614	-1.1745767	-0.6654081	H	5.78128387	-1.8295024	2.74267113
C	2.75804591	-4.4002095	1.96703668	H	5.28666659	-3.5175522	2.61017871
O	2.25134299	-4.4232753	3.09258721	H	5.71375717	-2.6021996	1.15324199
C	3.24987075	-5.6702509	1.32176283	H	-0.1237616	-3.3813182	0.18071877
O	3.51751002	-3.2917192	-0.1093414				

conformer 2a_7							
C	-2.2063807	2.86133879	0.16651516	C	5.55494117	-1.717382	1.84208642
C	0.48576463	2.04001676	0.83653572	H	-2.358884	0.92728957	1.03689229
C	0.22015042	3.48853118	0.39154616	O	1.0875048	-3.1033452	0.14663817
C	-1.2439189	3.81228985	0.20806205	H	0.59224942	4.14137717	1.19220304
C	-0.0604473	-0.4264402	0.57116031	H	-0.5455309	-1.1632336	-0.0737464
C	1.45354464	-0.7538071	0.5616956	H	2.43273796	1.80186787	-0.1498502
C	2.25223509	0.27521816	1.37400979	H	1.27867525	-2.3337463	2.03812588
C	1.98968555	1.69901459	0.84689966	H	3.73163434	-1.5035442	2.93253394
C	1.73077319	-2.1924085	1.05098035	H	4.30211843	0.67012275	1.96406422
C	3.23829908	-2.5320007	1.09242995	H	4.14030309	0.04945081	0.32669392
C	4.04029125	-1.4717685	1.87925402	H	-3.1632192	1.39335849	-1.6701717
C	3.75464763	-0.0552584	1.34935411	H	-1.4219277	0.62761003	-3.0377953
C	-0.3723727	1.011269	0.07300828	H	-2.5834557	-1.3806792	-0.477357
C	-1.898321	1.35493931	0.13800798	H	-4.0208304	-0.4442457	-0.1548209
C	-2.4819069	0.70731307	-1.1536192	H	-5.6652109	0.05793694	-1.99223
N	-1.3388841	0.57690652	-2.0280349	H	-6.6813466	-0.8079537	-4.0684414
C	-0.1812369	1.01623538	-1.4322947	H	-5.4902785	-2.5046795	-5.4293532
O	0.82321733	1.35662638	-2.0331262	H	-3.2692622	-3.3388042	-4.705333
C	-3.2335986	-0.6104599	-0.9018071	H	-2.2349979	-2.4840604	-2.6328393
C	-3.875815	-1.1522391	-2.158972	H	-4.2590879	2.3882647	0.64036688
C	-5.1292755	-0.6854583	-2.5776384	H	-3.8906533	4.0905062	0.78679726
C	-5.7075188	-1.1717575	-3.7511024	H	-4.0543226	3.36176526	-0.8296976
C	-5.0386493	-2.1252724	-4.5165007	H	-1.3415276	5.76064569	1.11164354
C	-3.7910115	-2.5941146	-4.1097116	H	-0.866446	5.7733305	-0.5992125
C	-3.2107564	-2.1097428	-2.9368744	H	-2.5450494	5.53071816	-0.168608
C	-3.6808132	3.20031867	0.18593804	H	0.83518116	3.1767578	-1.4594941
C	-1.5222176	5.29616314	0.13691307	H	2.83269056	3.42045969	1.18697291
O	0.94459873	3.86201486	-0.7711664	H	-0.1953447	-0.0153463	2.51022289
H	0.1521431	2.00908049	1.8862329	H	2.863156	-3.5406551	3.69643701
O	2.6633361	2.61426149	1.71239658	H	1.724742	-4.555281	2.7529562
O	-0.6134509	-0.623302	1.87781352	H	3.24984845	-5.2341266	3.36178269
H	1.94291983	0.235124	2.42785327	H	4.39678706	-3.2412741	-0.3145733
H	1.78164723	-0.6953976	-0.4860366	H	6.08230877	-0.9520978	2.42228164
C	3.47057326	-3.9434293	1.67692878	H	5.81218982	-2.6874797	2.27811633
O	4.23719455	-4.7271252	1.1041281	H	5.94578209	-1.6871038	0.81976764
C	2.77572565	-4.3365003	2.95405451	H	1.6599642	-3.1302242	-0.6491351
O	3.69438897	-2.556378	-0.2787574				

conformer 2a_8							
C	-1.8940138	3.35091253	-0.0254715	C	5.07199901	-2.4333239	1.02745401
C	0.67380599	2.12123472	0.19126726	H	-2.3530026	1.34389272	0.45540638
C	0.58954271	3.58904008	-0.2711611	O	0.2806278	-3.0672863	-0.1775612
C	-0.7988721	4.14605204	-0.0384561	H	1.31158044	4.18162121	0.30271481
C	-0.3053569	-0.2134498	0.02086842	H	-0.9580251	-0.8547482	-0.5762906
C	1.12521684	-0.8052547	-0.0303743	H	2.47647278	1.55169078	-0.9130576

C	2.14456623	0.10445563	0.67914942	H	0.81269006	−2.2049112	1.59987686
C	2.09678069	1.53799597	0.11273931	H	3.42068955	−1.8391729	2.24300734
C	1.17522511	−2.2297022	0.56679174	H	4.26986651	0.14421617	1.12357987
C	2.59060809	−2.8461366	0.51669403	H	3.88499392	−0.4999596	−0.4675687
C	3.63479944	−1.9133222	1.1685903	H	−2.7619097	2.54205956	−2.2016567
C	3.56029127	−0.4921603	0.58119842	H	−1.5148152	1.14990428	−3.5915377
C	−0.384249	1.24545626	−0.5015556	H	−4.5719068	1.354444	−1.1532074
C	−1.8228294	1.85178234	−0.3564522	H	−4.2004693	0.67996768	−2.719105
C	−2.5045851	1.58464756	−1.7272017	H	−2.9454058	−1.5411345	−2.8820491
N	−1.4811158	1.04400317	−2.5841714	H	−2.8058535	−3.7934557	−1.8834433
C	−0.2373384	1.16431577	−2.0100184	H	−3.5230961	−4.1709192	0.45681535
O	0.8101264	1.14681343	−2.6374179	H	−4.3994649	−2.2806694	1.80606191
C	−3.8132994	0.77460798	−1.6952665	H	−4.5384987	−0.0196815	0.83124093
C	−3.7425393	−0.6088785	−1.0971243	H	−3.8082962	3.10730896	0.92788455
C	−3.2687201	−1.6887592	−1.8539459	H	−3.2738041	4.76972421	0.89551195
C	−3.1887994	−2.9644913	−1.2936785	H	−3.8629235	4.01144927	−0.5980976
C	−3.5898411	−3.1762504	0.02380118	H	−0.4175543	5.89444676	1.14613165
C	−4.0773858	−2.1140935	0.78134299	H	−0.2033017	6.13348908	−0.5996948
C	−4.1552535	−0.8369992	0.2238189	H	−1.81775	6.07586724	0.07208689
C	−3.2807018	3.84487875	0.31307347	H	1.44483202	3.08897331	−1.9801653
C	−0.8196468	5.64045349	0.16020553	H	3.8721003	2.22518374	0.57742872
O	0.86653274	3.79908645	−1.6501928	H	−1.3378888	−1.0597939	1.43076932
H	0.4246023	2.14427389	1.26554546	H	0.88331832	−4.4367737	2.4341698
O	2.96161135	2.37098128	0.88858467	H	2.29724909	−5.3732913	2.96359564
O	−0.7759374	−0.2619034	1.37230851	H	2.27847141	−3.6219862	3.21179022
H	1.89745139	0.15631772	1.74970989	H	3.46002759	−3.8488805	−0.9189779
H	1.40124954	−0.8783278	−1.0911291	H	5.77658184	−1.75251	1.51779656
C	2.60819101	−4.2406961	1.18198777	H	5.18896473	−3.4139632	1.49870782
O	3.15885582	−5.1880847	0.60810337	H	5.37034292	−2.5204294	−0.0224179
C	1.97076927	−4.4212996	2.53474095	H	0.72575734	−3.2271325	−1.0363293
O	2.91765937	−3.0317156	−0.8777744				

Table S3. Cartesian coordinates for the low-energy optimized conformers of **3a** at B3LYP-D3(BJ)/6-311G* level.

conformer 3a_1							
C	-0.4883692	3.34882583	0.63292012	O	0.23524374	-0.673379	1.24982012
C	1.52922651	1.49322356	-0.2955556	O	-1.4060616	-2.2593382	1.43972072
C	1.71749465	3.00193047	-0.5267873	C	-0.5288957	-1.5651842	1.93530309
C	0.6645335	3.85643367	0.13757998	C	-0.1314413	-1.588759	3.3779379
C	-0.0521654	-0.4821565	-0.1595826	H	2.68035743	3.27545151	-0.0752669
C	0.93148512	-1.3280826	-1.0118582	H	-1.066804	-0.8188966	-0.3894368
C	2.38350533	-0.8540872	-0.8415156	H	2.38678159	0.82537311	-2.2010369
C	2.51790353	0.65280913	-1.1275164	H	1.0224137	-3.0465202	0.34106069
C	0.82488008	-2.8417918	-0.7171938	H	3.60847717	-3.3458004	-0.4721708
C	1.78413672	-3.6888142	-1.5907047	H	4.35747182	-1.331937	-1.6114196
C	3.23819935	-3.1721303	-1.4921898	H	3.07645547	-1.4536731	-2.8100424
C	3.3213577	-1.6609261	-1.7598243	H	-2.7161823	2.83176472	-0.4710243
C	0.06343288	1.04053299	-0.4544516	H	-2.3785231	1.80102761	-2.5364359
C	-0.9258435	1.88991027	0.41837171	H	-4.1659135	0.79582646	-0.5426261
C	-2.2724588	1.83473339	-0.3726646	H	-2.9535719	-0.1348793	0.28356053
N	-1.8814451	1.47167148	-1.7154985	H	-3.1766855	-0.3463728	2.63131691
C	-0.5236754	1.30240296	-1.827334	H	-4.1235987	0.41286963	4.77593587
O	0.08820003	1.37054436	-2.8792027	H	-5.4554403	2.50114134	4.88563065
C	-3.3453666	0.88136368	0.18319827	H	-5.8482789	3.82927139	2.82913052
C	-3.9396768	1.33344165	1.49531278	H	-4.9065734	3.09216216	0.67406288
C	-3.7413076	0.5820738	2.66122667	H	-1.8494053	3.53653138	2.29244426
C	-4.2809608	1.00391917	3.87725222	H	-0.9202752	4.98305171	2.01372542
C	-5.0318032	2.17564659	3.93901441	H	-2.245467	4.57741683	0.90530343
C	-5.2514854	2.92176793	2.78343816	H	1.83922905	5.43252349	1.0089784
C	-4.7129418	2.50102506	1.56650845	H	1.45827493	5.67344337	-0.7077658
C	-1.4267527	4.16157469	1.4990922	H	0.22646658	5.96895735	0.49535923
C	1.06278957	5.31052504	0.24706846	H	1.14741374	2.88667876	-2.4176391
O	1.82428552	3.36087586	-1.897264	H	4.05086204	1.83863562	-1.3312047
H	1.79513145	1.33524108	0.76243096	H	1.83142248	-6.264927	-2.8605082
O	3.85219756	1.04469143	-0.7987511	H	1.24608326	-7.1782198	-1.4559695
H	2.70751412	-1.0379958	0.19308997	H	0.16986768	-5.9992252	-2.2339771
H	0.63822668	-1.1794342	-2.0608031	H	0.35570626	-3.6015877	-2.8931192
C	1.72582819	-5.1359618	-1.0451637	H	5.21152352	-3.5408231	-2.3310994
O	2.08200635	-5.4109127	0.10476511	H	4.19680845	-4.9817664	-2.252597
C	1.20744074	-6.2109999	-1.9652073	H	3.89926292	-3.7550543	-3.4973332
O	1.33480428	-3.6012677	-2.9463772	H	-1.0524763	-3.1248158	-0.2089911
C	4.18549432	-3.9055714	-2.4511862	H	-0.2469239	-0.591337	3.8097188
H	-1.0345582	1.45064632	1.41637527	H	-0.7826492	-2.2791067	3.92148149
O	-0.5178111	-3.2637619	-1.0231678	H	0.90159227	-1.9324403	3.47132833
conformer 3a_2							
C	-0.7585213	3.40764464	1.11686791	O	0.84614387	-0.3686131	1.69990249
C	1.3947689	1.91875202	-0.1241098	O	-0.3887665	-2.2317371	2.20222146
C	1.23001599	3.41839537	-0.4246269	C	0.40016436	-1.3545487	2.52570232

C	0.1611288	4.09506279	0.40088812	C	1.03378218	-1.2274473	3.87555292
C	0.29314457	-0.3151655	0.35958758	H	2.18284211	3.90149723	-0.1709174
C	1.26100357	-1.004861	-0.6397339	H	-0.6577064	-0.8541627	0.32180762
C	2.59279574	-0.2473105	-0.7570057	H	2.00084058	1.30450928	-2.1400478
C	2.36211273	1.2339273	-1.1084144	H	1.93022804	-2.5887017	0.71463025
C	1.51516696	-2.490018	-0.2947244	H	4.3415696	-2.4247057	-0.5507697
C	2.4587943	-3.1863548	-1.3072374	H	4.45612851	-0.3806377	-1.8648618
C	3.77230116	-2.391646	-1.4901961	H	3.03646877	-0.8256717	-2.8023501
C	3.49868212	-0.9136418	-1.8101236	H	-2.9922828	2.38078764	0.51844762
C	0.04792907	1.18256486	0.01398643	H	-2.868108	1.26595525	-1.5325395
C	-0.9073083	1.88156759	1.04116974	H	-2.4825844	-0.5317529	1.35296852
C	-2.336719	1.50250193	0.54726217	H	-3.0105865	0.71953037	2.45291404
N	-2.1463689	1.12444777	-0.8329034	H	-5.2605903	1.81064628	2.16832058
C	-0.8328069	1.23296885	-1.2194633	H	-7.592138	1.441824	1.44898482
O	-0.460449	1.35430078	-2.3732164	H	-8.1354808	-0.3787443	-0.1442045
C	-3.0205982	0.41930871	1.39707529	H	-6.3325345	-1.8385545	-1.021221
C	-4.4549682	0.18624345	0.98109524	H	-3.9936484	-1.4863619	-0.3163168
C	-5.4806834	1.00742906	1.4690291	H	-1.9789644	3.39958237	2.89780618
C	-6.8007867	0.80330277	1.06475574	H	-1.236619	4.94878005	2.5764418
C	-7.1069587	-0.2196373	0.16919996	H	-2.6124696	4.4093787	1.58294049
C	-6.0939494	-1.0399725	-0.3233219	H	1.1553537	5.92657553	0.92927058
C	-4.7732979	-0.8382801	0.07928407	H	0.33328519	5.97118299	-0.6439313
C	-1.6989227	4.08453303	2.08986619	H	-0.6053667	6.09983047	0.82714301
C	0.26210946	5.60315604	0.38520081	H	0.34244458	3.08798908	-2.1607126
O	1.01360208	3.69952818	-1.8003247	H	3.55549917	2.67757846	-1.6438354
H	1.87619377	1.88150138	0.86683327	H	1.25456121	-5.799888	-1.5794698
O	3.62502723	1.90020868	-1.0569588	H	2.7983254	-5.7796011	-2.4961511
H	3.12481613	-0.2949804	0.20414697	H	2.66555481	-6.6986552	-0.9837922
H	0.76172574	-0.980005	-1.6188978	H	0.83735166	-3.4574813	-2.3278346
C	2.79072078	-4.5806093	-0.7232086	H	5.60292589	-2.4216095	-2.6656643
O	3.39313462	-4.7092186	0.34701579	H	4.93116262	-4.02459	-2.3635072
C	2.34433759	-5.7907863	-1.50237	H	4.17916864	-2.9607859	-3.5657952
O	1.77187618	-3.2718066	-2.5595183	H	-0.1512677	-3.0999228	0.55151891
C	4.66734303	-2.9859959	-2.5859673	H	0.81273285	-0.2436541	4.29731847
H	-0.7313223	1.50542675	2.05621356	H	0.62177484	-1.9902647	4.54221566
O	0.25279033	-3.1824376	-0.3419627	H	2.11294468	-1.3776012	3.79262121
conformer 3a_3							
C	-0.4456091	3.35257019	0.64870403	O	0.48555043	-0.6075552	1.19958494
C	1.38258915	1.53892696	-0.6391311	O	-1.0424732	-2.2219692	1.750711
C	1.45120406	3.04147815	-0.9784713	C	-0.1121883	-1.4852902	2.04902975
C	0.56901835	3.87766587	-0.0777713	C	0.54282557	-1.4349959	3.3936645
C	-0.0710625	-0.4774947	-0.1337153	H	2.48773791	3.36506433	-0.8186122
C	0.7612414	-1.3276203	-1.1300793	H	-1.1001209	-0.8479207	-0.1535576
C	2.2038724	-0.8104761	-1.2503765	H	1.94194702	0.84694099	-2.6338939
C	2.24473862	0.69123766	-1.5932071	H	1.15859241	-2.9941617	0.23428964
C	0.75846653	-2.831991	-0.7729882	H	3.55128588	-3.2582288	-1.0413927

C	1.56112421	-3.6850717	-1.7867762	H	4.0145736	-1.2717836	-2.3586837
C	2.99163971	-3.1299035	-1.9784115	H	2.5374917	-1.4617524	-3.2943578
C	2.9783745	-1.6272055	-2.302405	H	-2.8261999	2.79809707	-0.0515766
C	-0.0663992	1.03298607	-0.4966932	H	-2.8635944	1.69010479	-2.1188799
C	-0.8968807	1.88828402	0.52305344	H	-4.2431551	0.75248605	0.19406526
C	-2.3613977	1.80645202	-0.0114976	H	-2.8861557	-0.1563932	0.78765655
N	-2.2165723	1.40443396	-1.3916061	H	-2.68378	-0.3518797	3.13574925
C	-0.905446	1.2138853	-1.7450058	H	-3.2204149	0.42553835	5.41192353
O	-0.4997264	1.1890917	-2.8950441	H	-4.509913	2.51641149	5.74783316
C	-3.3016063	0.85534257	0.75120204	H	-5.2745293	3.82855709	3.78789745
C	-3.6435974	1.31978219	2.1461239	H	-4.745159	3.07311292	1.50247554
C	-3.2340219	0.57729659	3.26172717	H	-1.4211904	3.5205729	2.56183127
C	-3.5408621	1.00944257	4.55283415	H	-0.6156215	4.98905776	2.08330594
C	-4.2678641	2.18254054	4.74215331	H	-2.1354595	4.53555916	1.28778353
C	-4.6965718	2.91966185	3.640679	H	1.8857729	5.46458811	0.52801616
C	-4.3910537	2.48856985	2.34885149	H	1.14280282	5.70487407	-1.0657188
C	-1.1965827	4.14860374	1.69348056	H	0.19473211	5.98385856	0.37528546
C	0.96507196	5.335576	-0.0498316	H	0.63105742	2.66021806	-2.7494974
O	1.13928066	3.37598634	-2.3253117	H	4.02672975	1.01445341	-2.3355803
H	1.84758649	1.45063919	0.35672697	H	1.37525367	-6.3088632	-2.9521637
O	3.59831183	1.13546386	-1.4692312	H	1.11364778	-7.1710418	-1.4230636
H	2.71910241	-0.9542897	-0.2894408	H	-0.1166317	-6.0250722	-1.9942111
H	0.27305633	-1.2267801	-2.109378	H	-0.0854657	-3.6754658	-2.8038043
C	1.6488071	-5.1137587	-1.1983065	H	4.7846353	-3.4834078	-3.1591671
O	2.25388047	-5.3456957	-0.1472428	H	3.84192814	-4.9427802	-2.852073
C	0.95662466	-6.2235823	-1.9465062	H	3.28537558	-3.764741	-4.0541196
O	0.86678818	-3.6519348	-3.0370997	H	-0.9817606	-3.1369656	0.08842685
C	3.76459095	-3.8742078	-3.0756544	H	0.02245325	-2.113736	4.07529262
H	-0.8212718	1.46009647	1.52894484	H	1.58518299	-1.7517909	3.30934081
O	-0.6034992	-3.2979508	-0.8056034	H	0.47889167	-0.4217511	3.79849471
conformer 3a_4							
C	-0.7803421	3.37396479	1.2145722	O	0.33520819	-0.5568187	1.82783782
C	1.49985749	1.79481875	0.41644758	O	-1.1158125	-2.3296179	1.8247405
C	1.51904008	3.31927192	0.18794564	C	-0.3834054	-1.5503419	2.4188998
C	0.33877834	4.02463961	0.8196124	C	-0.1266574	-1.5705164	3.89314141
C	0.15952446	-0.3620427	0.40135945	H	2.42721957	3.70776528	0.66670966
C	1.30605996	-1.0656677	-0.373647	H	-0.7843882	-0.8073143	0.07332015
C	2.67120089	-0.4301106	-0.0682889	H	2.62154222	1.29650256	-1.3883084
C	2.65738174	1.09039575	-0.3130556	H	1.47712775	-2.7993802	0.95297947
C	1.35169287	-2.5892523	-0.1152248	H	4.14089185	-2.7726108	0.37544673
C	2.4771251	-3.29545	-0.9119764	H	4.74852277	-0.6594296	-0.6599173
C	3.84581815	-2.6171935	-0.6716576	H	3.60290158	-0.8950542	-1.9732786
C	3.77509641	-1.1001477	-0.9083518	H	-2.8339617	2.6363529	-0.0686263
C	0.12001816	1.16994039	0.13631656	H	-2.25623	1.63699421	-2.1088362
C	-1.0210111	1.8836343	0.93852485	H	-2.806086	-0.3623547	0.60611871
C	-2.2874011	1.69341253	0.0510094	H	-3.5185152	0.86216698	1.63004177

N	-1.7627728	1.38595332	-1.2581159	H	-5.5033072	2.20009165	0.83697664
C	-0.3905372	1.37088839	-1.2761794	H	-7.5699663	2.10878564	-0.5084453
O	0.29190822	1.49119941	-2.2791036	H	-7.8039587	0.44099742	-2.3283026
C	-3.2615924	0.63214681	0.58785358	H	-5.9568084	-1.1445792	-2.8025401
C	-4.5404005	0.56840249	-0.2152961	H	-3.8805066	-1.0695613	-1.4702944
C	-5.5889819	1.4618336	0.0430562	H	-2.3946624	3.33478858	2.64674753
C	-6.7595019	1.41463596	-0.7155013	H	-1.4964441	4.83371754	2.66120924
C	-6.8916462	0.47768492	-1.7386375	H	-2.6208258	4.49465594	1.32299374
C	-5.8538682	-0.4133171	-2.0048377	H	1.30940261	5.69092498	1.76870656
C	-4.6825395	-0.3684546	-1.2479188	H	0.91375322	5.95613519	0.05834798
C	-1.8815584	4.05329329	1.9980545	H	-0.3505744	6.05292264	1.26411568
C	0.55823311	5.5096825	0.9932177	H	1.15641723	3.06155421	-1.7476078
O	1.59813589	3.7163168	-1.1754699	H	4.49642395	1.70592819	-0.5615964
H	1.68913993	1.66691475	1.49511554	H	2.87161908	-5.8263893	-2.2212362
O	3.88369938	1.62532792	0.19167777	H	2.2896314	-6.8235769	-0.8733409
H	2.9210582	-0.6038691	0.98854402	H	1.15516805	-5.7374157	-1.7017866
H	1.09012221	-0.9254665	-1.4421252	H	1.17304099	-3.3341843	-2.3413936
C	2.53986963	-4.7542942	-0.3992058	H	5.91653832	-2.736882	-1.328653
O	2.85553387	-5.0191724	0.76478231	H	5.07180101	-4.2852546	-1.3696659
C	2.18908067	-5.8534723	-1.3684314	H	4.7492866	-3.0664411	-2.6158229
O	2.14624066	-3.2199563	-2.3017625	H	-0.5195803	-3.0952678	0.2110865
C	4.95465472	-3.2121715	-1.5501719	H	-0.7564274	-2.3331214	4.35994038
H	-1.1573925	1.41103741	1.91886148	H	0.92148083	-1.8147881	4.08218787
O	0.100911	-3.1549229	-0.5505322	H	-0.3804941	-0.5998173	4.3266447
conformer 3a_5							
C	-0.8690199	3.22548092	0.68874908	O	0.61689649	-0.4835653	1.13148777
C	1.04182649	1.71194133	-0.8102237	O	-0.6202195	-2.2808153	1.83004129
C	0.83794601	3.20077805	-1.148702	C	0.19750453	-1.3975189	2.0483594
C	0.00788154	3.89504435	-0.0946021	C	0.89631618	-1.1817794	3.35395725
C	-0.0343499	-0.4930134	-0.1672364	H	1.81791627	3.69047597	-1.2038803
C	0.86973428	-1.2402233	-1.1850519	H	-0.9927652	-1.0190669	-0.1092726
C	2.18908237	-0.48786	-1.4318462	H	1.51236807	1.02398416	-2.8292653
C	1.94146685	0.98339856	-1.8243297	H	1.63957501	-2.7384067	0.21300029
C	1.1566741	-2.7017354	-0.7701215	H	3.96392528	-2.6368759	-1.2351176
C	2.03658946	-3.4524808	-1.8025018	H	3.96444213	-0.6720582	-2.6676201
C	3.32589814	-2.6617843	-2.1295339	H	2.48192791	-1.174087	-3.4701996
C	3.0181051	-1.2050336	-2.5124869	H	-3.2065159	2.45796306	0.01793834
C	-0.2962899	0.98712595	-0.5679805	H	-3.2363888	1.27194823	-2.0617485
C	-1.1765627	1.73149241	0.50137184	H	-4.3957566	0.27323749	0.29301653
C	-2.6424308	1.51838688	0.02158961	H	-2.9198403	-0.508034	0.7771992
N	-2.5152842	1.13694416	-1.3621464	H	-2.6018431	-0.7854479	3.09978054
C	-1.2190738	0.97694323	-1.7780201	H	-3.0890103	-0.1492752	5.43067429
O	-0.9027093	0.76682095	-2.937242	H	-4.5330917	1.80707257	5.9173418
C	-3.4394699	0.4559781	0.80267348	H	-5.5029637	3.12458873	4.05395817
C	-3.7477463	0.83544145	2.23051161	H	-5.0254416	2.50933709	1.71538949
C	-3.2216155	0.08719713	3.2923731	H	-1.6653363	3.19011107	2.68930561

C	-3.4993513	0.43917396	4.61402512	H	-1.0995755	4.76850377	2.21463184
C	-4.3131231	1.53614518	4.88797618	H	-2.6201393	4.1537662	1.53887703
C	-4.8573858	2.27630932	3.84081233	H	1.21753317	5.58313	0.45059583
C	-4.5812222	1.92514153	2.51851236	H	0.27060811	5.81278021	-1.0329722
C	-1.6056411	3.87565769	1.83754418	H	-0.523145	5.92404378	0.52131233
C	0.25252314	5.38246426	-0.0255126	H	0.71230971	3.01815841	-3.0878084
O	0.17395609	3.41655654	-2.3818272	H	3.69853219	1.49130202	-1.089939
H	1.59608801	1.70812871	0.14275366	H	0.80731966	-6.0627876	-1.763921
O	3.19450482	1.66834291	-1.9032183	H	2.25841274	-6.1364093	-2.8187821
H	2.78555451	-0.4994789	-0.5080371	H	2.26150037	-6.932138	-1.2322814
H	0.31133099	-1.281555	-2.1295861	H	0.35012899	-3.7851681	-2.6939411
C	2.42600089	-4.8024014	-1.1541318	H	5.06759185	-2.7558764	-3.4299547
O	3.13464115	-4.8544115	-0.1439262	H	4.42586617	-4.3402431	-2.9927847
C	1.89952931	-6.0632541	-1.7893563	H	3.58549184	-3.3442622	-4.1946402
O	1.26889886	-3.6237469	-2.9965714	H	-0.4635471	-3.2409178	0.20957872
C	4.14263543	-3.3154551	-3.2524209	H	0.7019425	-0.1683631	3.71436667
H	-1.0249341	1.27712029	1.48682941	H	0.51142986	-1.8911813	4.09200002
O	-0.0978083	-3.4032306	-0.6889144	H	1.96919113	-1.3484748	3.23112162
conformer 3a_6							
C	0.03542248	3.42127249	0.93472667	O	0.37447288	-0.6955741	1.78433588
C	1.85617469	1.28083321	0.21953857	O	-1.8264953	-0.7262652	2.4381781
C	2.24387741	2.74243672	-0.060941	C	-0.6366748	-0.8530415	2.6782109
C	1.27290079	3.75350111	0.49998045	C	-0.0750421	-1.1742621	4.02882512
C	0.04352648	-0.4774669	0.3843875	H	3.20783154	2.91606586	0.43543897
C	0.92524654	-1.4635731	-0.4318454	H	-1.0051832	-0.7212862	0.19252844
C	2.42242786	-1.1802818	-0.2124911	H	2.72156891	0.43453884	-1.6144
C	2.76853696	0.2857505	-0.5307047	H	0.73289901	-3.0918687	0.99124934
C	0.60876237	-2.9354548	-0.0853831	H	3.28082273	-3.7887429	0.32159326
C	1.48355518	-3.9356047	-0.8756969	H	4.34603703	-1.9440027	-0.8664987
C	2.9878362	-3.6166979	-0.722572	H	3.11197766	-1.9542911	-2.1194901
C	3.28287633	-2.1423084	-1.0512684	H	-2.1481416	3.14094798	-0.2846779
C	0.35112487	1.00223936	0.00816273	H	-1.8684781	1.92706989	-2.2652829
C	-0.5683656	2.01896003	0.76284964	H	-2.935955	0.26104635	0.38809844
C	-1.8584647	2.09738806	-0.1134006	H	-3.2072546	1.58719898	1.51009254
N	-1.4426779	1.60359187	-1.4031444	H	-4.7543352	3.46800133	0.90486762
C	-0.119409	1.24908361	-1.4156023	H	-6.8072792	4.06789586	-0.3281968
O	0.5554867	1.17138874	-2.4280546	H	-7.5847658	2.66325059	-2.2182956
C	-3.0735365	1.34450131	0.44857172	H	-6.2996314	0.64578912	-2.8746774
C	-4.3481087	1.70423368	-0.2853945	H	-4.243265	0.02975443	-1.6540444
C	-5.0809775	2.84270617	0.07732611	H	-1.5351923	3.87421844	2.34653558
C	-6.241937	3.18574934	-0.6171264	H	-0.283771	5.09300279	2.2902724
C	-6.6793315	2.39677333	-1.6794952	H	-1.4604874	4.97736682	0.95859343
C	-5.9572584	1.2633927	-2.0483887	H	2.62700015	5.20042728	1.33338125
C	-4.7962088	0.91822619	-1.3556983	H	2.28099818	5.43523017	-0.3922579
C	-0.8569898	4.40079049	1.66600941	H	1.09439328	5.91360136	0.80147723
C	1.84308161	5.15153837	0.57075945	H	1.7491175	2.62888039	-1.9705643

O	2.46997042	3.01543623	-1.4365788	H	4.44829127	1.26111726	-0.6710986
H	2.05495459	1.14248723	1.29467857	H	1.98488197	-5.2953765	1.54392505
O	4.12492854	0.50970575	-0.1375949	H	0.19574094	-5.3674452	1.45080051
H	2.67893862	-1.3675066	0.84047161	H	1.17636991	-6.816232	1.14054939
H	0.68368264	-1.3146785	-1.4928062	H	1.21737233	-4.7048787	-2.6537019
C	1.18558062	-5.3899964	-0.4468572	H	4.93717637	-4.2599509	-1.4439213
O	1.01384517	-6.2620583	-1.3068525	H	3.75703774	-5.5651571	-1.3453576
C	1.1314685	-5.7298978	1.01957011	H	3.66111159	-4.3808992	-2.662027
O	1.10202867	-3.8119374	-2.2628464	H	-0.807663	-3.2707368	-1.3719343
C	3.88089107	-4.5074429	-1.5969024	H	-0.8953243	-1.3670518	4.72607666
H	-0.7995513	1.66552447	1.77240831	H	0.54449574	-2.0728111	3.9685135
O	-0.7682321	-3.1806709	-0.3959582	H	0.50926593	-0.3274649	4.39704619
conformer 3a_7							
C	-0.4921893	3.19867051	0.81474943	O	0.60355208	-0.8158366	1.07723776
C	1.36268672	1.46111694	-0.5690205	O	-1.2309424	-1.8056883	2.04402283
C	1.44971421	2.98256602	-0.7703068	C	-0.0662697	-1.4439868	2.07835598
C	0.52657956	3.7683719	0.12934756	C	0.83667006	-1.6160036	3.26080309
C	-0.0811233	-0.5837499	-0.1843164	H	2.47464456	3.27899613	-0.510539
C	0.71009195	-1.3454368	-1.2852712	H	-1.1028671	-0.9748016	-0.1571519
C	2.15842453	-0.8327087	-1.3807631	H	1.85938081	0.90126622	-2.63406
C	2.19615341	0.68894078	-1.61404	H	1.08856768	-3.0932067	-0.0561575
C	0.69716683	-2.8721531	-1.0543424	H	3.48162214	-3.2961643	-1.3352667
C	1.49753005	-3.6475606	-2.1258507	H	3.96425756	-1.2175032	-2.5214026
C	2.93433014	-3.0978015	-2.2663221	H	2.48578758	-1.3400151	-3.465636
C	2.9275318	-1.575137	-2.4882741	H	-2.8390502	2.68469541	0.04402219
C	-0.0909653	0.95056138	-0.450893	H	-2.9007105	1.64132691	-2.0309916
C	-0.9288623	1.7372552	0.6154216	H	-4.2593135	0.5890147	0.19590404
C	-2.393587	1.68329287	0.06932209	H	-2.9243577	-0.2542264	0.92037038
N	-2.2506103	1.3216393	-1.3211148	H	-2.752839	-0.2116188	3.3111501
C	-0.9331478	1.21555068	-1.6834481	H	-3.462248	0.69400075	5.48930231
O	-0.5311347	1.33911761	-2.8278477	H	-4.938136	2.68483913	5.59694701
C	-3.3591993	0.74233169	0.80737366	H	-5.7138198	3.76479842	3.50334426
C	-3.8064412	1.27928926	2.146666	H	-5.0133826	2.87532581	1.3130068
C	-3.387927	0.67061865	3.33633593	H	-1.51368	3.26958521	2.71122234
C	-3.7909697	1.17739659	4.57295939	H	-0.6957363	4.75846583	2.32778307
C	-4.6216066	2.2940484	4.63353788	H	-2.1974975	4.35042672	1.47497399
C	-5.0564626	2.90038813	3.45737595	H	1.80433863	5.348995	0.83058877
C	-4.6549689	2.39393142	2.22025763	H	1.08831385	5.64662579	-0.7659836
C	-1.2688502	3.9407	1.88170065	H	0.10790757	5.8466789	0.6662152
C	0.89752417	5.23025161	0.22905911	H	0.51789874	2.90531848	-2.512547
O	1.26559606	3.39380827	-2.1173283	H	3.62784946	1.94032959	-2.0383584
H	1.84105648	1.28284175	0.40775799	H	0.995912	-5.4550081	0.24086946
O	3.55955001	1.1106016	-1.5280631	H	2.07575032	-6.6924886	-0.4369367
H	2.68362924	-1.045269	-0.4381839	H	2.72968678	-5.0965031	-0.0433325
H	0.20139168	-1.1547613	-2.2399144	H	0.93461932	-4.3072466	-3.8784383
C	1.51734422	-5.1610118	-1.8141914	H	4.73623628	-3.3525687	-3.4590077

O	1.28115613	-5.9770927	-2.7130381	H	3.81433232	-4.8398298	-3.2497061
C	1.84973915	-5.6222671	-0.419378	H	3.24455967	-3.5950396	-4.3771558
O	0.7919823	-3.4771319	-3.3743831	H	-0.9164179	-3.3280912	-2.0372774
C	3.72089906	-3.7607427	-3.4053265	H	0.30235874	-2.1527829	4.04986361
H	-0.8539817	1.25265379	1.59510163	H	1.71358771	-2.2025222	2.97507673
O	-0.6639907	-3.3158047	-1.0898193	H	1.13537448	-0.6368725	3.64335894
conformer 3a_8							
C	-1.6938991	2.78961699	1.53006034	O	0.0114467	-0.8668127	1.72109234
C	0.96243394	1.84393259	0.99096857	O	-1.0412913	-2.8099062	1.11729966
C	0.69220244	3.35731004	1.01971241	C	-0.6281976	-2.0407699	1.9742273
C	-0.6768797	3.68013622	1.57710221	C	-0.7261222	-2.270499	3.44984365
C	0.13813915	-0.4589756	0.33311185	H	1.45562363	3.83920403	1.64256453
C	1.55587655	-0.8246544	-0.1873766	H	-0.5973202	-0.9863657	-0.2837235
C	2.65257087	-0.0058327	0.51673858	H	2.50336016	1.89289142	-0.5561229
C	2.36721653	1.5105148	0.46097968	H	1.77725965	-2.6810177	0.9529325
C	1.86042189	-2.336857	-0.0842233	H	4.44610971	-2.0776656	1.06981998
C	3.26245394	-2.7008761	-0.6357268	H	4.81033008	0.23658691	0.43289234
C	4.36627163	-1.8263917	0.00298028	H	4.05683183	-0.0015732	-1.1386943
C	4.03022941	-0.328875	-0.0910082	H	-3.1753597	2.22177787	-0.4487016
C	-0.149611	1.0695388	0.26000266	H	-1.9596861	1.66766582	-2.4562815
C	-1.5673587	1.42642176	0.83641115	H	-2.7852164	-0.8317626	-0.427749
C	-2.5252919	1.34149312	-0.3819462	H	-3.9357024	0.00807633	0.58866373
N	-1.6561254	1.40715459	-1.5246128	H	-5.8709303	1.26599846	-0.4317737
C	-0.3188334	1.40576414	-1.2175996	H	-7.5042827	1.26064317	-2.2820962
O	0.55942887	1.53047862	-2.0542675	H	-6.996697	0.06345391	-4.3935361
C	-3.3976858	0.07425872	-0.3659754	H	-4.8405861	-1.1348938	-4.6521302
C	-4.4131168	0.05787173	-1.4854216	H	-3.1929749	-1.1410127	-2.8147561
C	-5.6328584	0.73491844	-1.3503464	H	-3.4623253	2.09221297	2.55029824
C	-6.559318	0.73539916	-2.3941462	H	-2.9637994	3.68722914	3.05726287
C	-6.2747019	0.0629287	-3.5811532	H	-3.7380875	3.47218683	1.47031516
C	-5.0635648	-0.6107437	-3.7262821	H	-0.2287061	5.07695962	3.14396024
C	-4.1357406	-0.613211	-2.6841278	H	-0.3476052	5.80800401	1.53211041
C	-3.0353711	3.03243537	2.18428599	H	-1.806745	5.36678568	2.38894312
C	-0.7780012	5.05223754	2.19733439	H	0.63358543	4.86944597	-0.2110754
O	0.77905738	3.91284405	-0.2825078	H	4.11175291	2.33508148	0.75323835
H	0.9474975	1.52915547	2.04730752	H	3.77563397	-6.1794426	-0.9834674
O	3.32200777	2.17236033	1.29965199	H	2.6514816	-5.1961997	-1.9435609
H	2.69356607	-0.3013835	1.57549065	H	4.42244954	-4.9227695	-2.0570201
H	1.57216943	-0.5736835	-1.2558387	H	2.35111031	-2.7450415	-2.343109
C	3.51375939	-4.1826389	-0.2666833	H	6.50926092	-1.4721399	-0.1235976
O	3.63315151	-4.5477204	0.90702186	H	6.04065806	-3.1228495	-0.5344175
C	3.59460686	-5.1812575	-1.3923755	H	5.7576166	-1.807633	-1.6888215
O	3.25188785	-2.4900059	-2.050741	H	0.09734158	-3.1809596	-0.3205117
C	5.74400785	-2.0734029	-0.6266495	H	-1.2986489	-3.1833325	3.63700241
H	-1.854261	0.68895726	1.59613765	H	0.274591	-2.3888547	3.87240301
O	0.89456287	-3.0486647	-0.8813556	H	-1.2470604	-1.433256	3.92134345

Table S4. Cartesian coordinates for the low-energy optimized conformers of **4a** at B3LYP-D3(BJ)/6-311G* level.

conformer **4a**_1							
C	-0.4174462	1.65888626	2.8225526	O	-1.3940816	1.11000732	-2.6798823
C	0.0690941	-0.937653	1.71579484	C	-1.9872739	0.62721707	-1.7247775
C	0.40638154	-0.6861839	3.19974221	C	-3.4728219	0.6404923	-1.5447052
C	-0.1629787	0.63776895	3.67279018	H	-0.0513332	-1.4859716	3.798564
C	0.06020865	-0.0435489	-0.6542114	H	0.46854396	0.74093906	-1.2997381
C	0.55630632	-1.4158712	-1.1837246	H	1.66482232	-2.3749978	1.31174278
C	0.15085353	-2.569239	-0.2479252	H	-1.0061682	-1.6864251	-2.694902
C	0.57751182	-2.3006999	1.21243416	H	-0.8014177	-4.3613602	-2.1747422
C	0.08771525	-1.701991	-2.62918	H	0.41891674	-4.7223021	-0.1115126
C	0.61533986	-3.0556572	-3.1676525	H	1.83280157	-3.862512	-0.7083959
C	0.28737676	-4.2141425	-2.1976577	H	1.30188766	3.27456707	1.86032001
C	0.73673666	-3.8983152	-0.7612826	H	3.19593662	2.16455712	0.86690803
C	0.50376905	0.23144447	0.81114377	H	1.7009479	4.09544413	-0.4739789
C	-0.0501575	1.60793022	1.33224099	H	0.59888177	2.91180419	-1.1121839
C	1.06875474	2.63707552	0.99995764	H	-1.5978811	3.65567575	-1.5635673
N	2.24243478	1.83182361	0.78415924	H	-3.4361077	5.26335577	-1.2301829
C	2.00645569	0.48135417	0.7883554	H	-3.2285017	7.06818341	0.45784522
O	2.89102433	-0.3516358	0.67772873	H	-1.1597016	7.26682179	1.81056766
C	0.79314134	3.52742713	-0.2271386	H	0.68956618	5.66699832	1.49696711
C	-0.3279602	4.51937628	-0.0348366	H	-1.8010194	3.27114703	2.46589977
C	-1.4946491	4.42920218	-0.8060477	H	-1.7323622	2.7983695	4.14054176
C	-2.536154	5.3404534	-0.6254529	H	-0.3871217	3.72341627	3.44510828
C	-2.4187915	6.35624181	0.32064451	H	-0.5969029	1.69473803	5.53836414
C	-1.2565791	6.46708785	1.08070011	H	-1.2618925	0.05505495	5.42300459
C	-0.2138244	5.55693177	0.90091782	H	0.47040159	0.3221267	5.70373861
C	-1.1146384	2.93104232	3.24835043	H	0.54814097	-4.1166365	1.94012005
C	-0.4075999	0.68725025	5.16080585	H	1.20061891	-2.2986138	-5.8862612
H	-1.0315259	-0.9918102	1.67977305	H	1.50102948	-4.060949	-5.7196269
O	0.00499126	-3.3156669	2.04545167	H	0.10128547	-3.4723461	-6.6385692
H	-0.9437445	-2.6756648	-0.2642125	H	2.19649147	-2.03118	-3.6127089
H	1.65241213	-1.3638326	-1.2123832	H	0.58001051	-5.8262518	-3.6430483
C	-0.099928	-3.3068276	-4.5168772	H	2.0101659	-5.4887394	-2.6514778
O	-1.3165492	-3.5095566	-4.5780296	H	0.62940927	-6.3496274	-1.958757
C	0.73734943	-3.2810794	-5.769553	H	-0.016432	0.08453374	-3.4485629
O	2.03431354	-2.9522095	-3.3170338	H	3.3873198	-1.5627876	4.32729157
C	0.9165101	-5.5420723	-2.6411184	H	1.85082155	-1.6798596	5.20770281
H	-0.9727708	1.86026882	0.79776037	H	2.12377911	-2.6544325	3.72898458
O	0.61200099	-0.6712176	-3.4874145	H	-3.9316413	1.20175639	-2.3635869
O	1.81274536	-0.6219165	3.42269085	H	-3.8564609	-0.3825072	-1.5606105
C	2.3076817	-1.6921483	4.21376122	H	-3.7267366	1.13333675	-0.6027302
O	-1.3917325	-0.0156686	-0.6851862				

conformer **4a**_2

C	0.09666166	3.25313288	1.30584919	O	-0.745675	0.14286092	-3.3132352
C	-1.0689724	0.66390269	1.72186931	C	-1.5614335	0.66533908	-2.5661479
C	-0.7186354	1.53215152	2.94816472	C	-2.7421456	1.46818988	-3.0146251
C	-0.3713825	2.95199165	2.53854671	H	-1.5983136	1.57339336	3.60553673
C	-0.4175047	-0.1172166	-0.6011358	H	0.41912723	-0.1699859	-1.3064246
C	-0.8395081	-1.567394	-0.2394722	H	-0.664336	-1.2799687	2.63310136
C	-1.9094223	-1.5911189	0.86742543	H	-2.161435	-1.8834979	-1.9570718
C	-1.4750144	-0.7707105	2.10267552	H	-3.6666408	-3.4371905	-0.287248
C	-1.31247	-2.3760147	-1.4694967	H	-3.0247593	-3.0583822	2.02008727
C	-1.7009572	-3.8323931	-1.110699	H	-1.3542923	-3.5138159	1.7091775
C	-2.7174448	-3.873792	0.05342925	H	2.46778541	2.92628062	0.46945021
C	-2.2379361	-3.0450148	1.25658214	H	3.34041615	0.71145763	0.76865544
C	0.02892244	0.70438151	0.64382732	H	1.79097083	1.38687032	-2.1092015
C	0.40249964	2.17913801	0.25683522	H	1.73847191	3.13117026	-1.9761912
C	1.92265852	2.13411024	-0.0573743	H	3.84664846	4.4458712	-1.5071154
N	2.37552967	0.89624672	0.51775554	H	6.27105386	4.5524371	-1.9568208
C	1.37644116	0.13984734	1.07541372	H	7.52189037	2.49754196	-2.5586467
O	1.5789628	-0.8854562	1.70430595	H	6.3366288	0.32402504	-2.7101012
C	2.21962114	2.23485872	-1.5635143	H	3.91285238	0.19543438	-2.2627984
C	3.69992403	2.31282529	-1.8581831	H	0.15398025	4.76218539	-0.2342074
C	4.37950321	3.53555934	-1.7716696	H	-0.313406	5.39289725	1.32539469
C	5.75056119	3.60063597	-2.0242216	H	1.38602595	4.95812755	1.02670683
C	6.45419398	2.44615127	-2.3622324	H	-1.6594608	4.08627246	3.82831377
C	5.78819714	1.22512674	-2.4477945	H	-0.1075771	3.63165418	4.56144786
C	4.41739704	1.15735156	-2.1961193	H	-0.1694544	4.94793461	3.41173528
C	0.34744186	4.6691773	0.84013829	H	-2.6369502	-1.5334087	3.47917579
C	-0.5887082	3.96433942	3.63588075	H	-1.4923943	-6.4014166	-2.4014385
H	-1.9703224	1.13113938	1.29188452	H	-2.1911464	-5.8808562	-3.9477211
O	-2.585628	-0.6856846	3.00320336	H	-0.6420381	-5.1921325	-3.4204205
H	-2.8373841	-1.1474603	0.47787197	H	0.19566301	-4.1699723	-1.3015001
H	0.05938524	-2.0701689	0.1404844	H	-3.7707544	-5.3037445	1.31049422
C	-2.3530977	-4.4426649	-2.3745759	H	-3.4077149	-5.9157452	-0.304069
O	-3.4345041	-4.033309	-2.8082011	H	-2.1212094	-5.7998015	0.90997162
C	-1.6149526	-5.5522408	-3.0780309	H	-0.2544421	-1.627892	-2.9517228
O	-0.5120029	-4.5276189	-0.7241377	H	-0.6004774	-0.2719418	4.90762353
C	-3.0160374	-5.3064232	0.51639998	H	1.04043439	0.18590912	5.40172208
H	-0.1589109	2.48142471	-0.6357166	H	-0.2786245	1.36122606	5.57112871
O	-0.2235959	-2.448132	-2.4093951	H	-2.7067396	1.5883586	-4.1010792
O	0.41781008	1.02772219	3.64485438	H	-3.6653147	0.94850973	-2.7469174
C	0.11572441	0.55283107	4.94838823	H	-2.7113806	2.46001027	-2.5563406
O	-1.5408766	0.57456305	-1.2084945				

conformer **4a**_3							
C	0.26776495	2.4131383	2.23569129	O	-0.6179342	0.74514118	-3.0544785
C	-0.4103719	-0.3411895	1.88926408	C	-1.403618	0.85893404	-2.123701
C	-0.0421982	0.15833839	3.30225593	C	-2.7294448	1.54844391	-2.2027405

C	0.0103012	1.67347642	3.33905461	H	-0.8235153	-0.1709153	4.00143994
C	0.0788233	-0.2628736	-0.5985256	H	0.82097411	0.05332154	-1.3388906
C	-0.0719791	-1.8053837	-0.6885078	H	0.36003581	-2.3710101	2.10926342
C	-0.996101	-2.348957	0.41408599	H	-1.5257565	-1.8247014	-2.3248131
C	-0.5672804	-1.8696881	1.81752941	H	-2.5702236	-4.0632659	-1.1308385
C	-0.5603779	-2.2802472	-2.0765165	H	-1.7175606	-4.2653265	1.13673471
C	-0.6819883	-3.8232949	-2.1692764	H	-0.0495244	-4.2953953	0.57922331
C	-1.5301813	-4.3966314	-1.0089102	H	2.56687705	2.81048006	1.20853594
C	-1.0442459	-3.8866152	0.35771952	H	3.80526046	0.77406393	0.84007486
C	0.53809121	0.21605851	0.80903239	H	3.38858824	2.67844113	-1.1532171
C	0.63663189	1.78460025	0.8828338	H	1.90775913	1.92193405	-1.6613024
C	2.11011689	2.10835057	0.50192958	H	0.304127	3.33704378	-2.661238
N	2.80768712	0.86278552	0.68513737	H	-0.6486685	5.5774698	-3.0456945
C	1.99384269	-0.2022829	0.9726303	H	0.24222505	7.54214612	-1.8224536
O	2.4167593	-1.3216289	1.20859202	H	2.10758699	7.25781247	-0.2135405
C	2.31294971	2.63276789	-0.9328236	H	3.07263022	5.02565407	0.18988353
C	1.73730718	4.00591373	-1.1798642	H	-0.2459365	4.27403038	1.28019185
C	0.69588706	4.18286092	-2.1009155	H	-0.4522318	4.32269223	3.00922359
C	0.15681526	5.45016386	-2.3269774	H	1.1866732	4.36172664	2.33045499
C	0.65902146	6.55461393	-1.6424066	H	-1.2982922	2.14508253	4.97457715
C	1.70664034	6.39430647	-0.7382624	H	0.3534551	1.71487542	5.46174366
C	2.24733346	5.12767993	-0.5115912	H	0.03605423	3.30078319	4.79995715
C	0.19108451	3.92288759	2.22080373	H	-2.4362884	-1.9990271	2.43173571
C	-0.2406889	2.24759995	4.71159471	H	-0.2944412	-5.839029	-4.1886409
H	-1.412864	0.07323366	1.69199559	H	-1.2315147	-5.0139746	-5.4501327
O	-1.5647856	-2.2739611	2.76449939	H	0.26728481	-4.2697754	-4.8563295
H	-2.0187709	-1.9888915	0.22932503	H	1.20599742	-3.7387543	-2.5918199
H	0.92973095	-2.2299826	-0.543811	H	-2.1865133	-6.3037605	-0.1932492
C	-1.3906978	-4.1313068	-3.5098539	H	-1.9326243	-6.3317873	-1.9391386
O	-2.5572901	-3.7843909	-3.7202323	H	-0.5412913	-6.3438165	-0.8406001
C	-0.6040934	-4.8619544	-4.5672078	H	0.18568573	-0.9438542	-3.3147978
O	0.63538795	-4.3764542	-2.1128068	H	0.63132697	-2.1411946	4.46237667
C	-1.5432751	-5.9311788	-0.9979954	H	2.21226259	-1.5513096	5.00854415
H	-0.0531362	2.23144601	0.15819882	H	0.73582775	-0.8154609	5.66270602
O	0.40471366	-1.8687661	-3.0624327	H	-2.7728401	2.3522756	-1.4633754
O	1.24119737	-0.2936651	3.72028655	H	-2.8508869	1.98640668	-3.1974963
C	1.18865204	-1.2523065	4.76756069	H	-3.5323297	0.82643807	-2.0353179
O	-1.2056093	0.3585902	-0.8739641				

conformer 4a_4							
C	1.89832661	2.84188585	1.23008936	O	-1.2172701	0.54242292	-2.8882608
C	0.13978037	0.73398901	1.98860776	C	-1.3905054	1.32450944	-1.9643606
C	1.03825406	1.36642598	3.06553093	C	-2.1299353	2.62095959	-2.0742548
C	1.62871518	2.66494839	2.54426196	H	0.429166	1.59626119	3.95034921
C	-0.103296	-0.0015457	-0.4151422	H	0.45313211	-0.2611941	-1.3233191
C	-0.9405693	-1.2334634	0.04334333	H	0.01819939	-1.2915955	2.80016583

C	-1.6354828	-0.9914827	1.39934128	H	-2.6789005	-0.9076626	-1.2489245
C	-0.6512316	-0.4879259	2.48105867	H	-4.1670985	-1.9981563	0.76528164
C	-1.9675861	-1.7064074	-1.0106975	H	-2.872791	-2.088733	2.81112866
C	-2.750211	-2.9654727	-0.5600338	H	-1.611833	-3.0589932	2.0610516
C	-3.3842331	-2.7660887	0.83573801	H	3.87495077	1.45898962	0.62461996
C	-2.3529576	-2.2733653	1.86367296	H	3.88842852	-0.8576696	0.17722055
C	0.90865576	0.45427905	0.68284738	H	3.94957163	2.64445542	-1.4595745
C	1.74609456	1.70122826	0.21590747	H	4.71254946	1.08454532	-1.6343152
C	3.14934693	1.13942509	-0.137229	H	3.25510581	-0.8624893	-2.6864111
N	3.06612735	-0.2819108	0.03757006	H	1.88670994	-1.3206706	-4.6837556
C	1.96923982	-0.6360484	0.7831792	H	0.75453702	0.53342824	-5.876504
O	1.87233638	-1.716957	1.34122468	H	0.99525965	2.86146701	-5.0625265
C	3.73649628	1.56864784	-1.4931348	H	2.35086075	3.34418237	-3.058836
C	2.8967972	1.27454903	-2.7130855	H	1.9406637	4.32009929	-0.3334203
C	2.76279346	-0.0351427	-3.1926514	H	2.14998404	5.0076341	1.2581185
C	1.99360371	-0.2997846	-4.3262228	H	3.48587143	4.1042469	0.51415551
C	1.35896068	0.74245405	-4.9978837	H	0.9345998	4.0936007	3.98634934
C	1.49085158	2.0501645	-4.5362524	H	2.4084399	3.22657945	4.46408727
C	2.2558441	2.31596717	-3.3999646	H	2.50799661	4.52100487	3.29064925
C	2.40018059	4.13854885	0.64430095	H	-1.6705202	-0.8993645	4.10038329
C	1.88328763	3.68794728	3.62073491	H	-4.5890834	-4.3796517	-3.2260656
H	-0.6185915	1.50538693	1.77406871	H	-2.8207868	-4.26527	-3.1251488
O	-1.403352	-0.0884421	3.63285519	H	-3.7317741	-5.2914568	-1.9675875
H	-2.4101832	-0.2218476	1.26734133	H	-1.2129502	-3.9144835	-1.25186
H	-0.2302049	-2.0605261	0.16760805	H	-4.5281975	-3.8591092	2.32940977
C	-3.8735836	-3.1881705	-1.6018147	H	-4.8228868	-4.4030688	0.67681939
O	-4.8443029	-2.4291055	-1.6831637	H	-3.3201655	-4.8516305	1.50310445
C	-3.7378761	-4.360388	-2.5394157	H	-1.1228859	-1.2285388	-2.7154524
O	-1.8357338	-4.0651555	-0.5090209	H	2.08401922	0.81281522	5.45312344
C	-4.0482033	-4.0458676	1.36249913	H	1.15728733	-0.5966223	4.84536278
H	1.26771348	2.13495999	-0.6681592	H	2.93090969	-0.6392189	4.88273796
O	-1.2521878	-2.0640867	-2.2082504	H	-1.4998417	3.43837887	-1.7140113
O	2.14168942	0.52847638	3.40010087	H	-2.372803	2.81127022	-3.1235136
C	2.06208918	0.00337999	4.71721145	H	-3.0575473	2.56737682	-1.4994291
O	-0.9778645	1.12787263	-0.6835586				

conformer 4a_5							
C	-1.5911612	1.28820264	2.8402978	O	-1.5894209	1.0730566	-2.78769
C	-0.6516779	-1.1522809	1.67343342	C	-2.2809561	0.46287705	-1.9843092
C	-0.5702827	-0.9802995	3.20533398	C	-3.7663035	0.30574323	-2.0784836
C	-1.3582203	0.23561862	3.65739123	H	-1.0135053	-1.8673754	3.67892332
C	-0.3832318	-0.0828345	-0.6141794	H	0.03514106	0.78756268	-1.1315525
C	0.34425479	-1.3531677	-1.1330627	H	1.1240875	-2.398023	1.4410361
C	-0.0820584	-2.6079639	-0.3524946	H	-0.9092978	-1.6599309	-2.9009119
C	0.06374419	-2.4180986	1.173003	H	-0.5001887	-4.343367	-2.5006644
C	0.15447755	-1.5759259	-2.6512003	H	0.40391132	-4.7228798	-0.2755796

C	0.90336127	-2.8313863	-3.1682212	H	1.78848873	-3.683898	-0.5876657
C	0.55450469	-4.0852529	-2.3313472	H	0.05760936	3.12701823	2.26033146
C	0.72643089	-3.8297866	-0.8251327	H	2.17505309	2.35485992	1.43536881
C	-0.215297	0.11879907	0.92113519	H	-0.219117	2.98038304	-0.8023845
C	-1.0023494	1.37990101	1.42785068	H	-1.3256182	3.85708637	0.23219369
C	0.01445632	2.54938408	1.32938522	H	-0.5249276	5.83786391	1.58155661
N	1.29477149	1.90702257	1.20524584	H	0.99686478	7.78072948	1.56176328
C	1.23197748	0.53826057	1.1449212	H	3.01619999	7.77180725	0.12254807
O	2.21596934	-0.1817743	1.1459885	H	3.51343593	5.80481198	-1.3041213
C	-0.289778	3.50081143	0.1593552	H	2.00557234	3.8511613	-1.2981651
C	0.62504099	4.7035043	0.13731135	H	-2.9920261	2.85565896	2.36044964
C	0.35488888	5.81826366	0.94275311	H	-3.2285431	2.19252879	3.95848901
C	1.21296036	6.91894562	0.93564847	H	-1.8469416	3.2697053	3.65053957
C	2.34814973	6.91454181	0.12726315	H	-2.1990449	1.0983912	5.48301862
C	2.62721845	5.80966506	-0.674672	H	-2.6134803	-0.5985815	5.18603651
C	1.77077872	4.70807761	-0.6697575	H	-0.9911327	-0.1401428	5.7425175
C	-2.4570245	2.46356428	3.23241182	H	-1.3879283	-3.720665	1.46398494
C	-1.8215785	0.15118682	5.09069926	H	0.98908221	-3.0821084	-6.6915108
H	-1.7222528	-1.3080867	1.46067252	H	1.84369544	-1.8672056	-5.7186553
O	-0.5035643	-3.5567819	1.83378222	H	2.27114248	-3.6056851	-5.5813657
H	-1.1393274	-2.8187087	-0.5701343	H	2.41372546	-1.6252905	-3.2874697
H	1.41757552	-1.1912873	-0.9699809	H	1.09015102	-6.1864093	-2.1537835
C	0.43712018	-3.0547432	-4.6266505	H	1.25520294	-5.5511535	-3.7916375
O	-0.7325249	-3.3473702	-4.8947647	H	2.45959086	-5.1425006	-2.5566664
C	1.45816253	-2.8894139	-5.7224107	H	-0.002352	0.25058908	-3.3706309
O	2.30786088	-2.578031	-3.080537	H	2.31607091	-1.6270598	4.67801338
C	1.39156938	-5.3070545	-2.7335008	H	0.70444622	-2.0300021	5.30233766
H	-1.8602254	1.56873235	0.77088243	H	1.30300932	-2.7805239	3.78979359
O	0.69829303	-0.4393168	-3.3479152	H	-4.0166934	-0.7447514	-2.2450191
O	0.76475336	-0.773776	3.6535397	H	-4.2365079	0.67356254	-1.1628984
C	1.28901155	-1.8686504	4.39160299	H	-4.1402397	0.89578474	-2.9199683
O	-1.8050332	-0.1961178	-0.8917155				

conformer **4a**_6							
C	0.36366896	2.95582557	1.0854974	O	-0.3143895	0.39914539	-3.1222272
C	-0.5084762	0.31344331	1.75748972	C	-1.2583411	0.32603138	-2.3519916
C	-0.1751815	1.29887077	2.89529387	C	-2.6610558	0.72214064	-2.6961025
C	-0.0105352	2.70845558	2.36182377	H	-1.0123121	1.30019351	3.60730408
C	0.06826886	-0.5739729	-0.5447038	H	0.83014293	-0.5944195	-1.3323173
C	-0.1660803	-2.0292061	-0.0463943	H	0.12216372	-1.5110127	2.78635387
C	-1.1721764	-2.0686039	1.12070222	H	-1.5498183	-2.5624683	-1.6314525
C	-0.75689	-1.1195547	2.26560673	H	-2.8195872	-4.1286999	0.18284368
C	-0.6341729	-2.963416	-1.1846843	H	-2.0995866	-3.5326607	2.42993751
C	-0.8678458	-4.4150116	-0.7071145	H	-0.4144008	-3.8733118	2.05779576
C	-1.8239539	-4.4610672	0.50555255	H	2.76062862	2.7579638	0.29201043
C	-1.3559779	-3.5116425	1.6242664	H	3.82005961	0.64681716	0.66270112

C	0.52150171	0.37007704	0.60950084	H	3.69251927	1.71790914	-1.8543729
C	0.76215304	1.8403001	0.10731617	H	2.09001249	1.17429374	-2.2582022
C	2.28347773	1.91195744	-0.2166752	H	0.77258578	2.62481828	-3.6257166
N	2.84659455	0.73910701	0.39812087	H	0.3730986	4.83164009	-4.6451146
C	1.92471571	-0.060995	1.01768584	H	1.75852244	6.78301967	-3.9899169
O	2.23347884	-1.0240582	1.70092551	H	3.56192698	6.51095452	-2.3085357
C	2.6291513	1.95871121	-1.7159533	H	3.97613656	4.31032517	-1.2756849
C	2.38719004	3.30609042	-2.3540362	H	0.06295109	4.33366551	-0.5436961
C	1.38233542	3.47037764	-3.3155179	H	-0.2496723	5.04893941	1.01257002
C	1.15491833	4.71804487	-3.898738	H	1.42761611	4.743567	0.52079255
C	1.9340812	5.81315001	-3.5323551	H	0.03456739	4.77207127	3.08659034
C	2.94635909	5.65988858	-2.5878427	H	-1.3726649	3.82022499	3.59378129
C	3.1752329	4.41251927	-2.0047178	H	0.22291265	3.55452595	4.3248301
C	0.40912013	4.34671348	0.49479815	H	-1.7416115	-1.8593585	3.78392101
C	-0.2984736	3.77623125	3.38780854	H	-2.9454584	-5.6206646	-3.2941688
H	-1.4728634	0.66411049	1.35449935	H	-3.325533	-4.3784476	-2.0931526
O	-1.8258601	-1.071197	3.21865981	H	-2.1581199	-4.0322598	-3.4095985
H	-2.1564314	-1.7389973	0.75634322	H	0.4027074	-5.8949554	-0.5626845
H	0.798991	-2.4136733	0.30527494	H	-2.6808853	-5.8612041	1.9340574
C	-1.3997982	-5.2978898	-1.8586152	H	-2.3878019	-6.5666573	0.34605141
O	-0.9107235	-6.4175848	-2.0508002	H	-1.0307252	-6.2721179	1.44919852
C	-2.529126	-4.7902888	-2.7163138	H	1.11396008	-3.4987959	-1.843296
O	0.4182153	-4.94384	-0.320686	H	1.86160993	0.32576917	5.30970575
C	-1.9839711	-5.8718788	1.08872202	H	0.44130314	1.37491473	5.48822094
H	0.17213717	2.02034661	-0.7971592	H	0.24928405	-0.3272508	4.96202732
O	0.36988212	-2.9725121	-2.2046914	H	-2.9681576	1.56915205	-2.0776414
O	1.04854761	0.96745253	3.54624874	H	-2.7060024	1.02021037	-3.747466
C	0.87790898	0.56472593	4.89683996	H	-3.3328688	-0.1272387	-2.5481068
O	-1.205109	-0.0977789	-1.0612257				

Table S5. Cartesian coordinates for the low-energy optimized conformers of **5a** at B3LYP-D3(BJ)/6-311G* level.

conformer 5a_1							
C	−1.9354897	0.41203325	−2.5608914	O	−1.2073567	1.20279943	2.91772139
C	0.74452737	0.08426841	−1.6210165	C	−0.8257522	1.86973884	1.96548565
C	0.43649849	−0.2167344	−3.1041237	C	−0.9796963	3.35329224	1.84374492
C	−0.9360214	0.31795163	−3.4682312	H	1.19833324	0.2811324	−3.7170021
C	−0.0230592	−0.0703207	0.78924113	H	−0.7316124	−0.5715536	1.45724812
C	1.41617982	−0.4496159	1.23025593	H	2.31059526	−1.4156036	−1.3214586
C	2.47368785	0.08873187	0.24888094	H	1.61376678	1.08968737	2.77572045
C	2.1750187	−0.3336543	−1.2127909	H	4.25830053	1.17201567	2.12021843
C	1.7326229	0.00501494	2.67384268	H	4.63041348	0.06097634	0.00368729
C	3.15756227	−0.3989734	3.12720221	H	3.95890129	−1.4565595	0.59186979
C	4.22388024	0.0736083	2.11365529	H	−3.3419318	−1.5179216	−1.6401854
C	3.88066171	−0.3650995	0.68076399	H	−2.0206326	−3.3499108	−0.7553366
C	−0.3342728	−0.4898321	−0.6752984	H	−4.0122267	−2.1065885	0.70506955
C	−1.7824723	−0.0472599	−1.1050731	H	−2.9011044	−0.934126	1.34900184
C	−2.6891064	−1.2706846	−0.7955565	H	−3.799772	1.17528167	1.93018578
N	−1.7714725	−2.3710863	−0.676314	H	−5.5769263	2.87485414	1.76450178
C	−0.4492728	−2.0111162	−0.6920117	H	−7.4498258	2.57447463	0.16693539
O	0.45832006	−2.823281	−0.620973	H	−7.5448752	0.55121954	−1.2637065
C	−3.539995	−1.1395021	0.482793	H	−5.7750891	−1.159315	−1.1177124
C	−4.6353731	−0.1049229	0.39448843	H	−3.5624302	1.74551526	−2.0976657
C	−4.6031247	1.03372986	1.21080828	H	−3.2789012	1.59718863	−3.8185809
C	−5.6099065	1.99638164	1.12512493	H	−4.0491606	0.26560686	−2.9567027
C	−6.663641	1.82662561	0.22988092	H	−1.1222505	1.89330417	−4.9179337
C	−6.7162395	0.69007115	−0.573975	H	−0.2417034	0.49395357	−5.5177677
C	−5.7104056	−0.2738485	−0.4890354	H	−1.9886868	0.39171995	−5.3533148
C	−3.2765056	1.03544119	−2.8807561	H	3.73342884	0.05003942	6.58431551
C	−1.0842785	0.79932464	−4.8901028	H	2.64554452	−1.1536099	5.86311701
H	0.7131436	1.18313963	−1.5376574	H	4.41593931	−1.2418046	5.57669028
H	2.46969362	1.18803157	0.28852134	H	2.31714053	−2.0868027	3.56502169
H	1.46919763	−1.5458712	1.22656571	H	6.36717444	−0.0415911	1.75653485
C	3.40654375	0.29822767	4.48636337	H	5.93228903	−0.0897086	3.465767
O	3.4687679	1.52775822	4.58401655	H	5.68819573	−1.5207618	2.4483967
C	3.55912126	−0.5758994	5.70444578	H	−0.0084201	−0.088993	3.58693356
O	3.2027838	−1.8248873	3.23528242	H	0.89902153	−3.09667	−4.6700685
C	5.63031297	−0.427491	2.46950183	H	−0.2209956	−1.8625131	−5.2556209
H	−2.0935373	0.81360079	−0.5027384	H	1.53234992	−1.4992956	−5.1096923
O	0.80446487	−0.6421318	3.56552964	H	4.64184101	0.09825999	−3.3839536
O	0.4787523	−1.6260496	−3.3184467	H	4.56420441	−1.1116122	−2.086347
C	0.68301224	−2.0246353	−4.6634072	H	3.3920465	−1.1606596	−3.4387289
O	3.09991497	0.36901693	−2.0630164	H	−1.535921	3.73142665	2.70614094
C	3.96628277	−0.5086636	−2.7754878	H	0.00480766	3.8269673	1.82468699
O	−0.1845292	1.37114308	0.87517763	H	−1.5412711	3.59360684	0.93734453

conformer 5a_2							
C	-2.1494201	0.55180912	-2.4402543	O	-0.9771095	0.77787022	3.04803496
C	0.59084726	0.17123886	-1.7325322	C	-0.6708713	1.547096	2.14738299
C	0.17834511	-0.0018253	-3.2091933	C	-0.8279756	3.03430941	2.202304
C	-1.2310553	0.51170024	-3.4331737	H	0.85775004	0.59183049	-3.835739
C	0.01902999	-0.2491228	0.70474893	H	-0.6403213	-0.8201788	1.36659972
C	1.48354848	-0.6770018	0.98979737	H	2.16980944	-1.2982323	-1.6806214
C	2.47114635	-0.0135006	0.01204128	H	1.81443559	0.66443843	2.68786864
C	2.05774281	-0.2307704	-1.4648399	H	4.41901767	0.80777485	1.8357979
C	1.91345195	-0.4007243	2.44957352	H	4.59998018	-0.0643149	-0.4277342
C	3.3660759	-0.8582181	2.73998258	H	3.93158094	-1.6192497	0.04590917
C	4.36042367	-0.2817054	1.70429105	H	-3.4921903	-1.4319603	-1.5689697
C	3.89530468	-0.5434003	0.26253172	H	-2.1202695	-3.3337978	-1.0071109
C	-0.4075044	-0.5053688	-0.76926	H	-3.9773089	-2.2311764	0.75486101
C	-1.8801448	-0.0169214	-1.0378545	H	-2.8038963	-1.1299539	1.41351429
C	-2.7692789	-1.2634643	-0.762797	H	-3.6501653	0.88939307	2.2970889
N	-1.855209	-2.3733887	-0.8218821	H	-5.4158581	2.60296836	2.4425301
C	-0.5376974	-2.0138041	-0.9428175	H	-7.3942979	2.48982394	0.95153805
O	0.36601956	-2.8246426	-1.0581387	H	-7.6068104	0.63980208	-0.6859659
C	-3.5127753	-1.2495205	0.58687517	H	-5.8506788	-1.0821955	-0.851676
C	-4.5994806	-0.2068792	0.68694044	H	-3.7968069	1.72387924	-1.6974706
C	-4.5005631	0.8328374	1.62147752	H	-3.5437483	1.92250049	-3.4096132
C	-5.5004145	1.80228285	1.71225075	H	-4.2697825	0.41894877	-2.8095102
C	-6.6134851	1.73741035	0.87706968	H	-2.5499191	1.08014867	-5.0822518
C	-6.7321917	0.69818756	-0.0430417	H	-0.9740187	1.89438896	-5.0552098
C	-5.7334672	-0.2725094	-0.134559	H	-1.1178856	0.19805603	-5.557597
C	-3.5134478	1.1820075	-2.6058297	H	4.80553881	-1.9664511	4.97439108
C	-1.4895821	0.95010828	-4.8533823	H	4.21979006	-0.790577	6.1678599
H	0.52594131	1.25184815	-1.5358316	H	3.06473035	-1.9061323	5.41016673
H	2.47892016	1.06432589	0.22006493	H	2.5386731	-2.5810955	3.05159953
H	1.52937804	-1.7642524	0.84732327	H	6.46627055	-0.3773615	1.16632313
C	3.72922255	-0.3146456	4.14249658	H	6.16453659	-0.6262759	2.88689234
O	3.81104037	0.89725009	4.36651881	H	5.81257331	-1.9213592	1.72857185
C	3.969022	-1.3165427	5.24222198	H	0.24501978	-0.5986785	3.47494845
O	3.40039631	-2.286908	2.68749128	H	1.11953382	-2.7537661	-4.7790688
C	5.77925008	-0.8380548	1.88462812	H	1.11968061	-1.107967	-5.442295
H	-2.1315343	0.7930893	-0.3441394	H	2.19311354	-1.5335331	-4.0721638
O	1.04857757	-1.1459442	3.32692243	H	2.18168677	2.34478872	-2.3759747
O	0.16880114	-1.3655093	-3.6169467	H	3.62121472	2.0849831	-1.3367458
C	1.21157102	-1.6947666	-4.5233612	H	3.78187387	2.13553485	-3.0920299
O	2.96379657	0.40800992	-2.3830216	H	-1.313669	3.31251317	3.14187753
C	3.130425	1.81376987	-2.2742579	H	0.15390946	3.51176703	2.15861622
O	-0.1204232	1.17456086	0.9603565	H	-1.4577391	3.37039652	1.37458147

conformer 5a_3							
C	-1.9866989	0.8092511	-2.6756936	O	-0.8650752	2.26377897	2.6027084
C	0.69023186	0.28946199	-1.8032972	C	-0.4632859	2.74628032	1.55278556
C	0.26175764	-0.1805214	-3.2114299	C	-0.438901	4.20889946	1.23661724
C	-1.0604776	0.45993167	-3.5980077	H	1.04071433	0.12745882	-3.9200312
C	0.04582896	0.58299623	0.63312697	H	-0.6790612	0.26924646	1.39310667
C	1.45476559	0.0966669	1.06898926	H	2.084847	-1.3325504	-1.3406293
C	2.50973002	0.35394895	-0.0233726	H	1.91331134	1.80333175	2.36261026
C	2.0825437	-0.2379491	-1.3920999	H	4.50768499	1.47242584	1.59391131
C	1.9028584	0.70882347	2.41616701	H	4.63190537	0.03141068	-0.3457878
C	3.29645442	0.20493865	2.86680716	H	3.82686435	-1.2935108	0.48873163
C	4.34964992	0.3965349	1.75264128	H	-3.514017	-0.8079998	-1.4320405
C	3.87816085	-0.1992428	0.41629107	H	-2.4167045	-2.5769973	-0.2247098
C	-0.3902331	-0.0014528	-0.741484	H	-2.9584505	0.26108778	1.40247899
C	-1.7946862	0.5509594	-1.178492	H	-4.0957152	0.99777881	0.29323334
C	-2.8199583	-0.485482	-0.6471068	H	-6.1447875	-0.2728285	-0.4756296
N	-2.0346466	-1.6428427	-0.3215995	H	-7.8835522	-1.8703668	0.24299763
C	-0.6869391	-1.4790434	-0.5069721	H	-7.447455	-3.4312814	2.1199044
O	0.11922343	-2.3844435	-0.3730073	H	-5.2569587	-3.3910984	3.28267677
C	-3.6200208	0.04379266	0.55568134	H	-3.5043109	-1.802492	2.57888458
C	-4.69787	-0.9163443	1.00316468	H	-3.346547	2.45893885	-2.4537523
C	-5.9376281	-0.9492769	0.3502319	H	-3.3560302	1.78921254	-4.0790717
C	-6.9236258	-1.8514146	0.75246004	H	-4.1424047	0.90631768	-2.77733
C	-6.6791888	-2.7290682	1.80705287	H	-2.1243021	0.21988917	-5.4562266
C	-5.4487296	-2.70616	2.46066601	H	-1.334164	1.81066095	-5.2529826
C	-4.4613803	-1.8049085	2.06079909	H	-0.3821306	0.39219403	-5.6585843
C	-3.2741638	1.5252513	-3.0218239	H	4.58404975	-0.4258161	5.36487515
C	-1.2389096	0.73525566	-5.0700367	H	4.11144729	1.07047654	6.19420567
H	0.79240567	1.38420514	-1.8850949	H	2.85412774	-0.0823339	5.70173014
H	2.63319339	1.43961554	-0.1503008	H	2.29655893	-1.290099	3.58213423
H	1.38020192	-0.987165	1.22562884	H	6.44256663	-0.0243184	1.33166811
C	3.69992767	1.05367693	4.09654837	H	6.10310384	0.22562406	3.0449027
O	3.90924536	2.2675365	4.00754639	H	5.64199954	-1.2969652	2.26285075
C	3.81792732	0.3509699	5.42446009	H	0.21498866	0.95067325	3.40062556
O	3.18622472	-1.1860209	3.18289947	H	1.11725446	-1.8692436	-5.0283128
C	5.70877333	-0.2129839	2.12297058	H	0.28763828	-3.286112	-4.3570922
H	-1.9703373	1.52336356	-0.7019021	H	-0.6724086	-1.9969493	-5.0912494
O	0.96023846	0.30944214	3.42989335	H	3.07245112	-1.5275511	-3.5071293
O	0.12577516	-1.5995444	-3.2179979	H	4.45773888	-0.4341704	-3.6952729
C	0.22125535	-2.2036105	-4.4973769	H	4.31635122	-1.4189714	-2.224289
O	3.03249555	0.21402886	-2.3745075	H	-0.9149752	4.7632676	2.05031546
C	3.75294194	-0.8578685	-2.9750636	H	0.59503811	4.54789748	1.13640945
O	0.05181392	2.03115207	0.51595467	H	-0.9975354	4.39834459	0.31644362

conformer 5a_4							
C	-3.358402	-0.321416	-0.8488869	O	0.22518128	1.57062316	3.06051347

C	-0.7631629	0.43710814	-1.7857128	C	-0.314787	2.17280459	2.14299332
C	-1.7520108	-0.2084703	-2.7793602	C	-0.9973268	3.4983233	2.27323597
C	-3.1467539	-0.2774829	-2.1842634	H	-1.8000855	0.41436105	-3.6828408
C	0.20606171	0.43745101	0.56345936	H	0.27517611	-0.1539143	1.48311537
C	1.63752762	0.60760409	-0.0135654	H	1.06580074	-0.3597222	-2.6058921
C	1.61964305	1.31467119	-1.381661	H	2.1979616	2.33766763	1.20486244
C	0.65069718	0.62705232	-2.3760171	H	3.67921195	3.15056644	-0.9580033
C	2.58998532	1.34482018	0.95659239	H	3.0330011	1.88449503	-2.932744
C	4.02603393	1.48785921	0.39001812	H	3.40578459	0.35327325	-2.1549292
C	4.01785877	2.10741725	-1.0275362	H	-3.1008565	-2.3580943	0.64166234
C	3.04456268	1.37231767	-1.9631718	H	-0.9808103	-3.4696189	0.46844617
C	-0.7564301	-0.2859937	-0.4239373	H	-1.2878514	-1.081989	2.77770001
C	-2.2097345	-0.3970364	0.16421217	H	-3.0301459	-0.9167594	2.75769787
C	-2.2307791	-1.7523171	0.92125025	H	-4.5148747	-2.9396519	3.05324817
N	-1.0823847	-2.4615267	0.42645678	H	-4.7325174	-5.1000946	4.22639462
C	-0.3193693	-1.7453795	-0.4603583	H	-2.7133563	-6.295827	5.0279162
O	0.62948694	-2.2171582	-1.0635004	H	-0.4638476	-5.3213788	4.65110681
C	-2.2088283	-1.5767831	2.44945887	H	-0.2239896	-3.1628847	3.47896601
C	-2.3527607	-2.8886709	3.18576358	H	-4.7161046	0.24853702	0.72608128
C	-3.6192118	-3.4494932	3.4001877	H	-5.4650002	0.23678575	-0.8514596
C	-3.7472936	-4.6713991	4.06229505	H	-5.0994347	-1.3040463	-0.0418312
C	-2.6129688	-5.3440975	4.51280536	H	-4.0140603	-1.0658656	-3.9867946
C	-1.3491886	-4.7963854	4.30159543	H	-5.2189222	-0.5808566	-2.8145735
C	-1.21835	-3.5746954	3.64026798	H	-4.3273057	0.65990538	-3.7123426
C	-4.7362709	-0.289051	-0.2283124	H	5.57610382	1.05633627	2.78335788
C	-4.2407725	-0.3151311	-3.2222685	H	6.70272094	1.48663257	1.45313087
H	-1.1574603	1.44679007	-1.5966813	H	6.37749885	2.64063474	2.76183318
H	1.28580889	2.3482779	-1.2227073	H	4.26531721	-0.2898923	1.11956457
H	2.04586423	-0.4020531	-0.1497582	H	5.38089567	2.60842965	-2.6475209
C	4.7872634	2.43470885	1.34834378	H	6.12503798	2.68324532	-1.0493994
O	4.46515439	3.61991804	1.47923984	H	5.80685945	1.11218446	-1.8065835
C	5.93584871	1.85968066	2.13622581	H	1.92620634	0.83425636	2.73858961
O	4.61373182	0.18531432	0.33566008	H	-0.0314937	-1.1234004	-4.6115436
C	5.41362568	2.12432111	-1.6652956	H	-0.6940356	-2.7670824	-4.584626
H	-2.3893268	0.42896274	0.86326674	H	-1.7139561	-1.4284085	-5.1471656
O	2.68637846	0.57612273	2.17036296	H	-0.7725653	2.80183508	-3.2278619
O	-1.3985386	-1.54914	-3.1007268	H	0.95184478	3.29369468	-3.1647607
C	-0.9345574	-1.7113564	-4.4332281	H	0.16710311	2.97848627	-4.7121188
O	0.61242858	1.29646508	-3.6496984	H	-1.013944	3.79524819	3.32567578
C	0.21720872	2.65995702	-3.666799	H	-0.450739	4.25281601	1.70229442
O	-0.3555552	1.74642186	0.85050483	H	-2.0287545	3.42014045	1.92008395

conformer 5a_5							
C	-3.358402	-0.321416	-0.8488869	O	0.22518128	1.57062316	3.06051347
C	-0.7631629	0.43710814	-1.7857128	C	-0.314787	2.17280459	2.14299332

C	-1.7520108	-0.2084703	-2.7793602	C	-0.9973268	3.4983233	2.27323597
C	-3.1467539	-0.2774829	-2.1842634	H	-1.8000855	0.41436105	-3.6828408
C	0.20606171	0.43745101	0.56345936	H	0.27517611	-0.1539143	1.48311537
C	1.63752762	0.60760409	-0.0135654	H	1.06580074	-0.3597222	-2.6058921
C	1.61964305	1.31467119	-1.381661	H	2.1979616	2.33766763	1.20486244
C	0.65069718	0.62705232	-2.3760171	H	3.67921195	3.15056644	-0.9580033
C	2.58998532	1.34482018	0.95659239	H	3.0330011	1.88449503	-2.932744
C	4.02603393	1.48785921	0.39001812	H	3.40578459	0.35327325	-2.1549292
C	4.01785877	2.10741725	-1.0275362	H	-3.1008565	-2.3580943	0.64166234
C	3.04456268	1.37231767	-1.9631718	H	-0.9808103	-3.4696189	0.46844617
C	-0.7564301	-0.2859937	-0.4239373	H	-1.2878514	-1.081989	2.77770001
C	-2.2097345	-0.3970364	0.16421217	H	-3.0301459	-0.9167594	2.75769787
C	-2.2307791	-1.7523171	0.92125025	H	-4.5148747	-2.9396519	3.05324817
N	-1.0823847	-2.4615267	0.42645678	H	-4.7325174	-5.1000946	4.22639462
C	-0.3193693	-1.7453795	-0.4603583	H	-2.7133563	-6.295827	5.0279162
O	0.62948694	-2.2171582	-1.0635004	H	-0.4638476	-5.3213788	4.65110681
C	-2.2088283	-1.5767831	2.44945887	H	-0.2239896	-3.1628847	3.47896601
C	-2.3527607	-2.8886709	3.18576358	H	-4.7161046	0.24853702	0.72608128
C	-3.6192118	-3.4494932	3.4001877	H	-5.4650002	0.23678575	-0.8514596
C	-3.7472936	-4.6713991	4.06229505	H	-5.0994347	-1.3040463	-0.0418312
C	-2.6129688	-5.3440975	4.51280536	H	-4.0140603	-1.0658656	-3.9867946
C	-1.3491886	-4.7963854	4.30159543	H	-5.2189222	-0.5808566	-2.8145735
C	-1.21835	-3.5746954	3.64026798	H	-4.3273057	0.65990538	-3.7123426
C	-4.7362709	-0.289051	-0.2283124	H	5.57610382	1.05633627	2.78335788
C	-4.2407725	-0.3151311	-3.2222685	H	6.70272094	1.48663257	1.45313087
H	-1.1574603	1.44679007	-1.5966813	H	6.37749885	2.64063474	2.76183318
H	1.28580889	2.3482779	-1.2227073	H	4.26531721	-0.2898923	1.11956457
H	2.04586423	-0.4020531	-0.1497582	H	5.38089567	2.60842965	-2.6475209
C	4.7872634	2.43470885	1.34834378	H	6.12503798	2.68324532	-1.0493994
O	4.46515439	3.61991804	1.47923984	H	5.80685945	1.11218446	-1.8065835
C	5.93584871	1.85968066	2.13622581	H	1.92620634	0.83425636	2.73858961
O	4.61373182	0.18531432	0.33566008	H	-0.0314937	-1.1234004	-4.6115436
C	5.41362568	2.12432111	-1.6652956	H	-0.6940356	-2.7670824	-4.584626
H	-2.3893268	0.42896274	0.86326674	H	-1.7139561	-1.4284085	-5.1471656
O	2.68637846	0.57612273	2.17036296	H	-0.7725653	2.80183508	-3.2278619
O	-1.3985386	-1.54914	-3.1007268	H	0.95184478	3.29369468	-3.1647607
C	-0.9345574	-1.7113564	-4.4332281	H	0.16710311	2.97848627	-4.7121188
O	0.61242858	1.29646508	-3.6496984	H	-1.013944	3.79524819	3.32567578
C	0.21720872	2.65995702	-3.666799	H	-0.450739	4.25281601	1.70229442
O	-0.3555552	1.74642186	0.85050483	H	-2.0287545	3.42014045	1.92008395

conformer 5a_6							
C	-2.2023126	1.05449473	-2.4694351	O	-0.8610516	1.85388038	2.9006471
C	0.53131193	0.56001824	-1.8090703	C	-0.5423071	2.4934824	1.90763974
C	0.03484087	0.21195568	-3.2308554	C	-0.6058755	3.98430306	1.79255338

C	-1.3228643	0.84824131	-3.4772915	H	0.75152948	0.59846862	-3.9669571
C	0.01645349	0.50386242	0.67908935	H	-0.6507394	0.05365019	1.42336774
C	1.46688863	0.04001062	0.97320342	H	2.09903244	-0.9599286	-1.701006
C	2.44521418	0.51985729	-0.1143926	H	1.90160208	1.56451212	2.48569028
C	1.98792975	0.11934885	-1.5465477	H	4.45784439	1.51230508	1.57985161
C	1.94870571	0.47504715	2.37698856	H	4.56714708	0.40413161	-0.5568118
C	3.38576106	-0.0075238	2.68957094	H	3.88788222	-1.0896708	0.08427943
C	4.36861509	0.41694827	1.57668476	H	-3.5676831	-0.798589	-1.3896646
C	3.86600618	0.00336207	0.18382258	H	-2.3302373	-2.6420193	-0.473197
C	-0.4638824	0.0818116	-0.7360827	H	-2.9440166	-0.0605963	1.53470458
C	-1.9133255	0.61235721	-1.0289784	H	-4.1674852	0.73943927	0.5704739
C	-2.8573682	-0.5403075	-0.595028	H	-6.1734426	-0.5512833	-0.2844021
N	-1.9986065	-1.6844034	-0.4442556	H	-7.7925523	-2.3219676	0.29432329
C	-0.6738911	-1.4256354	-0.6849685	H	-7.1972304	-4.0656557	1.95408544
O	0.18723818	-2.2890015	-0.7295573	H	-4.9670092	-4.0348195	3.03913627
C	-3.6308505	-0.2104003	0.69317466	H	-3.3329438	-2.2738369	2.47325668
C	-4.635629	-1.2788611	1.05724125	H	-4.3369889	1.00974312	-2.7560496
C	-5.8970275	-1.3071601	0.44693606	H	-3.7595275	2.37538751	-1.7823511
C	-6.8155361	-2.3069515	0.77031361	H	-3.553551	2.41223329	-3.5152638
C	-6.4815273	-3.2873324	1.70276868	H	-1.3485216	0.31386431	-5.5583
C	-5.228762	-3.2697149	2.31273766	H	-2.6234249	1.43360103	-5.1353237
C	-4.3087342	-2.2710204	1.99145731	H	-0.9533724	2.01471596	-5.2390701
C	-3.5339896	1.7451788	-2.6496944	H	3.07896241	-0.7419642	5.45984638
C	-1.5830622	1.17603523	-4.9252836	H	4.80783777	-0.9381872	5.0166092
H	0.55004075	1.66228542	-1.772328	H	4.29922865	0.3888608	6.07983134
H	2.49795107	1.61826412	-0.0780473	H	2.50624097	-1.6529011	3.20522811
H	1.4592325	-1.0572265	0.96981157	H	6.45854397	0.19833766	1.01109026
C	3.80310313	0.66620199	4.01907637	H	6.19136501	0.16297583	2.75471401
O	3.95377061	1.88867188	4.10831187	H	5.77825865	-1.2491318	1.76594044
C	4.00876993	-0.2211282	5.21965487	H	0.30646596	0.48098855	3.46088451
O	3.36654302	-1.4343992	2.78826647	H	1.64547905	-1.9008171	-4.0299717
C	5.77685758	-0.1544468	1.79279824	H	0.22870074	-2.8076368	-4.564543
H	-2.1056479	1.50183918	-0.4165441	H	0.58976742	-1.247163	-5.3279766
O	1.07822013	-0.1202892	3.35810228	H	3.24883548	-0.4421446	-4.0171863
O	-0.1829319	-1.1868215	-3.3950883	H	4.51656498	0.712588	-3.5983481
C	0.61937811	-1.8020242	-4.3858248	H	4.2353288	-0.6903899	-2.545879
O	2.81867119	0.85315657	-2.4681803	H	-1.0630891	4.39805032	2.69578503
C	3.74961672	0.05251899	-3.1839577	H	0.40348351	4.39016935	1.69032069
O	-0.0518424	1.95252801	0.759114	H	-1.2223802	4.26366626	0.93432894

conformer 5a_7							
C	-2.7868059	-2.1488581	0.70153646	O	1.00290873	1.35235134	2.86264942
C	-1.5479424	-0.4107562	-1.1630086	C	-0.1582885	1.40482	2.48236415
C	-2.525178	-1.4785978	-1.6946601	C	-1.2738797	2.05342201	3.24018175
C	-3.3441243	-1.9997665	-0.5236098	H	-3.204224	-1.0336083	-2.4327044

C	0.29053366	0.11375802	0.4984016	H	1.04721829	-0.3429498	1.14779485
C	0.99773472	0.99878877	-0.5685609	H	-0.270925	-0.1677771	-2.9239005
C	0.00082241	1.55970374	-1.6028628	H	1.21102139	2.79583467	0.66662686
C	-0.8747875	0.44891098	-2.2513508	H	0.90008292	4.22252116	-1.6159645
C	1.83755186	2.1381725	0.05375073	H	0.02383843	2.82092346	-3.3688762
C	2.57424528	2.98887554	-1.0089246	H	1.38096289	1.70196448	-3.2719138
C	1.59353656	3.50892681	-2.0823968	H	-1.1211621	-4.0142614	0.49170918
C	0.74697401	2.37303417	-2.6778713	H	1.10512744	-3.8598071	-0.290725
C	-0.5548891	-1.0191803	-0.1543085	H	-1.0683728	-4.0618271	2.88690228
C	-1.2916109	-1.8864604	0.93504327	H	0.4357674	-4.6884757	2.26068361
C	-0.5185689	-3.2306879	0.97300617	H	2.48547835	-2.9978193	2.14733915
N	0.6091903	-3.0666465	0.09892143	H	3.73795895	-1.4573771	3.60646424
C	0.43085456	-2.0100262	-0.7594357	H	2.63234239	-0.4075374	5.55991576
O	1.05232859	-1.8887791	-1.8029661	H	0.25622425	-0.9073228	6.05663183
C	-0.1484342	-3.7644155	2.36839265	H	-1.021637	-2.4383229	4.60486615
C	0.63251942	-2.8257828	3.2577156	H	-3.227081	-2.0357398	2.80373641
C	1.98036485	-2.5453934	2.99775875	H	-4.6228478	-2.4491585	1.83834235
C	2.69594217	-1.6777337	3.82351797	H	-3.3602954	-3.6657323	2.11180911
C	2.0750372	-1.0889384	4.92265619	H	-5.3415105	-1.4380363	-1.0624202
C	0.73805837	-1.3665707	5.19772481	H	-4.7829279	-2.9562093	-1.794325
C	0.01998226	-2.2312244	4.37081452	H	-5.2754593	-2.9336987	-0.1136601
C	-3.5443819	-2.6041365	1.92274579	H	4.99063255	5.11760854	0.44671926
C	-4.7635985	-2.3499714	-0.8818519	H	5.03467304	3.34325907	0.43944924
H	-2.1866938	0.30003458	-0.6110222	H	5.16686514	4.26115372	-1.0978051
H	-0.6753108	2.26105724	-1.0915667	H	3.9032448	1.58646997	-0.9159704
H	1.70652488	0.34842264	-1.0962641	H	1.58702633	4.64318129	-3.9394732
C	3.19816029	4.19351347	-0.2625927	H	2.8887063	5.09831192	-2.8385835
O	2.50210243	5.09674773	0.21149237	H	2.99648221	3.58970487	-3.7629254
C	4.69749884	4.22398928	-0.1117868	H	2.42602963	1.39588071	1.77106983
O	3.56671391	2.16699092	-1.6313484	H	-2.8372066	-2.5815657	-4.0574634
C	2.31224718	4.24927628	-3.2189876	H	-1.1512764	-1.9587756	-4.0327083
H	-1.2158007	-1.3719882	1.89804664	H	-1.4623293	-3.6922414	-3.9124166
O	2.84751822	1.54589587	0.89259787	H	-2.1182937	-0.0298968	-4.6976972
O	-1.8747502	-2.6360355	-2.2159701	H	-2.5209425	1.68434271	-4.8521641
C	-1.8377072	-2.7047153	-3.6299805	H	-0.816398	1.19542095	-4.7539712
O	-1.9264127	1.10852994	-2.9818775	H	-0.9134366	2.35429856	4.22795302
C	-1.8250028	0.97649798	-4.3938354	H	-1.6190489	2.94014476	2.70321254
O	-0.617027	0.92039702	1.29665843	H	-2.092464	1.34166711	3.37550577

conformer 5a_8							
C	-1.2399745	0.99581503	-2.6653312	O	-0.7385122	2.57418112	1.96330148
C	0.68664102	-0.6780943	-1.3807489	C	0.32867206	2.42851716	1.38896249
C	0.28390549	-0.9926397	-2.8374673	C	1.34349172	3.5145217	1.20556835
C	-0.4745515	0.18090162	-3.4273215	H	1.20120659	-1.1634669	-3.4146729
C	-0.0497289	0.08908167	0.92046264	H	-0.9122667	0.26733406	1.57319027

C	0.84597508	-1.0031688	1.57255567	H	1.03976786	-2.7354019	-0.7133515
C	2.03604393	-1.3718645	0.66462941	H	1.90125796	0.36801339	2.88353198
C	1.57409858	-1.7800241	-0.756932	H	4.07868534	-1.2148655	2.57947017
C	1.35211521	-0.5753439	2.96860292	H	3.75641059	-2.6942154	0.68224392
C	2.241673	-1.6479298	3.63695873	H	2.30494305	-3.401528	1.38340843
C	3.40273123	-2.0703249	2.7105512	H	-3.5137571	0.31177496	-1.7520481
C	2.88947599	-2.4746541	1.31666003	H	-3.5712048	-1.6597385	-0.3734776
C	-0.5364388	-0.3274263	-0.4994437	H	-4.5003952	0.70603503	0.58618288
C	-1.4408644	0.778624	-1.1595514	H	-2.9751478	1.37805744	1.08468888
C	-2.9040881	0.35956955	-0.8419925	H	-2.6650778	3.73988204	0.80944781
N	-2.8040631	-0.9984668	-0.3823882	H	-3.4208751	5.90348412	-0.0947275
C	-1.5235574	-1.4758669	-0.3136749	H	-5.2795459	5.99420152	-1.7359313
O	-1.2687448	-2.6381548	-0.0424623	H	-6.3902967	3.8991316	-2.4641437
C	-3.6147522	1.23471951	0.20659682	H	-5.6457359	1.72317812	-1.5721767
C	-4.0818324	2.56612132	-0.3318758	H	-1.7524861	3.08528683	-2.561964
C	-3.4728103	3.75743066	0.08126345	H	-1.5671931	2.51536695	-4.2057123
C	-3.9001765	4.98581749	-0.4255114	H	-3.0060984	2.05681706	-3.2950277
C	-4.9450422	5.03685088	-1.3454855	H	0.39312096	1.27277422	-5.0611888
C	-5.5682484	3.86001272	-1.7542621	H	0.21293675	-0.4471638	-5.3878616
C	-5.1421395	2.63116381	-1.2476802	H	-1.2136439	0.57696935	-5.4195042
C	-1.9289254	2.22500187	-3.2157768	H	4.07360117	0.40251606	4.27236693
C	-0.2628698	0.41049865	-4.9028931	H	2.58283135	0.97909905	5.08532031
H	1.32095462	0.22107273	-1.4474383	H	3.93157689	0.31767942	6.03371195
H	2.69128964	-0.494083	0.56176516	H	1.73794419	-3.1874888	4.73236344
H	0.22344341	-1.8941155	1.71810915	H	5.05782068	-3.4791455	2.60389579
C	2.78068578	-1.1506894	4.99750694	H	4.68679131	-2.9518867	4.24392728
O	2.73880113	-1.8942025	5.98515803	H	3.63515782	-4.1261124	3.43111806
C	3.37684845	0.22919959	5.09496792	H	-0.1483957	-1.2184959	4.02075844
O	1.39874697	-2.78886	3.90236147	H	-1.0858189	-3.7904613	-3.9588307
C	4.23758664	-3.2237684	3.28390683	H	-1.222035	-2.2507726	-4.8142006
H	-1.2215587	1.74752637	-0.6997279	H	0.39040061	-2.9910655	-4.5312926
O	0.21692013	-0.3339707	3.80705089	H	3.02666136	-3.9526447	-1.2563121
O	-0.533176	-2.1610925	-2.860689	H	2.08879803	-3.5121603	-2.7170344
C	-0.6060183	-2.8200014	-4.113609	H	3.84428984	-3.2544995	-2.6697435
O	2.75591673	-1.9125565	-1.5693908	H	2.2996135	3.20476457	1.63522891
C	2.92780863	-3.2333617	-2.0742796	H	1.45389987	3.74134161	0.14228847
O	0.7695112	1.28493111	0.80043045	H	1.0069948	4.41650747	1.72462113

Table S6. Cartesian coordinates for the low-energy optimized conformers of **6a** at B3LYP-D3(BJ)/6-311G* level.

conformer 6a_1							
C	-1.1992672	2.9513075	-0.7081099	C	5.32923108	-2.913279	1.60780801
C	1.09714471	1.53186752	0.27789785	H	-1.8973542	1.10595818	0.0780239
C	1.27141671	2.97483704	-0.2476128	O	0.43023756	-3.4914135	0.75698565
C	-0.0495053	3.64958745	-0.5589122	H	1.91557132	3.00334096	-1.1338462
C	0.07706527	-0.7454484	-0.1201831	H	-0.4594978	-1.3684157	-0.8438425

C	1.4756033	-1.4177069	0.07145415	H	3.27101707	1.49607524	0.76832485
C	2.60668301	-0.4713876	0.39899303	H	1.138967	-2.113241	2.09799252
C	2.42829928	0.85279785	0.51775487	H	3.78098436	-1.7929885	2.55946209
C	1.4402932	-2.5405264	1.13429674	H	4.25705084	-1.5115236	-0.4488722
C	2.80057492	-3.2576063	1.29825631	H	4.71991639	-0.3778163	0.81138517
C	3.94557114	-2.2506891	1.57455868	H	-2.2151512	1.74251851	-2.7042536
C	3.96043237	-1.1193561	0.53273542	H	-0.4963985	0.62759708	-3.8555418
C	0.15600544	0.71829561	-0.6239136	H	-2.9466436	-0.6439712	-3.0631187
C	-1.2434469	1.41149873	-0.7453192	H	-2.4523525	-1.05405	-1.4485577
C	-1.7800527	0.92210413	-2.1228472	H	-3.8850398	-0.5665939	0.4425082
N	-0.5896384	0.54189065	-2.8497754	H	-6.0757478	0.21000876	1.26748166
C	0.55614584	0.65753135	-2.0896145	H	-7.6080819	1.4553224	-0.2333086
O	1.6785389	0.65165932	-2.562863	H	-6.9421398	1.91908795	-2.5766794
C	-2.8091559	-0.2194003	-2.0591037	H	-4.7564191	1.14803938	-3.4239808
C	-4.1575444	0.23418032	-1.5528586	H	-3.3116379	3.03742366	-0.2890404
C	-4.5461262	-0.0251997	-0.2316568	H	-2.5641344	4.61336363	-0.3546068
C	-5.7834814	0.4143687	0.24064089	H	-2.8549193	3.72549735	-1.8631197
C	-6.6440628	1.11484549	-0.6021004	H	0.93785558	5.42411925	-1.268107
C	-6.2691169	1.37563107	-1.9184834	H	-0.8051606	5.57141685	-1.2651456
C	-5.0319739	0.93764063	-2.3929028	H	0.07759736	5.63288744	0.27383219
C	-2.5515821	3.62204776	-0.817548	H	1.31700268	3.77429737	1.53243909
C	0.03665216	5.15082227	-0.707847	H	-0.9057884	-1.7329443	1.24069484
O	1.92309876	3.74597681	0.77238151	H	2.5400074	-2.9831217	4.09764996
H	0.64008406	1.60593046	1.27771573	H	1.05000224	-3.8882857	3.67945363
O	-0.7079264	-0.7871424	1.08043074	H	2.40266635	-4.7007732	4.49565387
H	1.71947414	-1.8621406	-0.9051999	H	3.55839346	-4.769091	0.31474701
C	2.72925011	-4.3150584	2.42240955	H	5.5761909	-3.3834746	0.65033655
O	3.17043132	-5.4533777	2.2233856	H	6.10529236	-2.1706367	1.82359998
C	2.13831728	-3.9390501	3.75621958	H	5.38622595	-3.6776495	2.38875553
O	3.04875215	-3.96788	0.06589603	H	0.84530324	-4.0405354	0.0567109
conformer 6a_2							
C	-1.2012626	3.28350003	0.20750737	C	4.62334229	-3.3575466	2.29060952
C	0.87221228	1.5602563	1.20472067	H	-2.1082536	1.40289136	0.60589611
C	1.19445126	3.04654955	0.93108282	O	-0.1659869	-3.4100684	0.83681896
C	-0.0388434	3.8602923	0.59180975	H	1.93626425	3.15470065	0.13156588
C	-0.2378896	-0.5448255	0.36170324	H	-0.7308519	-1.013501	-0.4965012
C	1.08197533	-1.3494262	0.59515355	H	2.96711566	1.27972816	1.91303379
C	2.22523021	-0.5547189	1.18220956	H	0.46299198	-2.3025839	2.44247084
C	2.11999379	0.74917357	1.47992898	H	3.04765384	-2.2647949	3.22919379
C	0.8494722	-2.608001	1.46302373	H	3.89390306	-1.5922698	0.36772351
C	2.13253368	-3.4483226	1.66113911	H	4.27644386	-0.6933282	1.82836474
C	3.29850001	-2.5875343	2.20961868	H	-2.0363956	2.47560502	-2.0451313
C	3.50917268	-1.3228726	1.36006119	H	-0.2910819	1.36051884	-3.1755559
C	-0.0041322	0.96336576	0.0925208	H	-2.5564289	-0.4852903	-1.4024379
C	-1.3324183	1.77573126	-0.0735861	H	-3.7116468	0.79328046	-1.1151429
C	-1.7278522	1.54276481	-1.559646	H	-4.7686386	2.02140359	-3.0037103

N	-0.4890452	1.1691472	-2.1995436	H	-5.4963128	1.8822235	-5.3578795
C	0.56397214	1.07569734	-1.3144716	H	-4.4767717	0.21536272	-6.8847082
O	1.73193521	1.03377003	-1.65749	H	-2.7179652	-1.3200944	-6.0479802
C	-2.8543456	0.51158251	-1.7404254	H	-1.9746645	-1.1960962	-3.6972132
C	-3.3217738	0.41762698	-3.1754976	H	-3.3499887	3.45112463	0.29007761
C	-4.3118917	1.28363247	-3.6593281	H	-2.5249633	4.93603305	0.70794955
C	-4.7261069	1.20922371	-4.9900195	H	-2.5925865	4.41890001	-0.9917097
C	-4.1534658	0.27270831	-5.8486296	H	1.1190074	5.64241876	0.26140974
C	-3.165784	-0.5904058	-5.3782095	H	-0.6049303	5.91988379	0.13981671
C	-2.7502811	-0.5184691	-4.048083	H	0.1274398	5.67798353	1.73703112
C	-2.4819201	4.07059663	0.03938828	H	1.0889572	3.55909379	2.81196767
C	0.15268295	5.35600121	0.69131361	H	-1.4412348	-1.6224823	1.45074284
O	1.77070949	3.60611806	2.12014849	H	1.55921754	-3.5609691	4.42473383
H	0.30538881	1.52346388	2.14890961	H	0.0753025	-4.2743343	3.71518176
O	-1.153787	-0.6861582	1.45735531	H	1.26786233	-5.3015605	4.53898746
H	1.40951977	-1.6659058	-0.4065362	H	2.90480163	-4.8575688	0.54583632
C	1.8615127	-4.6479664	2.59626694	H	4.95169846	-3.7032996	1.3049823
O	2.25034158	-5.777285	2.2745794	H	5.41408756	-2.7188489	2.69944217
C	1.14147618	-4.4224302	3.90022201	H	4.53839839	-4.2285117	2.94756558
O	2.47928026	-3.9908829	0.36872257	H	0.29465308	-3.8840003	0.11064877
conformer 6a_3							
C	-1.3258202	2.79404439	-1.0537288	C	5.51826772	-2.7602971	1.11814219
C	1.13197974	1.51434639	-0.2927918	H	-1.8421968	1.19699153	0.24563055
C	1.18498212	2.78272292	-1.1769405	O	0.54775706	-3.2703894	1.39608113
C	-0.169891	3.44821123	-1.3156973	H	1.58063292	2.56855604	-2.1772486
C	0.03567177	-0.7454691	0.05361509	H	-0.6482655	-1.4787805	-0.3871676
C	1.43708516	-1.4355341	0.0892337	H	3.36623752	1.45059264	-0.228163
C	2.61756958	-0.501009	-0.0360023	H	1.52290373	-1.6790569	2.24346088
C	2.47837621	0.82581987	-0.1797415	H	4.19729625	-1.3918916	2.08833285
C	1.61721276	-2.310671	1.3523204	H	4.05424808	-1.7809918	-0.9426326
C	2.9782661	-3.0426142	1.39128692	H	4.77155223	-0.4301298	-0.0784392
C	4.15859678	-2.0553702	1.21375105	H	-2.7440646	1.24139374	-2.4811227
C	3.96628955	-1.1742902	-0.0318145	H	-1.3189147	-0.1821762	-3.688259
C	0.01199643	0.56993723	-0.7646358	H	-3.5404435	-1.1325339	-2.1426431
C	-1.3793025	1.28773876	-0.7424917	H	-2.7252622	-1.1985239	-0.609417
C	-2.1996888	0.54262616	-1.8359304	H	-3.7266853	-0.2154768	1.3686006
N	-1.1936045	-0.0473331	-2.6915682	H	-5.6974628	0.83327876	2.41615345
C	0.08773634	0.17499431	-2.2313557	H	-7.5108622	1.77329908	1.00932089
O	1.08483375	0.02029062	-2.914834	H	-7.3490606	1.65597865	-1.4625695
C	-3.1974039	-0.5032869	-1.3098205	H	-5.3872869	0.60797506	-2.5317903
C	-4.4110324	0.11774949	-0.660656	H	-3.2821185	3.07431923	-0.1936816
C	-4.5151	0.18672184	0.73510571	H	-2.5721685	4.56057378	-0.7698401
C	-5.6270589	0.78152794	1.33266363	H	-3.2048771	3.3873768	-1.9412651
C	-6.6453005	1.31073766	0.54237525	H	0.63256198	5.00262383	-2.5668547
C	-6.5534757	1.2451097	-0.8463987	H	-1.0625371	5.23025626	-2.2048486
C	-5.442034	0.65157114	-1.4463007	H	0.14743178	5.54666091	-0.944742

C	-2.6661027	3.49423871	-0.995574	H	2.95510153	3.54010039	-0.8861061
C	-0.1171753	4.88590834	-1.7761584	H	-0.6477019	-1.3675266	1.76918465
O	2.06147736	3.73313722	-0.5584551	H	1.74851444	-3.082159	4.15101223
H	0.9036352	1.84646658	0.73252785	H	3.23459833	-3.7737911	4.83592755
O	-0.4715824	-0.4907848	1.37075666	H	3.29771279	-2.183837	4.06398103
H	1.46436513	-2.0856394	-0.7981443	H	3.50405834	-4.7639033	0.62546126
C	3.13354531	-3.8399773	2.70543869	H	5.56490983	-3.435231	0.25725823
O	3.52055316	-5.0142651	2.66790891	H	6.3243688	-2.0263067	1.00945795
C	2.82927662	-3.1688984	4.01942455	H	5.72961678	-3.3421549	2.02045911
O	2.96803435	-4.0030192	0.31303881	H	0.80522741	-3.970558	0.75791911
conformer 6a_4							
C	-0.8670375	3.00686885	-0.897031	C	4.92689829	-3.3604646	1.96655057
C	1.24040395	1.41627561	0.22624653	H	-1.7759444	1.30092167	-0.0167537
C	1.58711026	2.78558487	-0.4017723	O	0.02278793	-3.4771567	0.97521631
C	0.35073264	3.58527801	-0.7670278	H	2.2275833	2.6660804	-1.2840496
C	-0.0130313	-0.7655989	-0.0682434	H	-0.5890587	-1.3695071	-0.7778762
C	1.29999673	-1.5691666	0.2052468	H	3.37862401	1.17145887	0.8008878
C	2.51873914	-0.7308751	0.50987773	H	0.83336852	-2.0973963	2.25655948
C	2.48191134	0.60934579	0.54579373	H	3.48025014	-2.0230581	2.78915311
C	1.11587776	-2.6134189	1.33137979	H	4.0694103	-1.9947538	-0.2124559
C	2.38637332	-3.4566654	1.58711856	H	4.61731946	-0.8354876	0.9887157
C	3.6237529	-2.5599096	1.84162876	H	-1.9813737	1.79443075	-2.8362533
C	3.78980592	-1.5088766	0.73151799	H	-0.3717466	0.44006759	-3.8833983
C	0.23279166	0.65110168	-0.6465739	H	-2.9468414	-0.5296827	-3.0677636
C	-1.0795564	1.48178256	-0.8420673	H	-2.5371732	-0.8752431	-1.4147828
C	-1.6441856	0.96911946	-2.1991209	H	-3.9386846	-0.0858367	0.40169827
N	-0.4888313	0.42353349	-2.8768536	H	-6.0533356	0.96986298	1.105477
C	0.64945912	0.46447085	-2.0975077	H	-7.4245719	2.23561108	-0.5283226
O	1.77416021	0.32301946	-2.5445598	H	-6.6729754	2.43913744	-2.8829167
C	-2.7888254	-0.0527158	-2.090657	H	-4.5616251	1.38778252	-3.6093655
C	-4.0933029	0.57245848	-1.6573983	H	-2.9647495	3.33218614	-0.5195874
C	-4.5298636	0.46130027	-0.3304506	H	-2.0560584	4.81641896	-0.6419733
C	-5.7243582	1.05916253	0.07329452	H	-2.4174514	3.89304772	-2.114523
C	-6.4940803	1.77130339	-0.8442279	H	1.50610112	5.21451602	-1.5602812
C	-6.0708698	1.88587391	-2.1668274	H	-0.2099428	5.5446668	-1.5515499
C	-4.8761786	1.28967886	-2.5727443	H	0.6902325	5.57738679	-0.0222754
C	-2.1403483	3.80885838	-1.0599073	H	2.89576313	4.14624924	0.08142877
C	0.58824212	5.06089781	-0.9817641	H	-1.1323235	-1.5731997	1.30693729
O	2.3256484	3.53309806	0.57385964	H	2.0758357	-2.9759462	4.35332743
H	0.77753251	1.61643043	1.20580143	H	0.51053381	-3.7459047	3.93931918
O	-0.8367295	-0.6626196	1.1014748	H	1.74537205	-4.6405468	4.85083761
H	1.51806551	-2.0972815	-0.7351988	H	3.00098087	-5.1023676	0.72738774
C	2.17002323	-4.4259089	2.77063747	H	5.14982209	-3.9169006	1.05030149
O	2.49272613	-5.6146874	2.65685483	H	5.77146538	-2.6907093	2.16273927
C	1.58465221	-3.9048304	4.05726857	H	4.87891652	-4.072723	2.79587599
O	2.59374793	-4.2674219	0.41003848	H	0.39803661	-4.1084066	0.32360898

conformer 6a_5							
C	-1.5634747	3.09272101	-0.5765344	C	5.45932783	-2.1131539	1.86853719
C	0.92740542	1.93965791	0.27592969	H	-2.0576692	1.46700992	0.6984438
C	0.947821	3.20892351	-0.6077335	O	0.51305728	-2.8641696	1.96444328
C	-0.4335734	3.80610194	-0.7927201	H	1.38676863	3.01422894	-1.5938917
C	-0.0704071	-0.3664576	0.59780791	H	-0.7072494	-1.133269	0.14419527
C	1.35936479	-0.9900194	0.68547621	H	3.15875136	1.98453837	0.42007269
C	2.49785739	-0.0001241	0.59803702	H	1.37661897	-1.2254949	2.84232811
C	2.300722	1.31800903	0.44095751	H	4.038645	-0.807587	2.78286888
C	1.53484423	-1.8529832	1.95742438	H	4.02619805	-1.2126419	-0.2494356
C	2.92758187	-2.5176263	2.0493004	H	4.64600316	0.17384897	0.63362025
C	4.06450972	-1.4747815	1.91064134	H	-2.8390312	1.48386232	-2.0547394
C	3.87608933	-0.6079358	0.65455876	H	-1.325005	0.07856998	-3.1906999
C	-0.1262667	0.93978044	-0.2329785	H	-2.7662446	-1.0369511	-0.2961826
C	-1.5507934	1.58690108	-0.2666279	H	-3.9680123	0.22250544	-0.1499427
C	-2.2900881	0.80668007	-1.3901678	H	-5.6300601	0.63220094	-1.9605933
N	-1.2265045	0.25600358	-2.1972515	H	-6.9238411	-0.3990686	-3.7919951
C	0.02503022	0.53763905	-1.6928396	H	-6.1332159	-2.4958154	-4.854114
O	1.05541974	0.41892414	-2.3325476	H	-4.0343406	-3.5645994	-4.0770294
C	-3.2745778	-0.2484199	-0.8589433	H	-2.7246433	-2.5479948	-2.2477694
C	-4.0845422	-0.8863087	-1.965278	H	-3.6009561	3.21188126	0.12092235
C	-5.2696689	-0.2880486	-2.4146406	H	-2.9163277	4.77114501	-0.2772088
C	-6.0041788	-0.8671076	-3.4504909	H	-3.3865519	3.66008649	-1.583363
C	-5.5602734	-2.0453874	-4.0478292	H	0.31718839	5.39744679	-2.0289722
C	-4.3813497	-2.6461137	-3.6106723	H	-1.3923757	5.55000682	-1.6902945
C	-3.6453959	-2.0694511	-2.5748466	H	-0.2165909	5.91585937	-0.4135346
C	-2.9386527	3.72187605	-0.5871951	H	2.66757631	4.05106768	-0.2559424
C	-0.438555	5.2458784	-1.2500768	H	-0.7896781	-0.9993431	2.29534479
O	1.7543288	4.2016024	0.03844039	H	1.60054423	-2.6106535	4.76254547
H	0.64583986	2.26120672	1.29139514	H	3.09281764	-3.2263535	5.50415065
O	-0.6295131	-0.1202793	1.8953697	H	3.10585769	-1.6372913	4.72791228
H	1.44869969	-1.6402598	-0.1977006	H	3.56486051	-4.2129005	1.31051503
C	3.07305839	-3.3031247	3.3717144	H	5.57038516	-2.7876767	1.01324935
O	3.51800347	-4.4571792	3.35352282	H	6.23241991	-1.3412272	1.78599635
C	2.68835094	-2.6443616	4.67075417	H	5.66512063	-2.6812621	2.78084348
O	3.00344311	-3.480393	0.97570798	H	0.82909753	-3.5523477	1.33975577

conformer 6a_6							
C	-1.1392529	3.29443144	-0.2885199	C	4.48378414	-3.0775897	2.88645899
C	0.87799401	1.69858931	0.98376098	H	-2.0950835	1.51392655	0.37128364
C	1.24797522	3.11084772	0.47664706	O	-0.2661684	-3.2590707	1.32208207
C	0.03366946	3.89735651	0.01928635	H	1.98582019	3.06012218	-0.3333042
C	-0.2667318	-0.4912912	0.43431646	H	-0.7459629	-1.0691231	-0.3631504
C	1.03002909	-1.2781316	0.81277959	H	2.94332865	1.47455636	1.78854827
C	2.18001375	-0.4290645	1.30120457	H	0.34586659	-1.9357199	2.76329993
C	2.0991052	0.90604001	1.40240563	H	2.91099112	-1.8249342	3.60436171
C	0.74991555	-2.3898844	1.85093775	H	3.84119826	-1.6123045	0.69790156

C	2.00831432	-3.2145117	2.207595	H	4.21169693	-0.5101044	2.01505871
C	3.17958921	-2.303503	2.65281809	H	-1.9722804	2.18178367	-2.4055274
C	3.44019414	-1.1875644	1.62748782	H	-0.2367384	0.88310324	-3.337276
C	0.00275125	0.95876527	-0.0407162	H	-2.5597871	-0.6429903	-1.3435407
C	-1.3034282	1.76498515	-0.3448549	H	-3.6916146	0.68528211	-1.2656351
C	-1.687515	1.32403889	-1.7853379	H	-4.6961504	1.64308505	-3.3317215
N	-0.4487589	0.83868972	-2.3468114	H	-5.3969268	1.17200462	-5.6509475
C	0.59081342	0.85776822	-1.4409229	H	-4.3959503	-0.723068	-6.8984759
O	1.7632265	0.75627287	-1.7567439	H	-2.6831503	-2.1535647	-5.8160892
C	-2.8325462	0.29846995	-1.8292615	H	-1.9672658	-1.6979978	-3.497686
C	-3.2836037	0.00264792	-3.2421234	H	-3.2862512	3.50466222	-0.2501923
C	-4.247684	0.80754676	-3.8642556	H	-2.440522	5.01833984	-0.0234178
C	-4.6465089	0.5454826	-5.1757787	H	-2.5022465	4.28093628	-1.6409158
C	-4.0842792	-0.5193489	-5.877398	H	1.20533832	5.60489516	-0.5544708
C	-3.1224511	-1.3234005	-5.2689355	H	-0.5150369	5.8890553	-0.6903387
C	-2.7223871	-1.0638491	-3.9575812	H	0.23444707	5.84506304	0.91620884
C	-2.4056711	4.07172212	-0.5714569	H	2.46267501	4.46216599	1.17924303
C	0.24183458	5.38978203	-0.0790506	H	-1.5163237	-1.388065	1.63130627
O	1.84745766	3.81944592	1.56926293	H	1.03303111	-4.5984097	5.30561723
H	0.29934346	1.82988167	1.91213142	H	1.36549652	-2.9003283	4.94009736
O	-1.2151736	-0.464364	1.50981676	H	-0.1164053	-3.6849137	4.30540524
H	1.36964896	-1.7457923	-0.1236118	H	2.76984187	-4.7923732	1.33826355
C	1.68745622	-4.2549186	3.30377678	H	4.82815637	-3.5756196	1.97420797
O	2.0584411	-5.4268091	3.16473378	H	5.27888416	-2.3994652	3.21558049
C	0.94147805	-3.8230012	4.53931403	H	4.36248153	-3.8366047	3.6652879
O	2.37511799	-3.9514738	1.02085768	H	0.20244915	-3.8425909	0.68670389
conformer 6a_7							
C	-1.0858146	2.99853812	-0.9519438	C	5.25202763	-2.9377098	1.69590451
C	1.19876993	1.58910022	0.04242222	H	-1.8114939	1.33881025	0.16595294
C	1.41901549	2.91230389	-0.7245141	O	0.36852036	-3.4711577	0.94220369
C	0.11285468	3.62521236	-1.020355	H	1.97531125	2.75245437	-1.6547029
C	0.03409307	-0.6517693	0.07485095	H	-0.6304084	-1.3241247	-0.4751447
C	1.39679251	-1.4030907	0.18866767	H	3.38152544	1.46187178	0.45569652
C	2.58620009	-0.4886761	0.3684927	H	1.07918971	-2.0504144	2.25877572
C	2.48875639	0.85015345	0.32349046	H	3.75805579	-1.6968753	2.58904059
C	1.36412	-2.4886127	1.29406119	H	4.16058559	-1.6660069	-0.443542
C	2.71746691	-3.22842	1.45560166	H	4.71465355	-0.4611083	0.70933026
C	3.89028487	-2.2319429	1.63783174	H	-2.3619186	1.58911126	-2.6430449
C	3.91510074	-1.1868665	0.51332868	H	-0.8221873	0.20534192	-3.7606244
C	0.13746094	0.71632339	-0.6461434	H	-3.2818948	-0.7373454	-2.5962657
C	-1.22627	1.47640199	-0.7492364	H	-2.6191942	-1.004492	-1.0137167
C	-1.9180512	0.83187489	-1.9872901	H	-3.7960467	-0.2504824	0.95404782
N	-0.8232635	0.27381963	-2.7488742	H	-5.8183304	0.76763196	1.93291303
C	0.39350584	0.42099617	-2.115998	H	-7.427873	1.98663487	0.49272544
O	1.46613996	0.27595131	-2.6737681	H	-7.0113974	2.17968232	-1.9441653
C	-2.9980057	-0.2168353	-1.6711981	H	-4.996924	1.16572847	-2.9456851

C	-4.2445213	0.38550695	-1.0692107	H	-3.1367586	3.30142655	-0.3632499
C	-4.4926119	0.27884107	0.3060886	H	-2.3073888	4.79088504	-0.739353
C	-5.6339506	0.8549758	0.86516264	H	-2.800212	3.71266427	-2.0597169
C	-6.5380589	1.54038645	0.05626258	H	1.12583392	5.19285743	-2.08983
C	-6.3032007	1.64900496	-1.3129055	H	-0.5971616	5.46466152	-1.9561913
C	-5.1623209	1.07413052	-1.8744209	H	0.43568476	5.69932392	-0.5315167
C	-2.4003779	3.74360675	-1.0423363	H	1.66455285	4.02917615	0.86296563
C	0.27020738	5.07557374	-1.4153945	H	0.04261719	-0.0005148	1.93258752
O	2.2122293	3.77647668	0.10054145	H	1.78289601	-5.9139567	1.94502209
H	0.82474028	1.86605718	1.04110228	H	3.57069312	-5.9002577	2.11271246
O	-0.5817819	-0.4853682	1.36044481	H	2.53655734	-6.0729286	3.54462117
H	1.54175343	-1.9063239	-0.779203	H	2.0526084	-4.3618484	0.03233056
C	2.59793331	-4.0969774	2.73089961	H	5.46918521	-3.4755731	0.76714035
O	2.47435183	-3.5925898	3.8511403	H	6.05516944	-2.2097836	1.8548963
C	2.62402937	-5.594729	2.56479365	H	5.29354298	-3.6546275	2.52178557
O	2.93665602	-4.0225016	0.28507028	H	-0.4942542	-3.0695869	1.16863581
conformer 6a_8							
C	-2.354825	2.12734392	-1.0019798	C	6.20630646	-0.5883971	0.3198943
C	0.46861352	1.82997365	-0.6431674	H	-2.0805853	0.70546754	0.55524017
C	-0.0432682	2.86287947	-1.6725539	O	1.86444161	-2.762446	1.37889509
C	-1.5470482	3.04361351	-1.5877624	H	0.23244784	2.59729728	-2.6999981
C	0.28656526	-0.5551719	0.18575221	H	-0.1908725	-1.5249499	0.01822546
C	1.8110449	-0.7919668	-0.0407486	H	2.57855774	2.48692547	-0.9554061
C	2.57102683	0.44986435	-0.4448346	H	2.3018224	-0.8771446	2.09503453
C	1.96930971	1.62544577	-0.6903266	H	4.6344006	0.34424634	1.42873038
C	2.47269789	-1.4688752	1.18681873	H	4.21326289	-0.376598	-1.5140948
C	3.99687873	-1.6882244	1.00797621	H	4.54452306	1.21672228	-0.8522666
C	4.71251847	-0.3752974	0.60128787	H	-3.3879197	-0.0146617	-1.9004701
C	4.05563785	0.25897628	-0.6327813	H	-1.7846044	-1.1225298	-3.2177703
C	-0.3232348	0.51505128	-0.7533227	H	-3.3115321	-2.4039535	-1.1464388
C	-1.8495819	0.75702014	-0.5138968	H	-2.223404	-1.9942938	0.14340712
C	-2.539815	-0.3865738	-1.3145258	H	-3.0642144	-1.1052935	2.22582176
N	-1.549751	-0.7761349	-2.2942619	H	-5.006711	-0.5254907	3.6306179
C	-0.3620069	-0.0913324	-2.1478577	H	-7.2649324	-0.3298886	2.62425805
O	0.50303389	-0.0453361	-3.0044825	H	-7.5785265	-0.7245318	0.19625162
C	-3.0247123	-1.5817408	-0.4763695	H	-5.6499275	-1.3091179	-1.2278675
C	-4.2128829	-1.2481215	0.39291971	H	-4.117107	1.94914342	0.22596015
C	-4.0484692	-1.0273223	1.76714258	H	-4.0539529	3.45087577	-0.6609381
C	-5.1438414	-0.6968225	2.56593465	H	-4.4453785	1.94848748	-1.520675
C	-6.4125103	-0.5861431	2.00051452	H	-1.5373226	4.51152431	-3.1596945
C	-6.5879228	-0.8073859	0.63602892	H	-3.1232617	4.27357208	-2.4633223
C	-5.4940012	-1.1373936	-0.1651243	H	-1.8947566	5.16960488	-1.546717
C	-3.8222486	2.38423413	-0.7346479	H	1.36915969	4.18360699	-1.8982416
C	-2.059055	4.31892365	-2.2154428	H	0.47175931	0.59578593	1.77324832
O	0.55427171	4.12895128	-1.3712201	H	5.39215503	-3.7522956	3.5143917
H	0.28812577	2.26644215	0.35217215	H	4.23406745	-4.2677749	2.27165804

O	0.00508247	-0.2333909	1.5552302	H	5.88049584	-3.7209684	1.80844384
H	1.88256703	-1.484627	-0.8928859	H	3.46945719	-3.3267556	0.1198743
C	4.54274562	-2.1515763	2.38014627	H	6.36653155	-1.2770013	-0.5163033
O	4.5612045	-1.4000212	3.3595362	H	6.68840014	0.36198732	0.06584229
C	5.04345981	-3.5686028	2.49406	H	6.72264163	-0.9920363	1.19640287
O	4.19206094	-2.6771125	-0.0084869	H	1.00290828	-2.5886647	1.80916517

Table S7. Important thermodynamic parameters of the B3LYP-D3(BJ)/6-311G* optimized conformers of **1a** in the gas phase

Conformers	C[a](Hartree)	E[b](Hartree)	G[c](Hartree)
1a_10	0.565275	-1668.726258	-1668.160983
1a_7	0.564920	-1668.721431	-1668.160121
1a_9	0.564836	-1668.724784	-1668.159948
1a_1	0.563665	-1668.722845	-1668.159180
1a_4	0.563411	-1668.722587	-1668.159176
1a_2	0.562082	-1668.721177	-1668.159095
1a_8	0.564031	-1668.723115	-1668.159084
1a_5	0.562724	-1668.721431	-1668.158707
1a_12	0.563580	-1668.722130	-1668.158550
1a_3	0.563554	-1668.721435	-1668.157880
1a_6	0.563658	-1668.721431	-1668.157137
1a_11	0.561775	-1668.717682	-1668.155907

[a]Thermal correction to Gibbs free energy; [b]Electronic energy; [c]Gibbs free energy (E + C).

Table S8. Conformational analysis of the B3LYP-D3(BJ)/6-311G* optimized conformers of **1a** in the gas phase (T=298.15 K)

Conformers	ΔG (kcal/mol)[a]	Population[b]
1a_10	0.000000	39.63%
1a_7	0.540908	15.89%
1a_9	0.649466	13.23%
1a_1	1.131388	5.86%
1a_4	1.133898	5.84%
1a_2	1.184726	5.36%
1a_8	1.191628	5.30%
1a_5	1.428197	3.55%
1a_12	1.526715	3.01%
1a_3	1.947142	1.48%
1a_6	2.413377	0.67%
1a_11	3.185205	0.18%

[a]The relative Gibbs free energy; [b]The Boltzmann distribution of each conformer.

Table S9. Important thermodynamic parameters of the B3LYP/6-311G* optimized conformers of **2a** in the gas phase

Conformers	C[a](Hartree)	E[b](Hartree)	G[c](Hartree)
2a_3	0.561115	−1671.078096	−1670.516981
2a_2	0.559030	−1671.075902	−1670.516872
2a_1	0.560529	−1671.076880	−1670.516352
2a_4	0.561076	−1671.077314	−1670.516238
2a_7	0.559563	−1671.075752	−1670.516190
2a_8	0.559917	−1671.075183	−1670.515266
2a_6	0.559284	−1671.073025	−1670.513741
2a_5	0.557378	−1671.070645	−1670.513268

[a]Thermal correction to Gibbs free energy; [b]Electronic energy; [c]Gibbs free energy (E + C).

Table S10. Conformational analysis of the B3LYP/6-311G* optimized conformers of **2a** in the gas phase (T=298.15 K)

Conformers	ΔG (kcal/mol)[a]	Population[b]
2a_3	0.0000000	28.52%
2a_2	0.0683978	25.41%
2a_1	0.3946994	14.64%
2a_4	0.4662347	12.98%
2a_7	0.4963549	12.33%
2a_8	1.0761676	4.63%
2a_6	2.0331097	0.92%
2a_5	2.3299186	0.56%

[a]The relative Gibbs free energy; [b]The Boltzmann distribution of each conformer.

Table S11. Important thermodynamic parameters of the B3LYP/6-311G* optimized conformers of **3a** in the gas phase

Conformers	C[a](Hartree)	E[b](Hartree)	G[c](Hartree)
3a_4	0. 5921770	−1823. 7932322	−1823. 2010550
3a_2	0. 5923070	−1823. 7932337	−1823. 2009260
3a_1	0. 5948740	−1823. 7952335	−1823. 2003600
3a_3	0. 5948770	−1823. 7952335	−1823. 2003560
3a_5	0. 5916870	−1823. 7932322	−1823. 1985720
3a_6	0. 5912700	−1823. 7932322	−1823. 1979380
3a_7	0. 5937350	−1823. 7911947	−1823. 1974600
3a_8	0. 5886950	−1823. 7834799	−1823. 1947850

[a]Thermal correction to Gibbs free energy; [b]Electronic energy; [c]Gibbs free energy (E + C).

Table S12. Conformational analysis of the B3LYP/6-311G* optimized conformers of **3a** in the gas phase (T=298.15 K)

Conformers	ΔG (kcal/mol)[a]	Population[b]
3a_4	0. 000000	33. 79%
3a_2	0. 080948	29. 47%
3a_1	0. 436115	16. 17%
3a_3	0. 438625	16. 11%
3a_5	1. 558090	2. 43%
3a_6	1. 955927	1. 24%
3a_7	2. 255873	0. 75%
3a_8	3. 934444	0. 04%

[a]The relative Gibbs free energy; [b]The Boltzmann distribution of each conformer.

Table S13. Important thermodynamic parameters of the B3LYP/6-311G* optimized conformers of **4a** in the gas phase

Conformers	C[a](Hartree)	E[b](Hartree)	G[c](Hartree)
4a_1	0.616836	−1863.104872	−1862.488036
4a_4	0.618558	−1863.105978	−1862.48742
4a_3	0.617827	−1863.104635	−1862.486808
4a_2	0.614872	−1863.101431	−1862.48656
4a_5	0.61573	−1863.101164	−1862.485434
4a_6	0.6176	−1863.100993	−1862.483393

[a]Thermal correction to Gibbs free energy; [b]Electronic energy; [c]Gibbs free energy (E + C).

Table S14. Conformational analysis of the B3LYP/6-311G* optimized conformers of **4a** in the gas phase (T=298.15 K)

Conformers	ΔG (kcal/mol)[a]	Population[b]
4a_1	0.000000	48.25%
4a_4	0.386542	25.12%
4a_3	0.770574	13.13%
4a_2	0.926194	10.10%
4a_5	1.632763	3.06%
4a_6	2.913496	0.35%

[a]The relative Gibbs free energy; [b]The Boltzmann distribution of each conformer.

Table S15. Important thermodynamic parameters of the B3LYP/6-311G* optimized conformers of **5a** in the gas phase

Conformers	C[a](Hartree)	E[b](Hartree)	G[c](Hartree)
5a_4	0.645210	−1902.421409	−1901.776199
5a_1	0.644777	−1902.420877	−1901.776100
5a_3	0.641933	−1902.417132	−1901.775200
5a_7	0.645770	−1902.420382	−1901.774612
5a_2	0.644733	−1902.418849	−1901.774115
5a_8	0.643599	−1902.420382	−1901.773501
5a_6	0.643022	−1902.416296	−1901.773275
5a_5	0.642424	−1902.415373	−1901.772949

[a]Thermal correction to Gibbs free energy; [b]Electronic energy; [c]Gibbs free energy (E + C).

Table S16. Conformational analysis of the B3LYP/6-311G* optimized conformers of **5a** in the gas phase (T=298.15 K)

Conformers	ΔG (kcal/mol)[a]	Population[b]
5a_4	0.000000	37.35%
5a_1	0.062123	33.63%
5a_3	0.626875	12.96%
5a_7	0.995847	6.95%
5a_2	1.307716	4.10%
5a_8	1.693003	2.14%
5a_6	1.834819	1.68%
5a_5	2.039385	1.19%

[a]The relative Gibbs free energy; [b]The Boltzmann distribution of each conformer.

Table S17. Important thermodynamic parameters of the B3LYP/6-311G* optimized conformers of **6a** in the gas phase

Conformers	C[a](Hartree)	E[b](Hartree)	G[c](Hartree)
6a_9	0. 531742	−1594. 601499	−1594. 069757
6a_1	0. 530085	−1594. 599328	−1594. 069243
6a_2	0. 529057	−1594. 598179	−1594. 069122
6a_3	0. 530369	−1594. 597597	−1594. 067228
6a_5	0. 529503	−1594. 596613	−1594. 067110
6a_10	0. 530592	−1594. 597539	−1594. 066947
6a_4	0. 531255	−1594. 598039	−1594. 066783
6a_6	0. 530253	−1594. 596916	−1594. 066662
6a_7	0. 528903	−1594. 594854	−1594. 065952
6a_8	0. 529554	−1594. 594252	−1594. 064698

[a]Thermal correction to Gibbs free energy; [b]Electronic energy; [c]Gibbs free energy (E + C).

Table S18. Conformational analysis of the B3LYP/6-311G* optimized conformers of **6a** in the gas phase (T=298.15 K)

Conformers	ΔG (kcal/mol)[a]	Population[b]
6a_9	0. 000000	42. 14%
6a_1	0. 322537	24. 44%
6a_2	0. 398464	21. 50%
6a_3	1. 586955	2. 89%
6a_5	1. 661000	2. 55%
6a_10	1. 763283	2. 14%
6a_4	1. 866194	1. 80%
6a_6	1. 942122	1. 59%
6a_7	2. 387649	0. 75%
6a_8	3. 174538	0. 20%

[a]The relative Gibbs free energy; [b]The Boltzmann distribution of each conformer.
